# Impact of phosphorus fertilizer level on the yield and metabolome of goji fruit

**DOI:** 10.1038/s41598-020-71492-y

**Published:** 2020-09-04

**Authors:** Feng Wei, Zhigang Shi, Ru Wan, Yunxiang Li, Yajun Wang, Wei An, Ken Qin, Youlong Cao, Xiaoyi Chen, Xiuying Wang, Libin Yang, Guoli Dai, Jiayue Feng

**Affiliations:** 1 Wolfberry Engineering Research Institute, Ningxia Academy of Agriculture and Forestry Sciences, National Wolfberry Engineering Research Center, Yinchuan, 750002 Ningxia China; 2 College of Horticulture, Northwest A & F University, Yangling, 712000 Shan Xi China; 3 Ningxia State Farm A & F Technology Central, Yinchuan, 750002 Ningxia China

**Keywords:** Metabolomics, Secondary metabolism

## Abstract

Goji (*Lycium barbarum* L.) is a highly medicinal value tree species. The yield and nutritional contents of goji fruit are significant affected by fertilizer level. In this study, we analyzed the yield and nutritional contents change of goji fruit, which planted in pot (vermiculite:perlite, 1:2, v:v) in growth chamber under P0 (32.5 g/per tree), P1 (65 g/per tree), and P2 (97.5 g/per tree). Meanwhile, we utilized an integrated Ultra Performance Liquid Chromatography–Electrospray Ionization–Tandem Mass Spectrometry (UPLC–ESI–MS/MS) to analysis of the response of the metabolome in goji fruit to phosphorus level. The results show that the yield of goji fruits had strongly negative correlation with phosphorus level, especially in the third harvest time. The amino acids, flavonoids, polysaccharides, and betaine contents of goji fruits in the first harvest time had obvious correlated with the level of phosphorus level. The Kyoto Encyclopedia of Genes and Genomes (KEGG) enrichment results indicated that the impact of different phosphorus fertilizer levels on each group mainly involved the biosynthesis of flavonoids. The results provide new insights into the theoretical basis of the relationship between the nutritional contents of goji fruits and phosphorus fertilizer level.

## Introduction

*Lycium barbarum* L., well known as goji or wolfberry, is an economic tree species belonging to the Solanaceae family. It is widely distributed in the arid and semi-arid areas of northwestern China, Southeastern Europe, and the Mediterranean areas^1,2^. In China, there are seven species and three varieties that are mainly cultivated in the northwest and northern parts of the country^3^. Because its fruit is rich in nutrients, such as amino acids, polysaccharides, and flavonoids etc., the goji fruit is also called a “super fruits”, and has become popular around the world, with a rapid sequence of new products entering a dynamic and further growing market^4^. Phosphorus plays an important role in the growth and metabolism of plants and is also an important component of the plant protoplasm as well as nucleic acids and nuclear proteins^5,6^. As a structural element and regulatory factor in plants, phosphorus is involved in the metabolic pathways of the three major metabolites of plants, and as an enzymatic reaction substrate, it is involved in the reaction process of photosynthesis and respiration as well as in the regulation of enzyme activity, ensuring the normal growth and development of plants. Phosphorus is also an important role in the inner energy transfer of plant, such as phosphate esters. Most of phosphate esters are intermediates of biosynthesis and metabolic degradation. Their function and formation are directly related to energy metabolism and energy-rich phosphate. For example, the energy required for starch biosynthesis or ion absorption is composed of an energy-rich intermediate or coenzyme (mainly ATP). When Energy liberated during glycolysis, respiration, or photosynthesis is utilized for the synthesis of the energy-rich pyrophosphate bond, and on hydrolysis of this bond − 30 kJ per mole ATP are released. This energy can be transferred with the phosphoryl group in a phosphorylation reaction to another compound which results in the activation (priming reaction) of this compound. In some phosphorylation reactions the energy-rich inorganic pyrophosphate (PP_i_) is liberated, which takes place in all of the major biosynthetic pathways, such as acylation of CoA in fatty acid synthesis, formation of APS in sulphate activation and so on^7–10^.

A Previous study reported that as the yield of dry goji fruit was 1605–1978.5 kg/ha, which needs nitrogen, phosphorus, and potassium at 4.85, 8.2, and 1.8 kg, respectively, per 50 kg of dry goji fruit^11,12^. Some researchers also reported that the yield of dry goji fruits reached 4,440–8,355 kg/ha, needing nitrogen, phosphorus, and potassium at 10.95, 5.4, and 1.75 kg, respectively, per 50 kg of dry goji fruit^13,14^. Wang et al. indicated that the accumulation of nitrogen, phosphorus, and potassium in dry goji fruits at different harvest times was parabolic, and that the peak of nutrient accumulation in goji fruits occurred in July^15^. The above studies showed that the yield of dry goji fruits was positively correlated with the amount of applied fertilizer. Cai et al. studied the effects of N, P, and K on the contents of sugar and carotene in goji fruits and found that the contents of polysaccharides, sugars, and carotenoids increased with increases in N, P, and K up to a certain level, beyond which they no longer increased^16^. Liu et al., through their research on the fruit quality of goji in Ningxia under different phosphorus application levels, found that the transverse diameter of goji showed a trend of first decreasing and then increasing with the application of increasing amounts of phosphorus^17^. Wang et al. reported that the application of organic fertilizers (cow dung, sheep dung, pig dung, and biogas slurry) significantly increased the transpiration rate, stomatal conductance, and intercellular CO_2_ concentration of leaves, significantly increased the chlorophyll content and photosynthesis of leaves in goji, and that sheep manure had the greatest effect in increasing the 100-grain weight, soluble solid solution, soluble sugar, and vitamin C of goji fruits^18^. Some researchers by comparing appearance traits (fruit color, diameter, 100-grain weight, and grain size) of organic and non-organic fresh goji fruits from different harvest times, indicated that the nutritional component contents were higher in goji fruits picked in July^19,20^.

Although there have been a large number of reports about the influence of fertilizer on the yield and quality of goji fruits, and some research has also studied the relationship between harvest time and the quality of goji fruit, details about the impact of different phosphorus fertilizer levels on the yield and quality of goji fruits from different harvest times is still unclear. In the present study, we analyzed the yield changes in fresh and dry fruits in response to different phosphorus fertilizer levels for different harvest times and also studied the linear correlation between the nutritional contents of goji fruits and the level of phosphorus fertilizer. Finally, we utilized an integrated UPLC–ESI–MS/MS detection system to study the change of metabolite profiling under different phosphorus fertilizer levels in order to provide a theoretical basis for the regulation of the yield and nutrients of goji fruits by fertilization.

## Materials and methods

### Plant materials

The seedlings variety was called ‘0901’, which provided from the National Wolfberry Engineering Research Center, Ningxia, China.

### Experimental design and conditions

The experiment was implemented at the phytotron of National Wolfberry Engineering Research Center, Ningxia, China. The temperature ranging from 25 to 27 °C, a relative humidity ranging from 70 to 80% and illumination intensity range from 110 to 125 μmol m^−2^ s^−1^ (14 h light, and 10 h dark.). There were sixteen seedlings assigned to each treatment, which planted in pots (diameter: 25 cm, height: 50 cm) containing culture medium (vermiculite: perlite, 1:2, v: v), respectively. The details of each treatment are shown in Table 1. The nitrogen, phosphorus, and potassium were supplied by urea (N 46%), ammonium diacid phosphate (N 12%, P_2_O5 61%), and potassium sulfate (K_2_O 52%), respectively.

**Table 1:** Experimental treatments.

Treatment	N (g/per tree)	P (g/per tree)	K (g/per tree)
P0	85	32.5	50
P1	85	65	50
P2	85	97.5	50

### Determination of nutritional contents of the goji fruit

The sample was prepared according to a method as described previous study^21^. The amino acids, total flavonoid, polysaccharide, and betain in goji fruits were measured according to a method as described previously^21^. The mass spectrometry of the amino acids is shown in Supplementary Figs. S1 and S2.

### The untargeted metabolomics assays of goji fruits

The HPLC conditions and ESI-Q TRAP-MS/MS according to a method as described previous study^21,22^. Qualitative and quantitative analyses of metabolites followed the methods of Wang^23^. Based on the self-built database MWDB (Metware Biotechnology Co., Ltd. Wuhan, China) and the public database of metabolite information, qualitative analyses of the primary and secondary spectral data of mass spectrometry were performed. The stacking diagram of TIC maps from QC mass spectrometry is shown in Supplementary Fig. S3.

### Statistical analysis

Statistical software was performed using Microsoft Office Excel 2013 and SPSS 20.0 (IBM Corporation, Armonk, NY, USA), Graphpad Prism 7.0 (GraphPad Software, Inc., 7,825 Fay Avenue, Suite 230, La Jolla, CA 92,037 USA), and R (https://www.r-project.org/)^24^.

## Results

### Yield of Goji Fruits for Different Phosphorus level

**Figure 1:**
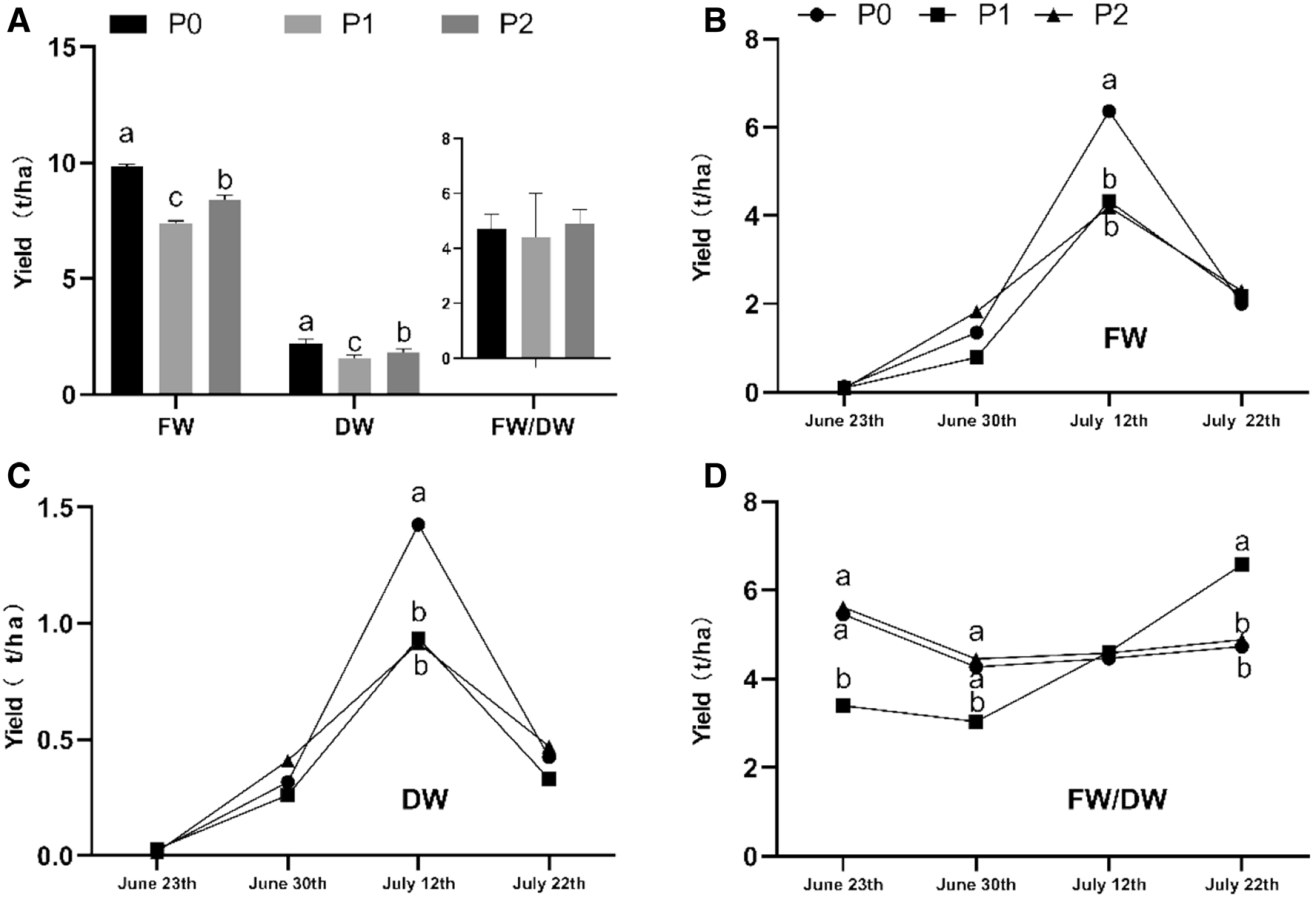
Yield of goji fruits under different levels of phosphorus fertilization. (**A**) Total yield of goji fruit; (**B,C**) yield of goji fresh fruit (FF) and dry fruit (DF) from each harvest. (**D**) Ratio of goji fruit fresh weight (FW) and dry weight (DW). Small letters indicate a significant difference (*p* < 0.05), as analyzed by Duncan’s multiple tests; values without letters means no significant difference.

The impact of different phosphorus fertilizers on the yield of goji fruits is shown in Fig. 1. The total yields of goji fresh fruit (FF) and dry fruit (DF) significantly differed for P0, P1, and P2 (Fig. 1A), whereby P0 had the highest yields and P1 the lowest. However, the difference was not significant in FW/DW (p < 0.05). Cause goji fruits can be harvested four or five times from early June to mid-August depending on the climatic conditions^3^. So, we analyzed the effects of different phosphorus fertilizations on the yield of goji fresh fruit and dry fruit for each harvest (Fig. 1B,C). As the graph shows, we harvested the goji fruits on June 23rd, June 30th, July 12th, and July 22nd. We found that significant differences in the total yield of goji fruits was due to differences observed at the third harvest. Thus, we analyzed the correlation of phosphorus level with the yield of goji fruits by bivariate correlation analysis and found that the total yield of fresh fruits (FF) and dry fruits (DF) was not correlated with phosphorus fertilizer level, but the yield of fresh fruits (FF) and dry fruits (DF) was negative and showed a strong linear correlation (− 0.98, − 0.89) for the first and third harvest (June 23th, July 12th) in addition to a positive correlation (0.99) for the fourth harvest (July 22th). Figure 1D shows the ratio between the weight of fresh fruit (FW) and weight of dry fruit (DW), which means that one kilogram of dry fruits requires a certain number of kilograms of fresh fruit. Although the FW/DW ratios of goji fruits were significantly different between the first, second, and fourth harvest, there was still no correlation between FW/DW and phosphorus fertilization.

### Nutritional contents of fresh goji fruits under different levels of phosphorus fertilization

**Figure 2:**
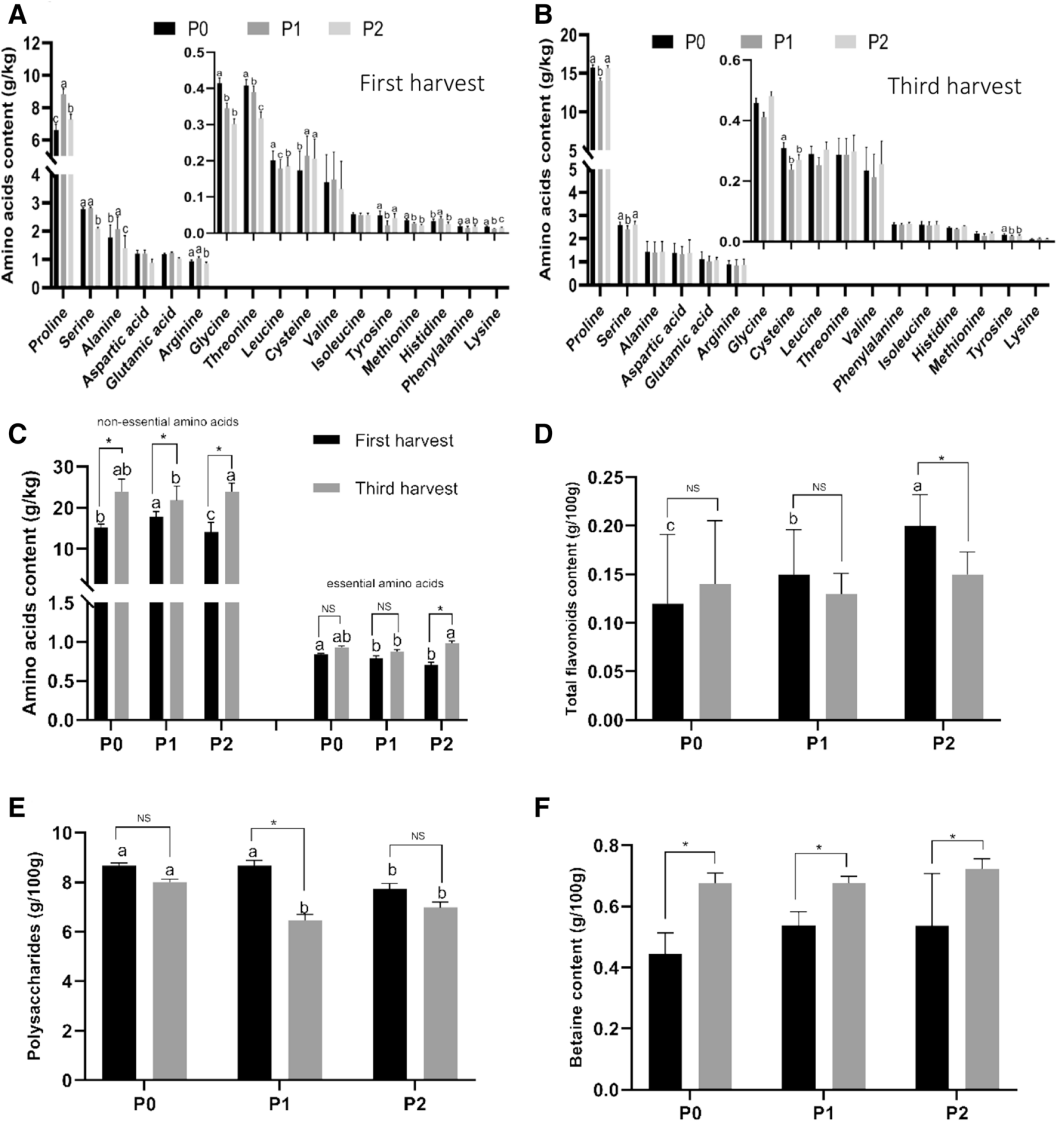
The nutritional contents of fresh goji fruits. (**A,B**) Amino acids content of goji fruits under different phosphorus level; (**C**) non-essential amino acids and essential amino acids total content of goji fruits; (**D,E,F**) Total flavonoids, polysaccharides, and betaine content of goji fruits. Small letters indicate a significant difference between P0, P1, and P2 (*p* < 0.05), as analyzed by Duncan’s multiple tests; values without letters mean no significant difference. * means a significant difference between first harvest and third harvest (*p* < 0.05), as analyzed by Duncan’s multiple tests; NS means no significant difference.

Because the yields of P0, P1, and P2 were significantly different due to differences at the third harvest and most customers think that goji fruits from the first harvest—also called “tou cha”—are best because it can reach the highest value of commodity, we determined the main nutritional contents in fresh goji fruits of the first harvest and third harvest for each treatment (Fig. 2, and Table S1). Seventeen kinds of amino acids were detected in the fruits of P0, P1, and P2, among which 6 essential amino acids and 11 non-essential amino acids were found. The total content of non-essential amino acids showed significant differed between goji fruits of the first and third harvests for each treatment, in which the goji fruits of the third harvest had higher contents than those of the first harvest. The contents of essential amino acids did not significantly differ in the fruits of the first and third harvest under P0 and P1, but were found to differ for P2. We also found a significant difference in non-essential amino acids between the first and third harvest under P0, P1, and P2 due to Pro, and the significant difference in essential amino acids under P2 was due to differences in Phe. There were also significant differences in non-essential amino acids (Serine, Glycine, Histidine, Arginine, Alanine, Proline, Tyrosine, and Methionine) and essential amino acids (Valine, Threonine, Leucine, Phenylalanine, and Lysine) under P0, P1, and P2. The flavonoid contents of goji fruits did not significantly differ between the first and third harvests under P0, P1, and P2, but showed significant differences between P0, P1, and P2 for the first harvest, where the flavonoid content of fruits increased with increasing levels of phosphorus fertilizer. The polysaccharide contents of fruits showed significant divergence between the first and third harvest for both P1 and P2. Regardless of the first harvest or the third harvest, there were also significant differences in the polysaccharide contents of fruits between the three different phosphorus applications P0, P1, and P2. There was significant divergence in the betaine content of fruits between the first and third harvest under P0, P1, and P2, with the fruits of the third harvest having higher contents than for the first harvest, but for fruits which the same harvest, there was no significant difference between P0, P1, and P2 for fruits of either the first or third harvest.

### Correlations between nutritional contents of fresh goji fruits and phosphorus fertilizer levels

We found the nutritional contents of fresh goji fruits were significantly affected by phosphorus fertilizer levels for different harvest times (Fig. 2, and Table S1). Thus, we further analyzed the relationships between the nutritional contents of fresh goji fruits and phosphorus at different harvest times using bivariate correlation analysis (Table 2). Some non-essential amino acids (Serine, Glycine, Arginine, and Methionine), essential amino acids (Threonine and Isoleucine), and polysaccharides of goji fruits were observed to have a highly negative correlation with phosphorus fertilizer levels at the first harvest. However, there were no strong linear correlations with phosphorus at the third harvest. The betaine levels of goji fruits had a significant positive linear correlation with phosphorus in both the first and third harvest, and flavonoid levels had strong highly linear correlations with phosphorus only in the first harvest.

**Table 2:** The correlation between the nutritional contents of fresh goji fruits and phosphorus.

	Phosphorus	
	First harvest	Third harvest
	(June 23rd)	(July 12th)
Aspartic acid (Asp)	− 0.77	− 0.05
Glutamic acid (Glu)	− 0.39	− 0.56
Cysteine (Cys)	0.76	− 0.55
Serine (Ser)	− 0.85	0.14
Glycine (Gly)	− 0.99	0.31
Histidine (His)	− 0.53	0.44
Arginine (Arg)	− 0.86	− 0.72
Alanine (Ala)	− 0.56	0.17
Proline (Pro)	0.28	− 0.03
Tyrosine (Tyr)	− 0.25	− 0.65
Methionine (Met)	− 0.99	− 0.13
Valine (Val)	− 0.71	0.46
Threonine (Thr)	− 0.94	0.82
Isoleucine (Ile)	− 0.87	− 0.40
Leucine (Leu)	− 0.71	0.27
Phenylalanine (Phe)	− 0.24	0.65
Lysine (Lys)	− 0.50	0.33
Flavonoid	0.99	0.50
Polysaccharide	− 0.87	− 0.66
Betaine	0.85	0.87

Correlation is indicated by the Pearson’s correlation coefficient.

### Metabolite profiling of fresh goji fruits for different phosphorus fertilization levels and KEGG enrichment analysis

**Figure 3:**
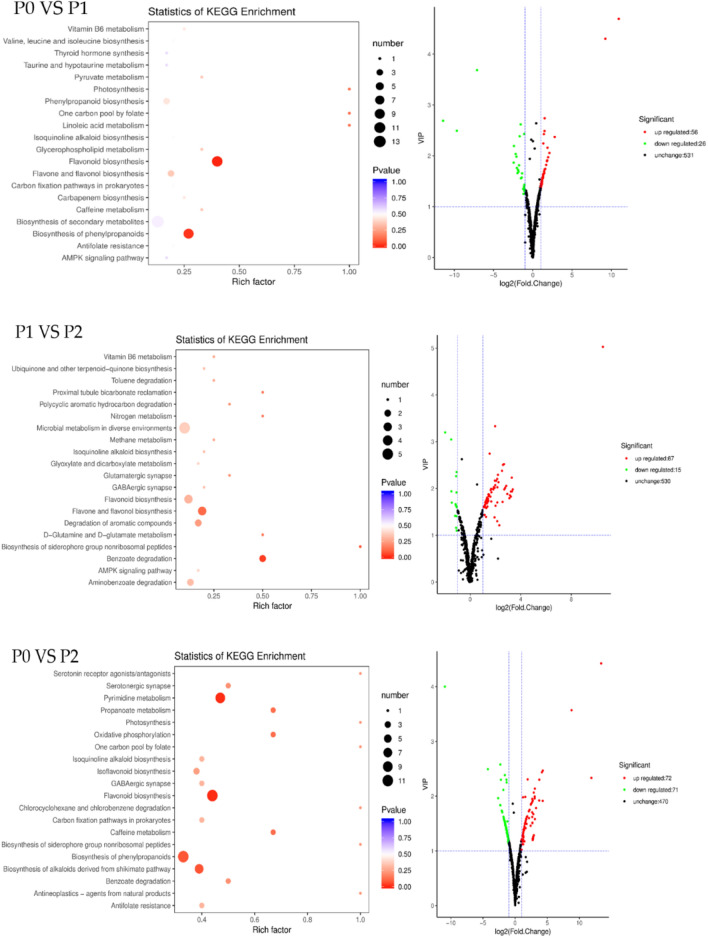
Enrichment analysis of Kyoto Encyclopedia of Genes and Genomes (KEGG) and volcanic map of differential metabolites. The *p*-value represents the degree of enrichment, and the closer the *p*-value is to 0, the more significant the enrichment. The size of the point represents the number of differential metabolites.

We found that in most cases, the nutritional content of fresh goji fruits in the first harvest were strongly correlated with phosphorus fertilizer levels (Table 2), and in order to study the impact of phosphorus fertilizer levels on the secondary metabolites of goji fruits, they were investigated for P0, P1, and P2 of the first harvest using UPLC–ESI–MS/MS, and the results were compared with databases. The results showed that 612 metabolites were identified from three treatments (Table S2), and these results corresponded with those of a previous study^21^. Comparing P0 with P1, 82 metabolites had a significant change, including 56 upregulated and 26 downregulated metabolites. Comparing P1 to P2, there were also 82 metabolites with an obvious change, including 67 upregulated and 15 downregulated metabolites. About 143 metabolites had changes when comparing P0 to P2. All Kyoto Encyclopedia of Genes and Genomes (KEGG)^25^ enrichment classification results indicated that the impact of different phosphorus fertilizer levels on each group mainly involved the biosynthesis of flavonoids and phenylpropanoids (Fig. 3).

### Metabolic profiling of flavonoids in goji fruits for different phosphorus fertilizer levels

**Figure 4:**
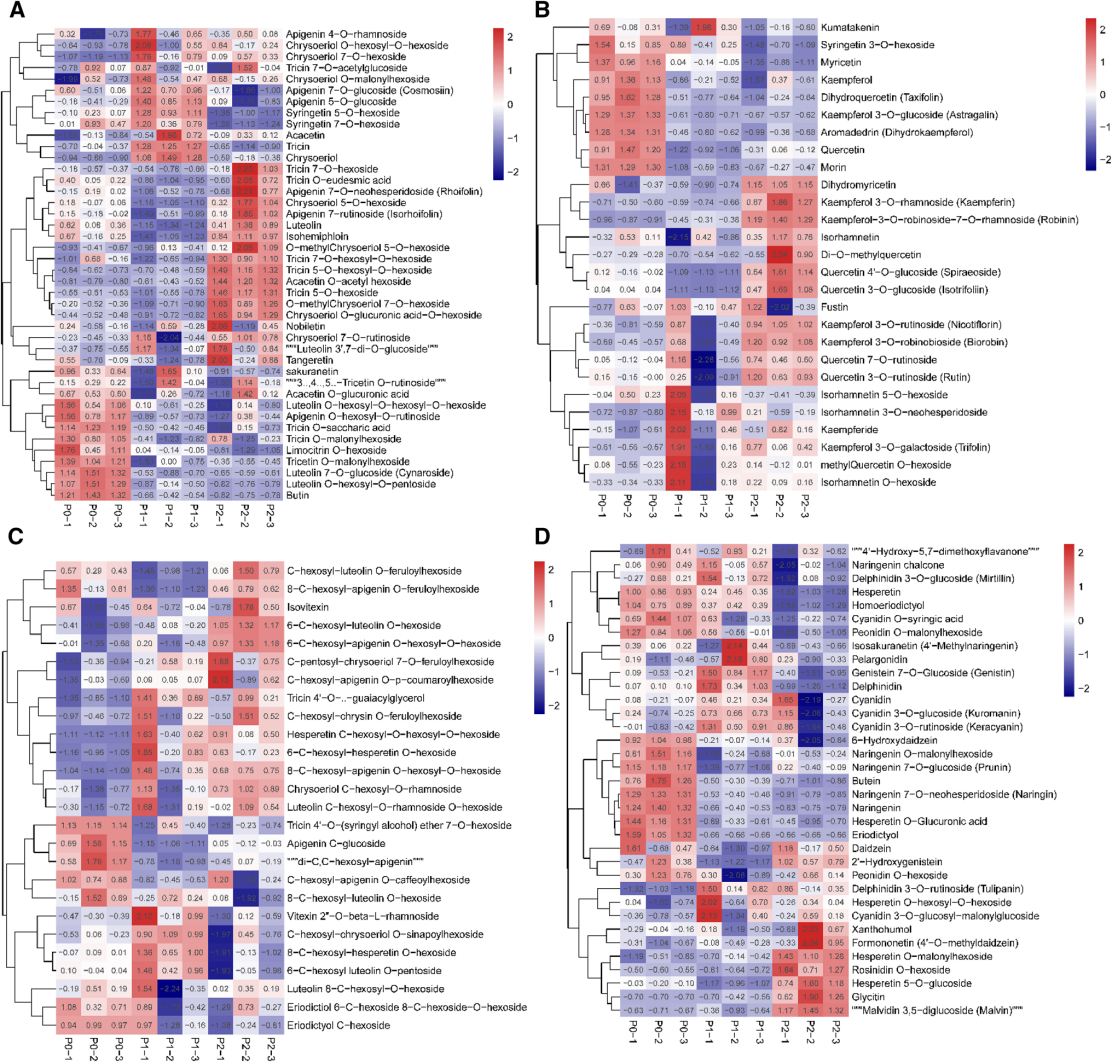
Heat map of flavonoids of goji fruits. (**A**) Flavone of goji fruits; (**B**) Flavonol of goji fruits; (**C**) Flavonolignan, flavone C-glycosides, and flavanone of goji fruits; (**D**) Isoflavone and anthocyanins of goji fruits. One column for each sample, and one row for each metabolite. The amount of each metabolite is represented by a bar of a specific color. The up-regulated and down-regulated metabolites are indicated by different shades of red and blue, respectively.

The KEGG enrichment classification results indicated that the impact of different phosphorus fertilizer levels on each group (P0 vs. P1, P1 vs. P2, and P0 vs. P2) mainly involved the biosynthesis of flavonoids (Fig. 3), and the flavonoid contents of goji fruits from the first harvest were strongly positively correlated with phosphorus fertilizer levels (Table 2). Thus, we analyzed the change in metabolites of the flavonoid biosynthesis pathway of goji fruits (Fig. 4). There were 117 flavonoids found in goji fruits, including 42 flavone, 27 flavonol, 2 flavonolignan, 24 flavone C-glycosides, 16 flavanone, and 6 isoflavone compounds. There were also 13 anthocyanins in goji fruits. The flavone of goji fruits had six metabolites which were significantly upregulated with increasing phosphorus fertilizer levels (Fig. 4A), which were tricin 7-O-hexosyl-O-hexoside, tricin 5-O-hexosyl-O-hexoside, acacetin O-acetyl hexoside, tricin 5-O-hexoside, O-methylchrysoeriol 5-O-hexoside, and chrysoeriol O-glucuronic acid-O-hexoside, and nine metabolites were obviously downregulated (luteolin O-hexosyl-O-hexosyl-O-hexoside, apigenin O-hexosyl-O-rutinoside, tricin O-saccharic acid, tricin O-malonylhexoside, limocitrin O-hexoside, tricetin O-malonylhexoside, luteolin 7-O-glucoside, luteolin O-hexosyl-O-pentoside, and butin). The flavonols of goji fruits included seven downregulated metabolites (Fig. 4B), which were myricentin, kaempferol, dihydroquercetin, kaempferol 3-O-glucoside, aromaderdrin, and morin. The flavone C-glycosides had three metabolites (hesperetin C-hexosyl-O-hexosyl-O-hexoside, c-pentosyl-chrysoeriol 7-O-feruloylhexoside, and c-hexosyl-apigenin O-p-coumaroylhexoside) which significantly increased with the increase in phosphorus fertilizer levels (Fig. 4C). Four flavanones of goji fruits were affected by phosphorus fertilizers, namely naringin, naringenin, eriodictyol, and butein, which decreased with increasing phosphorus fertilizer levels (Fig. 4C). The isoflavones of goji fruits included one upregulated metabolite (glycitin), and one downregulated metabolite (6-hydroxydaidzein) as phosphorus fertilizer levels increased (Fig. 4D). There were also two metabolites of anthocyanins (rosinidin O-hexoside, and tulipanin) in goji fruits which were significantly upregulated with increasing phosphorus fertilizer levels (Fig. 4D).

### Metabolic profiling of polysaccharides and alkaloids in goji fruits

**Figure 5:**
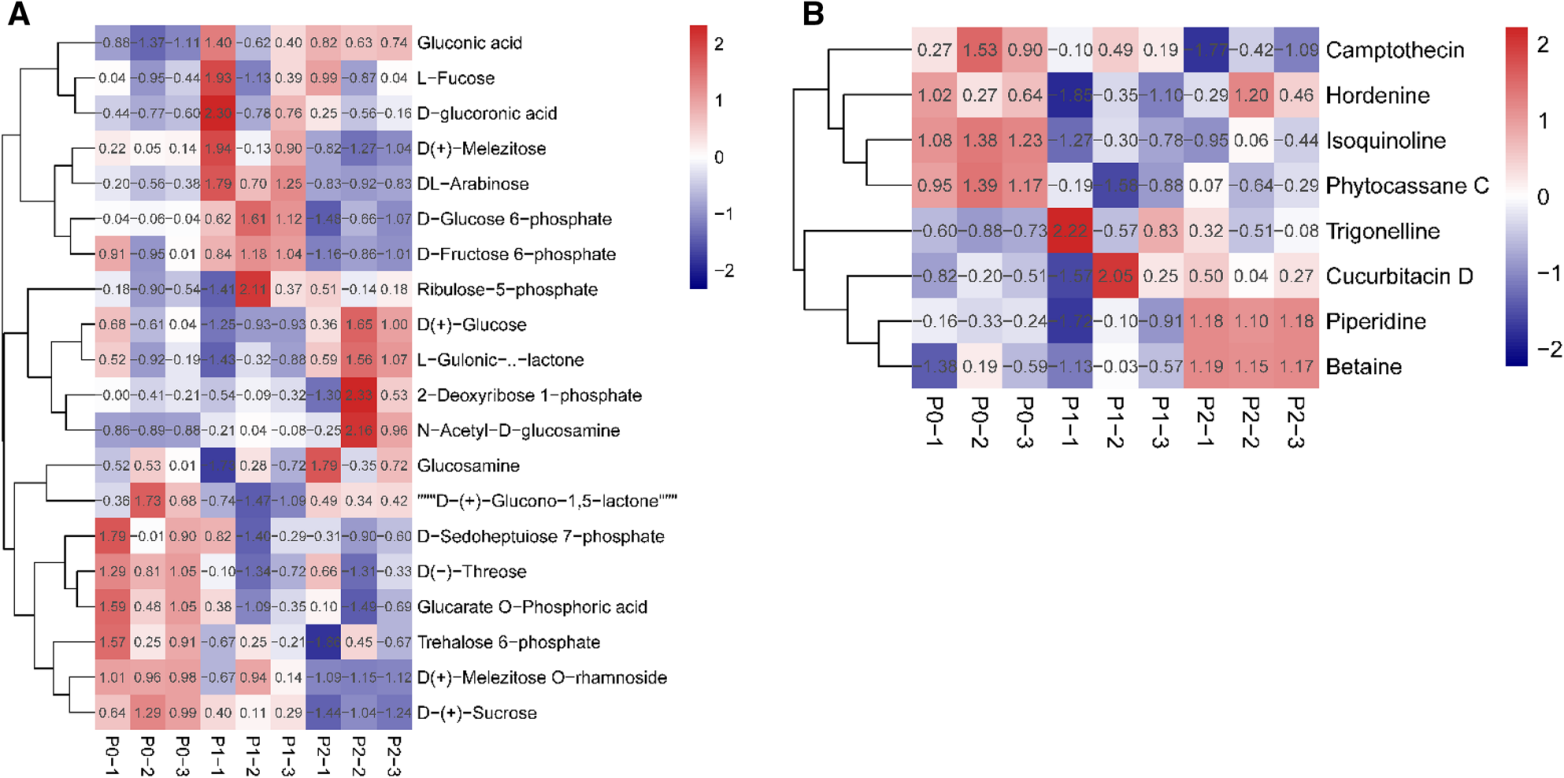
Heat map of carbohydrates and alkaloids in goji fruits. (**A**) Carbohydrates of goji fruits; (**B**) alkaloids of goji fruits. One column for each sample, and one row for each metabolite. The amount of each metabolite is represented by a bar of a specific color. The up-regulated and down-regulated metabolites are indicated by different shades of red and blue, respectively.

The contents of polysaccharides and alkaloids also had a significant linear correlation with the levels of phosphorus fertilizer (Table 2). There were 20 carbohydrates of goji fruits in each treatment under the first harvest (Fig. 5A), with only three monosaccharides contributing to the polysaccharide composition (DL-arabinose, L-fucose, and glucosamine), and the three monosaccharides showed no significant change with the increase in phosphorus fertilizers. However, D (−)-threose was downregulated with the increase in the level of phosphorus fertilizer (Fig. 5A). We also found six alkaloids and two terpenoids of goji fruits in P0, P1, and P2 under the first harvest (Fig. 5B). According to the VIP values and fold change, the metabolic profiling of alkaloids and terpenoids showed no significant change with phosphorus fertilizers in each group (P0 vs. P1, P1 vs. P2, and P0 vs. P2), except betaine.

## Discussion

In the present study, the yields of goji fruits under different phosphorus fertilizer levels at different harvest times were analyzed, and we found that the total yield of goji fresh fruit (FF) and dry fruit (DF) were both highest in P0 and lowest in P2. Shi et al. reported that the correlation between yield and various fertilizer factors and the first-order coefficient showed a larger effect for potassium, followed by phosphorus and then nitrogen^26^. The goji is an economic tree species with continuous flowering and fruiting from summer to autumn, and can be harvested four to five times per year^15,27–29^. Thus, each harvest of goji is very important for the total yield, and there has so far been no research analyzing the yield differences according to different harvest times and different levels of fertilizer. Here, we found that the yield of fresh fruits (FF) and dry fruits (DF) had a negative and strong linear correlation (− 0.98, − 0.89) with phosphorus fertilizer levels for the first and third harvests, and a positive correlation (0.99) for the fourth harvest. Furthermore, the significant difference in the total yield of goji fruits for different phosphorus fertilizer levels was due to differences in the third harvest. However, the results of this study are different from those of previous studies which indicated that fertilization had a positive effect on the yield of goji^11–14,30^. We think the third harvest time is key for the yield of goji fruit, where low phosphorus at this time is beneficial for yield. Meanwhile, we studied the ratio of goji fruit fresh weight (FW) and dry weight (DW) in three treatments under different harvest times. FW/DW means the number of kilograms of fresh fruits needed for one kilogram of dry fruit. Yuan et al. set up a comprehensive evaluation system for fresh goji fruit drying process indicators, which indicated that the 100-grain dry weight and tissue moisture content are the key indicators that reflect the processing characteristics of dried fresh goji fruit^31^. Although there were significant differences in the FW/DW in the first, second, and fourth harvest under different phosphorus level, this still had no significant impact on the total FW/DW of goji. We infer that the FW/DW may be affected by climatic conditions of cultivated areas and the characteristics of the specific variety.

The amino acids, polysaccharides, betaines, flavonoids, anthocyanins, and other functional ingredients in goji fruits have been shown to enhance human immunity, inhibit the growth of tumor cells, delay aging, contribute to fatigue resistance, lower blood pressure, protect the liver, protect vision, and provide antioxidant activity^1,32^. By characterizing the yield of fruits in response to P0, P1, and P2, a significant difference was observed for the third harvest. Therefore, we detected the main nutritional contents of fresh goji fruits in P0, P1, and P2 from the first harvest and third harvest. Meanwhile, we analyzed the metabolic profiles of nutritional contents in goji fruit from the first harvest using an integrated UPLC–ESI–MS/MS detection system. In the present study, the first and third harvests of goji fruit corresponding P0, P1, and P2 treatments presented 17 amino acids, among which six essential amino acids and 11 non-essential amino acids were found. The results were consistent with previous studies, which detected 17 kinds of amino acids from summer and autumn fruits of ‘Jingqi No.1’, ‘Jingqi No.2’, ‘Ningqi No.1’, ‘Ningqi No.5’ and ‘Ningqi No.7’^3,21,30,33–35^. The essential and non-essential amino acids levels were both higher for all three treatments in the third harvest than compared to the first harvest. The results corresponded with those of a previous report, which indicated that the dry goji fruits harvested in July and October had the highest nutritional contents; the fresh goji fruits harvested in summer are easier to eat fresh, and in autumn, they are suitable for making dry fruits^30,31^. Meanwhile, the total contents of amino acids in goji fruits increased with the phosphorus level for a range of phosphorus level of 32.5–65 g/plant. A previous study reported that the increasing phosphorus level inhibits Cd accumulation and promotes the synthesis of amino acids in plants, but the correlation between each amino acid and phosphorus level has not been clarified^36^. We found that there were significant negative linear correlations between contents of amino acids (Ser, Gly, His, Arg, Ala, Pro, Tyr, Met, Val, Thr, Leu, Phe, and Lys) and phosphorus fertilizers. We infer that phosphorus may affect the conversion between amino acids, but how it affects the conversion between amino acids requires further study.

The flavonoid contents of goji fruits showed no significant difference between the first harvest and third harvest for P0, P1, and P2. However, a significant difference in fruits was observed between P0, P1, and P2 for the first harvest, where the flavonoid contents of fruits in the first harvest were positively correlated with phosphorus level (0.99). Wang et al. reported that the flavonoid content of the goji was highly correlated with altitude (r = 0.914, *p* < 0.01) and average diurnal temperature (r = 0.851, *p* < 0.05). Thus, the divergence of flavonoid contents in each harvest time may be caused by phosphorus level and diurnal temperature^3,15,27^. The flavonoid biosynthetic pathway is initiated by the catalytic action of phenylalanine ammonia lyase (PAL) on the precursor amino acid phenylalanine, and then cinnamate 4-hydroxylase (C4H) enzyme, leading to the production of the entry compound to flavonoid biosynthesis, chalcone. Chalcone was catalyzed by chalcone isomerase to naringenin, which is the main product of metabolism and then enters other different metabolic pathways^37–39^. We analyzed the metabolic profiling of flavonoids and found 117 metabolites (42 flavones, 27 flavonols, 2 flavonolignans, 24 flavone C-glycosides, 16 flavanones, and 6 isoflavones) and 13 anthocyanins in goji fruits. The flavanones of goji fruits included four metabolites (naringin, naringenin, eriodictyol, and butein) which were significantly downregulated and others that exhibited no change. There were also some metabolites of flavone, flavonol, flavonolignan, flavone C-glycoside, isoflavone, and anthocyanin in goji fruits which were upregulated or downregulated. We infer that the change in flavonoids may be initiated by the divergence of phenylalanine.

*Lycium barbarum* polysaccharide (LBP) is a glycoprotein complex in which sugar chains (glucose, arabinose, galactose, mannose, xylose, and rhamnose) account for 70% of its total content^2,31,40^. We found that LBP was significantly negatively correlated with phosphorus level (− 0.87). However, Zhang et al. indicated that the correlation between total sugar and available potassium (0.608) was positive, and polysaccharides were positively correlated with available potassium (0.626)^41^. Their descriptions are different from the results presented here, which may mean that the samples were different. Our samples were from the first harvest and third harvest, but their samples were mixed year-round. A previous study also provides an opinion, which show that potassium deficiency can lead the root respiration per fresh or dry matter increased, but decreased by deficiency of either phosphorus or all nutrients^42^. So, the LBP may be will be influenced by phosphorus fertilizer level. But, through the metabolic profiling of carbohydrates, we found only three monosaccharides (DL-arabinose, L-fucose, and glucosamine) belonging to LBP and no change with changing phosphorus level was observed. Betaine is a small molecule belonging to the active components of goji^43,44^. According to the metabolic profiling of alkaloids and terpenoids, we found six alkaloids and two terpenoids of goji fruits. Only betaine showed a significant change, which had a strong positive correlation with phosphorus level. Chung et al. indicated that the synthesis of betaine in goji was highly related to nitrogen levels, with a decrease in betaine concentrations with increasing N fertilizer^45^. Thus, we can infer that the betaine of goji had a positive correlation with phosphorus level and negative correlation with nitrogen level.

In conclusion, the yield of goji fruits under different harvest times had a highly negative correlation with phosphorus fertilizer levels, especially in the third harvest time. The amino acids, flavonoids, polysaccharides, and betaine contents of goji fruits in the first harvest time were significantly affected by phosphorus level. The data of the metabolic profiling of goji fruits showed that the phosphorus fertilizer levels mainly affected the conversion between amino acids and the biosynthesis of flavonoids.

## Supplementary information

Supplementary information 1.

Supplementary information 2.
