# Supplementary material for: Impact of phosphorus fertilizer level on the yield and metabolome of goji fruit
Source: Sci Rep. 2020 Sep 4;10:14656. doi: 10.1038/s41598-020-71492-y (PMC7474080; doi:10.1038/s41598-020-71492-y)
Supplement: Supplementary file 1 — Supplementary information 1. [file 41598_2020_71492_MOESM1_ESM.docx]

**Impact of phosphorus fertilizer level on the Yield and Metabolome of Goji Fruit**

**Feng Wei ^2, 3, †^,** **Zhigang Shi** **^1, †,^ *, Ru Wan** **^1, †^, Yunxiang Li ^1^,** **Yajun Wang ^1^, Wei An ^1^, Ken Qin ^1^, Youlong Cao ^1^, Xiaoyi Chen ^2^, Xiuying Wang ^1^, Libin** **Yang ^1^, Guoli Dai ^1^, Jiayue Feng ^2^**

^1^ Wolfberry Engineering Research Institute, Ningxia Academy of Agriculture and Forestry Sciences, National Wolfberry Engineering Research Center, Yinchuan 750002, China.

^2^ College of Horticulture, Northwest A & F University, Yangling, Xianyang 712000, China.

^3^ Ningxia State Farm A & F Technology Central, Yinchuan, Ningxia 750002, China.

***** Correspondence: shizhigang76@163.com; Tel.: +86-0951-6886733

Figure S1: The mass spectrometry of the amino acids of goji fruit in first harvest; Figure S2: The mass spectrometry of the amino acids of goji fruit under in third harvest;

Figure S3: Tic of positive (A) and negative (B) ion multiple reaction monitoring (MRM);

Table S1: The statistical analysis in nutritional contents of fresh goji fruits under different phosphorus levels;

Table S2: Metabolites of goji fruits under different phosphorus fertilizations.
