# Supplementary material for: Impact of phosphorus fertilizer level on the yield and metabolome of goji fruit
Source: Sci Rep. 2020 Sep 4;10:14656. doi: 10.1038/s41598-020-71492-y (PMC7474080; doi:10.1038/s41598-020-71492-y)
Supplement: Supplementary file 2 — Supplementary information 2. [file 41598_2020_71492_MOESM2_ESM.docx]

**
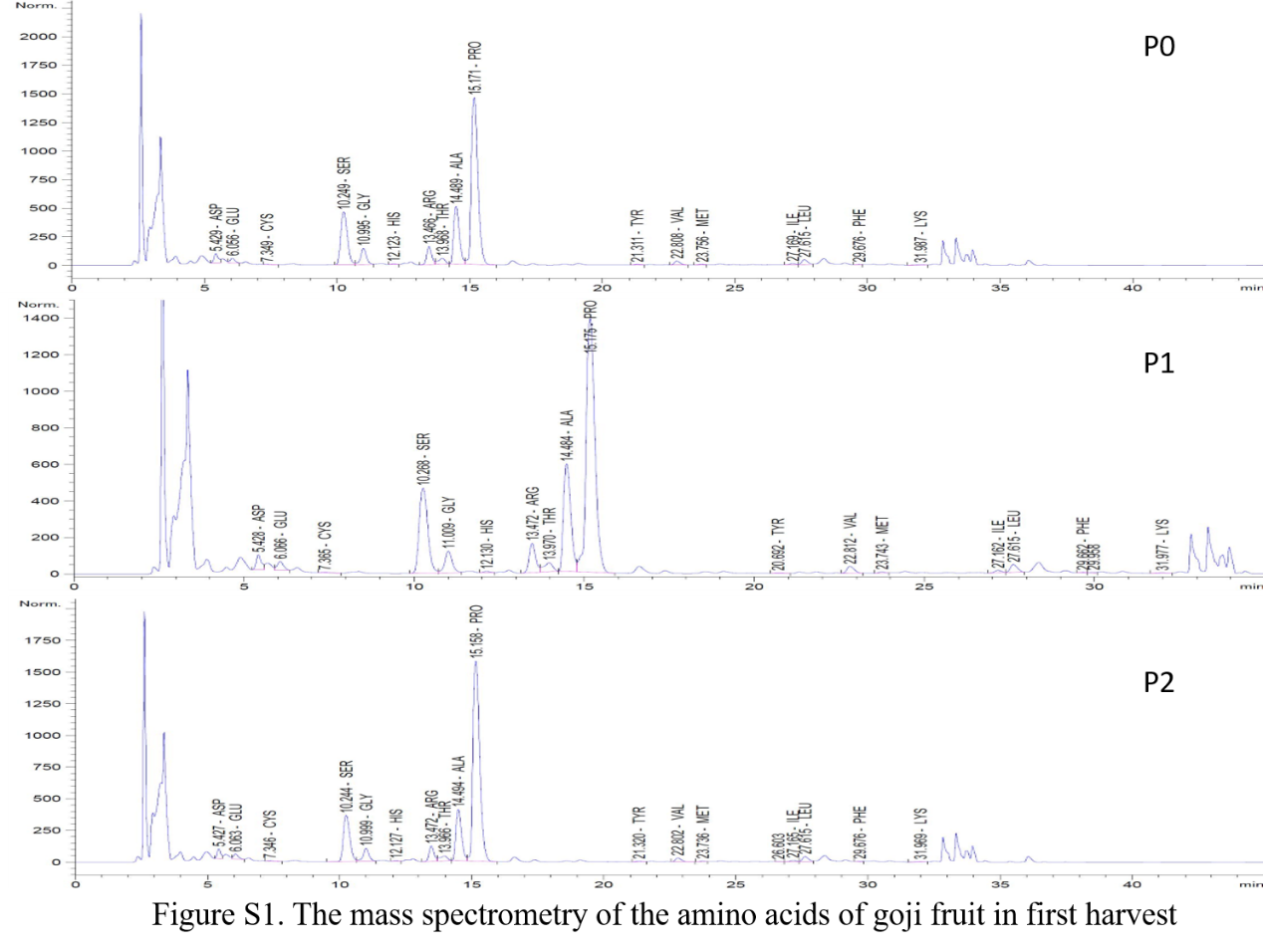
**

**
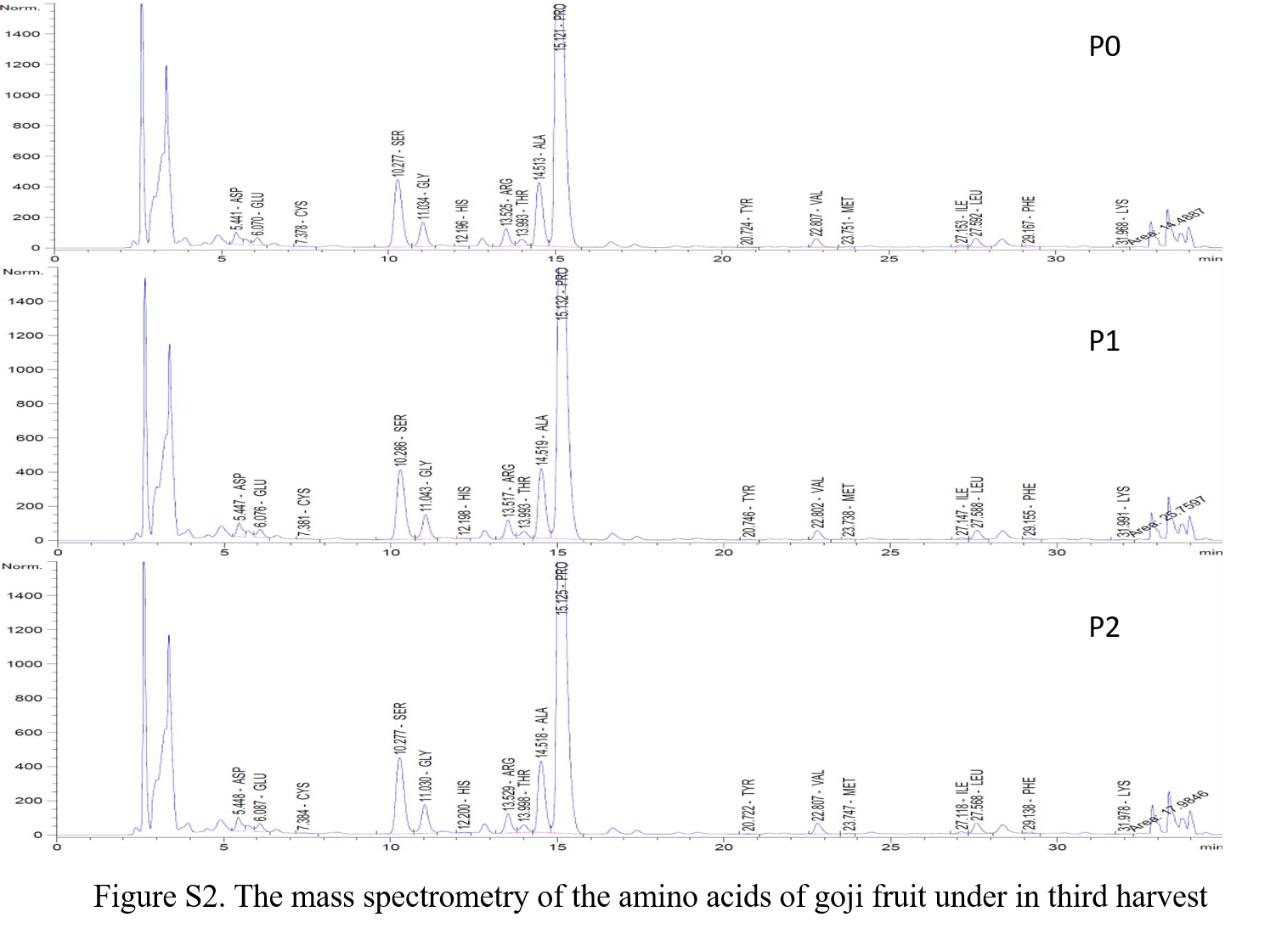
**

**
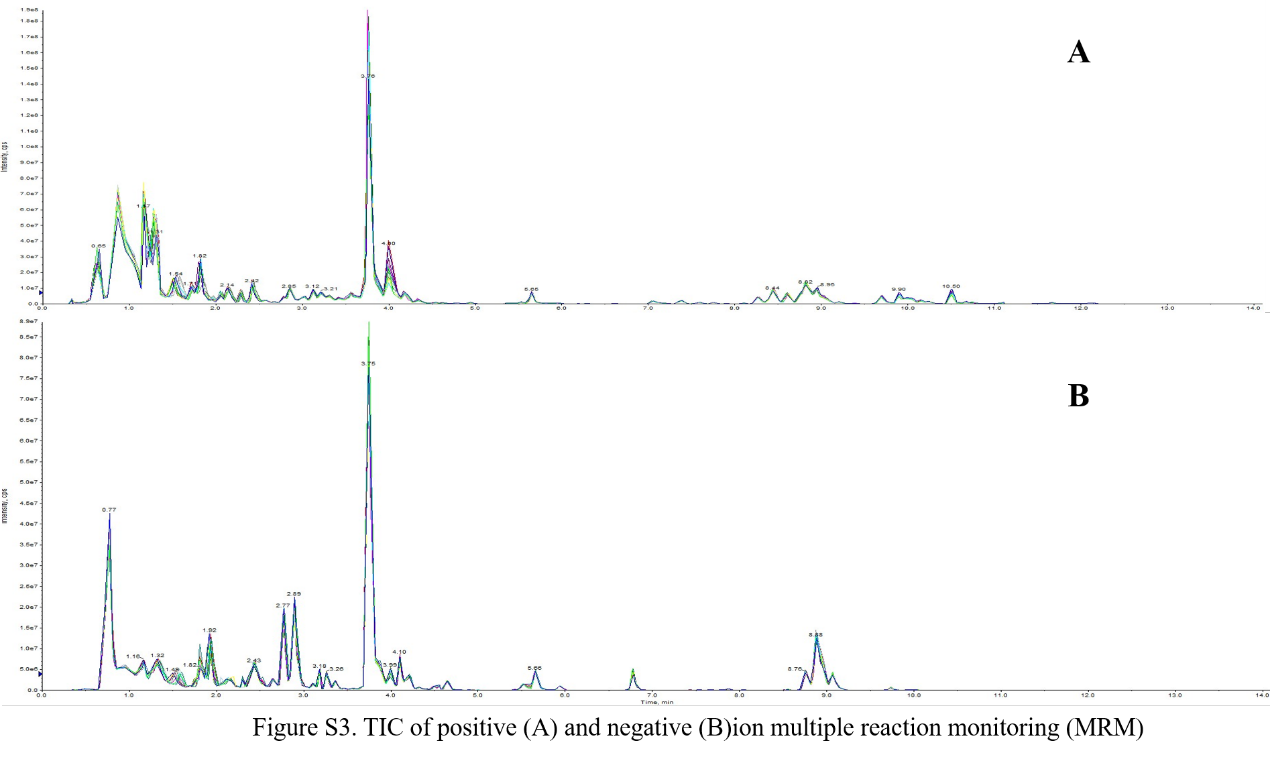
**

**Table S1.** The statistical analysis in nutritional contents of fresh goji fruits under different phosphorus levels

|  | P0 | | P1 | | P2 | |
| --- | --- | --- | --- | --- | --- | --- |
|  | First harvest | Third harvest | First harvest | Third harvest | First harvest | Third harvest |
|  | (June 30^th^) | (July 12^th^) | (June 30^th^) | (July 12^th^) | (June 30^th^) | (July 12^th^) |
| Aspartic acid (Asp) (g/kg) | 1.195±0.028 | 1.394 ± 0.963* | 1.231 ± 0.051 | 1.340 ± 0.737 | 1.032 ± 0.051 | 1.391 ± 0.763* |
| Glutamic acid (Glu) (g/kg) | 0.930 ± 0.051 | 1.120 ± 0.012 | 1.044 ± 0.050 | 1.030 ± 0.076 | 0.857 ± 0.043 | 1.069 ± 0.026* |
| Cysteine  (Cys) (g/kg) | 0.173 ± 0.054b | 0.310 ± 0.006a* | 0.214 ± 0.023a | 0.238 ± 0.003b | 0.206 ± 0.010a | 0.270 ± 0.054b |
| Serine  (Ser) g/kg) | 2.781 ± 0.046a* | 2.579 ± 0.230a | 2.805 ± 0.025a | 2.410 ± 0.455b | 2.092 ± 0.061b | 2.609 ± 0.128a* |
| Glycine  (Gly) (g/kg) | 0.414 ± 0.035a | 0.459 ± 0.056 | 0.345 ± 0.025b | 0.412 ± 0.098 | 0.301 ± 0.061b | 0.481 ± 0.073* |
| Histidine  (His) (g/kg) | 0.033 ± 0.006b | 0.046 ± 0.004* | 0.040 ± 0.002a | 0.041 ± 0.002 | 0.025 ± 0.002b | 0.050 ± 0.002* |
| Arginine  (Arg) (g/kg) | 1.206 ± 0.120a* | 0.900 ± 0.066 | 1.210 ± 0.245a* | 0.847 ± 0.296 | 0.883 ± 0.046b | 0.860 ± 0.487 |
| Alanine  (Ala) (g/kg) | 1.777 ± 0.441b* | 1.434 ± 0.298 | 2.072 ± 0.322a* | 1.416 ± 0.876 | 1.394 ± 0.227c | 1.438 ± 0.036 |
| Proline  (Pro) (g/kg) | 6.627 ± 0.332b | 15.734 ± 1.222a* | 8.823 ± 1.211a | 14.047 ± 0.832b* | 7.263 ± 0.598c | 15.682 ± 0.332a* |
| Tyrosine  (Tyr) (g/kg) | 0.049 ± 0.012a* | 0.023 ± 0.002a | 0.022 ± 0.007b | 0.017 ± 0.001b | 0.042 ± 0.023a* | 0.019 ± 0.002b |
| Methionine (Met) (g/kg) | 0.035 ± 0.003a | 0.027 ± 0.002 | 0.027 ± 0.012b | 0.020 ± 0.002 | 0.022 ± 0.004b | 0.026 ± 0.001 |
| Total (g/kg) | 15.220 ± 0.784b | 24.026 ± 2.981a* | 17.833 ± 1.229a | 21.818 ± 3.457b* | 14.117 ± 2.335c | 23.895 ± 2.129a* |
| Valine  (Val) (g/kg) | 0.141 ± 0.076b | 0.236 ± 0.022* | 0.148 ± 0.120a | 0.213 ± 0.005* | 0.122 ± 0.044c | 0.256 ± 0.045* |
| Threonine  (Thr) (g/kg) | 0.408 ± 0.170a* | 0.288 ± 0.087 | 0.390 ± 0.112b* | 0.287 ± 0.034 | 0.318 ± 0.065c | 0.298 ± 0.056 |
| Isoleucine  (Ile) (g/kg) | 0.052 ± 0.005 | 0.055 ± 0.012 | 0.050 ± 0.007 | 0.054 ± 0.003 | 0.050 ± 0.005 | 0.055 ± 0.021 |
| Leucine  (Leu) (g/kg) | 0.202 ± 0.065a | 0.290 ± 0.001a | 0.179 ± 0.078c | 0.253 ± 0.003b* | 0.185 ± 0.025b | 0.304 ± 0.012a* |
| Phenylalanine (Phe) (g/kg) | 0.019 ± 0.004a | 0.057 ± 0.003* | 0.018 ± 0.003a | 0.056 ± 0.008* | 0.015 ± 0.008b | 0.059 ± 0.032* |
| Lysine  (Lys) (g/kg) | 0.018 ± 0.002a* | 0.008 ± 0.002 | 0.012 ± 0.001c | 0.011 ± 0.001 | 0.015 ± 0.004b | 0.009 ± 0.002 |
| Total (g/kg) | 0.840 ± 0.0140a | 0.934 ± 0.023ab | 0.794 ± 0.0270b | 0.874 ± 0.033b | 0.708 ± 0.032b | 0.981 ± 0.054a* |
| Flavonoid  (g/100g) | 0.120 ± 0.071c | 0.140 ± 0.065 | 0.150 ± 0.046b | 0.130 ± 0.001 | 0.200 ± 0.032a | 0.150 ± 0.023 |
| Polysaccharide  (g/100g) | 8.660 ± 0.051a | 8.010 ± 1.222a | 8.760 ± 0.071a* | 6.470 ± 2.870b | 7.740 ± 0.032b* | 6.980 ± 1.198b |
| Betaine  (g/100g) | 0.445 ± 0.069 | 0.676 ± 0.033* | 0.539 ± 0.043 | 0.677 ± 0.022* | 0.536 ± 0.172 | 0.724 ± 0.032* |

Note: Small letters indicate the analysis revealed significant differences between different phosphorus levels in the first harvest and third harvest (*p* < 0.05). * The analysis of variance of the first and third harvests in P0, P1, and P2 (*p* < 0.05). All values were analyzed by Duncan’s multiple range tests.

Table S2. Metabolites of goji fruits under different phosphorus fertilizations

| Index | Q1 (Da) | Q3 (Da) | Rt (min) | Molecular Weight (Da) | Ionization model | KEGG ID | Compounds | Class | mix01 | mix02 | mix03 | mix04 | mix05 | mix06 | P0-1 | P0-2 | P0-3 | P1-1 | P1-2 | P1-3 | P2-1 | P2-2 | P2-3 |
| --- | --- | --- | --- | --- | --- | --- | --- | --- | --- | --- | --- | --- | --- | --- | --- | --- | --- | --- | --- | --- | --- | --- | --- |
| pma0101 | 2.93E+02 | 2.35E+02 | 2.45E+00 | 2.92E+02 | [M+H]+ | - | N-Caffeoyl agmatine | Phenolamides | 4.00E+05 | 4.66E+05 | 4.99E+05 | 4.17E+05 | 4.46E+05 | 5.64E+05 | 8.48E+05 | 8.16E+05 | 8.32E+05 | 8.99E+04 | 2.94E+05 | 1.92E+05 | 1.48E+05 | 1.91E+05 | 1.70E+05 |
| pma0134 | 1.21E+02 | 7.71E+01 | 1.16E+00 | 1.20E+02 | [M+H]+ | - | D(-)-Threose | Carbohydrates | 1.39E+05 | 1.75E+05 | 1.42E+05 | 1.51E+05 | 1.67E+05 | 1.37E+05 | 1.91E+05 | 1.57E+05 | 1.74E+05 | 9.16E+04 | 3.34E+03 | 4.75E+04 | 1.46E+05 | 5.38E+03 | 7.57E+04 |
| pma0149 | 3.41E+02 | 1.79E+02 | 2.28E+00 | 3.40E+02 | [M+H]+ | C02887 | Sinapoyl malate | Organic acids | 2.43E+04 | 3.23E+04 | 2.56E+04 | 2.55E+04 | 2.40E+04 | 2.30E+04 | 1.99E+04 | 2.24E+04 | 2.12E+04 | 3.36E+04 | 2.03E+04 | 2.70E+04 | 3.10E+04 | 2.94E+04 | 3.02E+04 |
| pma0214 | 4.79E+02 | 3.17E+02 | 3.91E+00 | 4.78E+02 | [M+H]+ | - | methylQuercetin O-hexoside | Flavonol | 4.65E+06 | 4.92E+06 | 4.77E+06 | 4.40E+06 | 4.13E+06 | 4.20E+06 | 3.55E+06 | 3.26E+06 | 3.41E+06 | 4.51E+06 | 2.72E+06 | 3.62E+06 | 3.58E+06 | 3.46E+06 | 3.52E+06 |
| pma0253 | 4.77E+02 | 3.16E+02 | 5.27E+00 | 4.76E+02 | [M+H]+ | - | O-methylChrysoeriol 5-O-hexoside | Flavone | 3.28E+04 | 3.00E+04 | 5.32E+04 | 3.22E+04 | 2.42E+04 | 4.03E+04 | 2.01E+04 | 2.80E+04 | 2.41E+04 | 1.96E+04 | 3.63E+04 | 2.80E+04 | 3.61E+04 | 6.55E+04 | 5.08E+04 |
| pma0461 | 2.77E+02 | 1.57E+02 | 8.52E+00 | 2.76E+02 | [M+H]+ | - | "14,15-Dehydrocrepenynic acid" | Lipids_Fatty acids | 8.54E+05 | 8.18E+05 | 8.25E+05 | 8.31E+05 | 7.87E+05 | 7.83E+05 | 1.07E+06 | 1.07E+06 | 1.07E+06 | 9.23E+05 | 1.00E+06 | 9.62E+05 | 5.41E+05 | 1.12E+06 | 8.31E+05 |
| pma0584 | 3.08E+02 | 2.21E+02 | 1.27E+00 | 3.07E+02 | [M+H]+ | - | N-Caffeoylspermidine | Phenolamides | 5.27E+06 | 5.35E+06 | 4.76E+06 | 5.41E+06 | 4.58E+06 | 8.13E+06 | 6.16E+06 | 5.26E+06 | 5.71E+06 | 1.03E+07 | 6.56E+06 | 8.43E+06 | 7.99E+06 | 5.85E+06 | 6.92E+06 |
| pma0692 | 4.38E+02 | 1.48E+02 | 3.67E+00 | 4.37E+02 | [M+H]+ | - | "N', N''-di-p-coumaroylspermidine" | Phenolamides | 7.66E+04 | 9.30E+04 | 6.46E+04 | 7.29E+04 | 7.78E+04 | 5.88E+04 | 6.11E+03 | 1.21E+03 | 3.66E+03 | 2.05E+04 | 1.04E+04 | 1.55E+04 | 3.54E+04 | 2.56E+04 | 3.05E+04 |
| pma0791 | 5.21E+02 | 3.18E+02 | 4.50E+00 | 5.20E+02 | [M+H]+ | - | Naringenin O-malonylhexoside | Flavanone | 2.69E+05 | 2.00E+05 | 2.36E+05 | 2.20E+05 | 1.87E+05 | 2.00E+05 | 1.30E+05 | 1.42E+05 | 1.36E+05 | 8.90E+04 | 1.12E+05 | 1.01E+05 | 1.16E+05 | 1.07E+05 | 1.12E+05 |
| pma0795 | 5.51E+02 | 3.47E+02 | 4.56E+00 | 5.50E+02 | [M+H]+ | - | Tricetin O-malonylhexoside | Flavone | 1.32E+05 | 1.17E+05 | 1.30E+05 | 1.16E+05 | 9.30E+04 | 9.77E+04 | 2.04E+05 | 1.90E+05 | 1.97E+05 | 8.83E+04 | 1.49E+05 | 1.19E+05 | 1.35E+05 | 1.27E+05 | 1.31E+05 |
| pma0948 | 1.38E+02 | 7.72E+01 | 1.26E+00 | 1.37E+02 | [M+H]+ | - | Hydroxyphenethylamine | Others | 4.79E+05 | 5.77E+05 | 4.76E+05 | 5.04E+05 | 5.46E+05 | 4.99E+05 | 6.97E+05 | 6.93E+05 | 6.95E+05 | 3.45E+05 | 5.56E+05 | 4.51E+05 | 4.40E+05 | 3.09E+05 | 3.75E+05 |
| pma1108 | 4.33E+02 | 2.72E+02 | 3.78E+00 | 4.32E+02 | [M+H]+ | - | Apigenin C-glucoside | Flavone C-glycosides | 1.56E+05 | 2.68E+05 | 2.87E+05 | 2.92E+05 | 2.63E+05 | 2.08E+05 | 2.52E+05 | 2.89E+05 | 2.71E+05 | 1.75E+05 | 1.79E+05 | 1.77E+05 | 2.25E+05 | 2.18E+05 | 2.22E+05 |
| pma1116 | 3.01E+02 | 2.59E+02 | 4.13E+00 | 3.00E+02 | [M+H]+ | C10098 | Kaempferide | Flavonol | 7.21E+04 | 4.18E+04 | 7.98E+04 | 5.20E+04 | 5.73E+04 | 4.67E+04 | 6.59E+04 | 5.70E+04 | 6.15E+04 | 8.69E+04 | 5.66E+04 | 7.18E+04 | 6.24E+04 | 7.53E+04 | 6.89E+04 |
| pma1590 | 4.63E+02 | 3.01E+02 | 2.89E+00 | 4.63E+02 | Protonated | - | Peonidin O-hexoside | Anthocyanins | 3.29E+05 | 2.92E+05 | 3.04E+05 | 3.13E+05 | 3.35E+05 | 3.62E+05 | 2.71E+05 | 2.89E+05 | 2.80E+05 | 2.71E+05 | 2.25E+05 | 2.48E+05 | 2.57E+05 | 2.78E+05 | 2.68E+05 |
| pma1751 | 2.86E+02 | 1.24E+02 | 1.95E+00 | 2.85E+02 | [M+H]+ | - | Nicotinic acid-hexoside | Nicotinic acid derivatives | 5.10E+06 | 6.93E+06 | 4.16E+06 | 8.31E+06 | 7.56E+06 | 4.52E+06 | 2.83E+06 | 4.90E+06 | 3.87E+06 | 2.43E+06 | 5.82E+06 | 4.13E+06 | 2.81E+06 | 5.06E+06 | 3.94E+06 |
| pma1840 | 2.50E+02 | 1.91E+02 | 2.73E+00 | 2.50E+02 | Protonated | - | Coumaroyl choline | Cholines | 5.03E+04 | 8.79E+04 | 9.53E+04 | 6.46E+04 | 1.08E+05 | 7.67E+04 | 3.95E+04 | 3.57E+04 | 3.76E+04 | 8.69E+04 | 4.34E+04 | 6.52E+04 | 5.70E+04 | 3.96E+04 | 4.83E+04 |
| pma2172 | 2.68E+02 | 1.31E+02 | 5.94E+00 | 2.67E+02 | [M+H]+ | - | Cinnamoyl tyramine | Tryptamine derivatives | 5.72E+05 | 5.66E+05 | 5.62E+05 | 5.47E+05 | 5.07E+05 | 5.18E+05 | 5.08E+04 | 4.24E+04 | 4.66E+04 | 3.58E+04 | 7.37E+04 | 5.48E+04 | 6.36E+04 | 7.00E+04 | 6.68E+04 |
| pma2405 | 2.80E+02 | 1.78E+02 | 2.87E+00 | 2.80E+02 | Protonated | - | Feruloylcholine | Cholines | 4.80E+04 | 3.68E+04 | 4.01E+04 | 3.47E+04 | 4.18E+04 | 3.83E+04 | 1.90E+04 | 2.38E+04 | 2.14E+04 | 1.76E+04 | 1.39E+04 | 1.58E+04 | 2.89E+04 | 4.18E+04 | 3.54E+04 |
| pma2681 | 2.06E+02 | 1.32E+02 | 2.05E+00 | 2.05E+02 | [M+H]+ | C02470 | Xanthurenic acid | Organic acids | 3.30E+04 | 4.71E+04 | 4.73E+04 | 3.50E+04 | 3.38E+04 | 3.62E+04 | 4.91E+04 | 5.50E+04 | 5.21E+04 | 7.88E+04 | 6.17E+04 | 7.03E+04 | 4.57E+04 | 6.25E+04 | 5.41E+04 |
| pma2987 | 1.42E+02 | 8.29E+01 | 6.20E-01 | 1.41E+02 | [M+H]+ | C00860 | Histidinol | Others | 8.46E+07 | 7.42E+07 | 8.33E+07 | 7.42E+07 | 5.25E+07 | 5.73E+07 | 5.50E+07 | 4.74E+07 | 5.12E+07 | 4.57E+07 | 4.92E+07 | 4.75E+07 | 5.25E+07 | 5.42E+07 | 5.34E+07 |
| pma3101 | 2.56E+02 | 1.24E+02 | 8.40E-01 | 2.55E+02 | [M+H]+ | C05841 | Nicotinate ribonucleoside | Nicotinic acid derivatives | 5.43E+06 | 5.56E+06 | 6.26E+06 | 6.01E+06 | 5.53E+06 | 5.96E+06 | 1.16E+07 | 1.11E+07 | 1.14E+07 | 8.39E+06 | 7.67E+06 | 8.03E+06 | 6.76E+06 | 5.72E+06 | 6.24E+06 |
| pma3443 | 5.35E+02 | 4.87E+02 | 5.78E+00 | 5.34E+02 | [M+H]+ | - | Tricin 7-O-acetylglucoside | Flavone | 3.94E+04 | 3.67E+04 | 3.66E+04 | 3.86E+04 | 3.08E+04 | 3.12E+04 | 2.52E+04 | 3.12E+04 | 2.82E+04 | 3.10E+04 | 2.47E+04 | 2.79E+04 | 2.22E+04 | 3.33E+04 | 2.78E+04 |
| pma3606 | 2.95E+02 | 1.80E+02 | 9.22E+00 | 2.94E+02 | [M+H]+ | - | "9-Hydroxy-(10E,12Z,15Z)-octadecatrienoic acid" | Lipids_Fatty acids | 2.58E+06 | 2.32E+06 | 2.42E+06 | 2.32E+06 | 2.16E+06 | 2.31E+06 | 1.80E+06 | 1.70E+06 | 1.75E+06 | 1.22E+06 | 1.28E+06 | 1.25E+06 | 8.64E+05 | 1.15E+07 | 6.18E+06 |
| pma3649 | 1.32E+02 | 8.62E+01 | 1.01E+00 | 1.31E+02 | [M+H]+ | C00430 | 5-Aminolevulinate | Organic acids | 3.98E+07 | 5.29E+07 | 4.85E+07 | 6.44E+07 | 6.14E+07 | 7.71E+07 | 5.83E+07 | 6.47E+07 | 6.15E+07 | 5.36E+07 | 7.64E+07 | 6.50E+07 | 6.80E+07 | 7.55E+07 | 7.18E+07 |
| pma3724 | 3.69E+02 | 2.08E+02 | 3.42E+00 | 3.68E+02 | [M+H]+ | - | 1-O-Feruloyl quinic acid | Quinate and its derivatives | 7.41E+05 | 7.25E+05 | 6.42E+05 | 6.40E+05 | 6.70E+05 | 6.07E+05 | 1.48E+06 | 1.51E+06 | 1.50E+06 | 8.08E+05 | 7.59E+05 | 7.84E+05 | 4.53E+05 | 5.77E+05 | 5.15E+05 |
| pma6254 | 7.57E+02 | 7.57E+02 | 3.68E+00 | 7.56E+02 | [M+H]+ | - | C-hexosyl-apigenin O-caffeoylhexoside | Flavone C-glycosides | 1.13E+05 | 1.02E+05 | 7.08E+04 | 1.07E+05 | 8.13E+04 | 7.55E+04 | 1.45E+05 | 1.37E+05 | 1.41E+05 | 9.23E+04 | 1.03E+05 | 9.77E+04 | 1.50E+05 | 6.74E+04 | 1.09E+05 |
| pma6270 | 2.58E+02 | 1.25E+02 | 7.80E-01 | 2.58E+02 | Protonated | C00670 | sn-Glycero-3-phosphocholine | Cholines | 1.69E+05 | 2.15E+05 | 2.22E+05 | 2.66E+05 | 2.59E+05 | 2.57E+05 | 2.30E+05 | 1.79E+05 | 2.05E+05 | 1.34E+05 | 2.98E+05 | 2.16E+05 | 1.72E+05 | 2.00E+05 | 1.86E+05 |
| pma6281 | 1.45E+02 | 7.40E+01 | 7.50E-01 | 1.46E+02 | [M-H]- | - | Methylglutaric acid | Organic acids | 1.58E+05 | 1.80E+05 | 1.45E+05 | 1.49E+05 | 1.60E+05 | 1.32E+05 | 1.23E+05 | 1.38E+05 | 1.31E+05 | 4.24E+04 | 1.34E+05 | 8.82E+04 | 3.96E+04 | 4.44E+04 | 4.20E+04 |
| pma6298 | 9.60E+01 | 8.02E+01 | 1.16E+00 | 9.50E+01 | [M+H]+ | - | 3-Hydroxypyridine | Others | 2.62E+04 | 3.25E+04 | 3.37E+04 | 2.80E+04 | 2.07E+04 | 2.03E+04 | 2.16E+04 | 1.93E+04 | 2.05E+04 | 2.95E+04 | 2.00E+04 | 2.48E+04 | 4.18E+04 | 1.91E+04 | 3.05E+04 |
| pma6455 | 2.29E+02 | 1.57E+02 | 8.60E-01 | 2.30E+02 | [M-H]- | C00117 | Ribulose-5-phosphate | Carbohydrates | 2.04E+05 | 1.98E+05 | 2.00E+05 | 1.78E+05 | 2.09E+05 | 1.75E+05 | 1.23E+05 | 1.03E+05 | 1.13E+05 | 8.91E+04 | 1.86E+05 | 1.38E+05 | 1.42E+05 | 1.24E+05 | 1.33E+05 |
| pma6460 | 3.37E+02 | 2.76E+02 | 3.26E+00 | 3.38E+02 | [M-H]- | - | 5-O-p-Coumaroylquinic acid | Quinate and its derivatives | 1.99E+04 | 1.77E+04 | 1.90E+04 | 1.76E+04 | 2.06E+04 | 1.62E+04 | 1.38E+04 | 1.67E+04 | 1.53E+04 | 1.18E+04 | 1.14E+04 | 1.16E+04 | 6.84E+03 | 1.72E+04 | 1.20E+04 |
| pma6499 | 5.09E+02 | 3.48E+02 | 3.41E+00 | 5.08E+02 | [M+H]+ | - | Limocitrin O-hexoside | Flavone | 2.31E+05 | 2.70E+05 | 2.47E+05 | 2.59E+05 | 2.62E+05 | 2.52E+05 | 4.75E+05 | 3.88E+05 | 4.32E+05 | 3.61E+05 | 3.49E+05 | 3.55E+05 | 3.05E+05 | 2.73E+05 | 2.89E+05 |
| pma6515 | 7.55E+02 | 3.32E+02 | 4.73E+00 | 7.54E+02 | [M+H]+ | - | C-hexosyl-chrysin O-feruloylhexoside | Flavone C-glycosides | 1.78E+05 | 1.79E+05 | 1.67E+05 | 1.52E+05 | 1.41E+05 | 1.18E+05 | 2.60E+05 | 2.72E+05 | 2.66E+05 | 3.18E+05 | 2.57E+05 | 2.88E+05 | 2.71E+05 | 3.18E+05 | 2.95E+05 |
| pma6518 | 7.71E+02 | 1.78E+02 | 4.25E+00 | 7.70E+02 | [M+H]+ | - | C-pentosyl-chrysoeriol 7-O-feruloylhexoside | Flavone C-glycosides | 8.82E+04 | 6.04E+04 | 4.99E+04 | 3.73E+04 | 5.37E+04 | 2.77E+04 | 9.00E+00 | 9.68E+03 | 4.84E+03 | 1.09E+04 | 1.74E+04 | 1.42E+04 | 2.81E+04 | 9.55E+03 | 1.88E+04 |
| pma6561 | 3.41E+02 | 1.79E+02 | 2.86E+00 | 3.42E+02 | [M-H]- | - | Caffeic acid O-glucoside | Hydroxycinnamoyl derivatives | 1.02E+07 | 1.06E+07 | 1.01E+07 | 1.02E+07 | 1.01E+07 | 1.00E+07 | 1.22E+07 | 1.20E+07 | 1.21E+07 | 1.22E+07 | 1.09E+07 | 1.16E+07 | 8.74E+06 | 8.63E+06 | 8.69E+06 |
| pma6599 | 2.07E+02 | 1.47E+02 | 3.31E+00 | 2.06E+02 | [M+H]+ | - | 6-Hydroxymethylherniarin | Hydroxycinnamoyl derivatives | 9.87E+05 | 9.96E+05 | 1.03E+06 | 1.04E+06 | 9.81E+05 | 1.02E+06 | 1.20E+06 | 1.21E+06 | 1.21E+06 | 1.09E+06 | 1.15E+06 | 1.12E+06 | 8.40E+05 | 1.06E+06 | 9.50E+05 |
| pma6625 | 4.05E+02 | 2.42E+02 | 4.63E+00 | 4.06E+02 | [M-H]- | - | "E-3,4,5'-Trihydroxy-3'-glucopyranosylstilbene" | Others | 7.05E+04 | 6.96E+04 | 6.79E+04 | 6.88E+04 | 6.76E+04 | 7.11E+04 | 5.25E+04 | 4.92E+04 | 5.09E+04 | 6.20E+04 | 4.46E+04 | 5.33E+04 | 3.95E+04 | 4.27E+04 | 4.11E+04 |
| pma6638 | 4.77E+02 | 3.28E+02 | 5.30E+00 | 4.76E+02 | [M+H]+ | - | O-methylChrysoeriol 7-O-hexoside | Flavone | 5.69E+04 | 5.34E+04 | 5.26E+04 | 5.16E+04 | 6.18E+04 | 5.88E+04 | 4.94E+04 | 4.34E+04 | 4.64E+04 | 3.27E+04 | 3.98E+04 | 3.63E+04 | 8.40E+04 | 7.00E+04 | 7.70E+04 |
| pma6639 | 4.79E+02 | 3.18E+02 | 4.17E+00 | 4.78E+02 | [M+H]+ | - | Isorhamnetin O-hexoside | Flavonol | 4.88E+06 | 5.51E+06 | 5.78E+06 | 5.57E+06 | 4.70E+06 | 5.29E+06 | 4.22E+06 | 4.21E+06 | 4.22E+06 | 5.81E+06 | 3.29E+06 | 4.55E+06 | 4.58E+06 | 4.49E+06 | 4.54E+06 |
| pmb0037 | 1.66E+02 | 1.21E+02 | 1.53E+00 | 1.65E+02 | [M+H]+ | C06199 | Hordenine | Alkaloids | 2.30E+05 | 2.66E+05 | 2.35E+05 | 2.46E+05 | 2.26E+05 | 2.57E+05 | 2.19E+05 | 2.07E+05 | 2.13E+05 | 1.73E+05 | 1.97E+05 | 1.85E+05 | 1.98E+05 | 2.22E+05 | 2.10E+05 |
| pmb0064 | 3.74E+02 | 1.99E+02 | 2.05E+00 | 3.73E+02 | [M+H]+ | C04831 | DIMBOA glucoside | Others | 2.67E+06 | 3.36E+06 | 3.40E+06 | 3.74E+06 | 3.41E+06 | 3.55E+06 | 1.51E+06 | 1.77E+06 | 1.64E+06 | 1.81E+06 | 1.92E+06 | 1.87E+06 | 1.53E+06 | 2.08E+06 | 1.81E+06 |
| pmb0069 | 1.22E+02 | 1.05E+02 | 2.26E+00 | 1.21E+02 | [M+H]+ | C09815 | Benzamide | Others | 2.92E+06 | 3.83E+06 | 3.73E+06 | 3.61E+06 | 3.84E+06 | 3.80E+06 | 4.81E+06 | 4.79E+06 | 4.80E+06 | 4.59E+06 | 5.11E+06 | 4.85E+06 | 4.47E+06 | 4.74E+06 | 4.61E+06 |
| pmb0130 | 2.03E+02 | 1.33E+02 | 4.72E+00 | 2.02E+02 | [M+H]+ | - | N-Acetyl tryptamine | Phenolamides | 7.67E+04 | 7.71E+04 | 7.50E+04 | 7.22E+04 | 6.48E+04 | 6.59E+04 | 1.45E+04 | 1.37E+04 | 1.41E+04 | 3.04E+04 | 1.01E+04 | 2.03E+04 | 1.18E+04 | 2.92E+04 | 2.05E+04 |
| pmb0142 | 1.65E+02 | 9.55E+01 | 6.04E+00 | 1.64E+02 | [M+H]+ | C10945 | Caffeic aldehyde | Hydroxycinnamoyl derivatives | 2.23E+05 | 2.18E+05 | 2.28E+05 | 2.22E+05 | 2.07E+05 | 2.08E+05 | 1.17E+05 | 1.26E+05 | 1.22E+05 | 2.32E+05 | 1.19E+05 | 1.76E+05 | 2.43E+05 | 2.24E+05 | 2.34E+05 |
| pmb0159 | 6.96E+02 | 6.96E+02 | 7.60E+00 | 6.95E+02 | [M+H]+ | - | DGMG (18:2) isomer2 | Lipids_Glycerolipids | 5.29E+04 | 4.68E+04 | 4.36E+04 | 4.07E+04 | 5.01E+04 | 4.31E+04 | 3.31E+04 | 4.46E+04 | 3.89E+04 | 2.80E+04 | 2.22E+04 | 2.51E+04 | 3.57E+04 | 4.83E+05 | 2.59E+05 |
| pmb0160 | 3.53E+02 | 2.62E+02 | 7.60E+00 | 3.52E+02 | [M+H]+ | - | MAG (18:3) isomer5 | Lipids_Glycerolipids | 1.54E+06 | 1.48E+06 | 1.42E+06 | 1.38E+06 | 1.32E+06 | 1.30E+06 | 1.15E+06 | 1.11E+06 | 1.13E+06 | 7.80E+05 | 8.08E+05 | 7.94E+05 | 1.08E+06 | 1.47E+07 | 7.89E+06 |
| pmb0161 | 6.96E+02 | 3.38E+02 | 8.12E+00 | 6.95E+02 | [M+H]+ | - | DGMG (18:2) isomer1 | Lipids_Glycerolipids | 7.02E+05 | 7.97E+05 | 8.05E+05 | 8.09E+05 | 6.69E+05 | 7.19E+05 | 5.10E+05 | 5.41E+05 | 5.26E+05 | 3.95E+05 | 4.45E+05 | 4.20E+05 | 5.31E+05 | 6.89E+06 | 3.71E+06 |
| pmb0163 | 6.96E+02 | 6.96E+02 | 8.11E+00 | 6.95E+02 | [M+H]+ | - | DGMG (18:2) isomer3 | Lipids_Glycerolipids | 7.82E+05 | 8.00E+05 | 7.88E+05 | 7.84E+05 | 6.81E+05 | 7.40E+05 | 5.15E+05 | 5.02E+05 | 5.09E+05 | 4.07E+05 | 4.91E+05 | 4.49E+05 | 5.22E+05 | 7.18E+06 | 3.85E+06 |
| pmb0164 | 3.55E+02 | 2.64E+02 | 8.46E+00 | 3.54E+02 | [M+H]+ | - | MAG (18:2) isomer1 | Lipids_Glycerolipids | 9.44E+04 | 8.98E+04 | 1.03E+05 | 1.16E+05 | 1.02E+05 | 1.10E+05 | 1.68E+05 | 1.78E+05 | 1.73E+05 | 2.27E+05 | 1.55E+05 | 1.91E+05 | 1.52E+05 | 3.59E+05 | 2.56E+05 |
| pmb0165 | 4.94E+02 | 4.77E+02 | 8.55E+00 | 4.93E+02 | [M+H]+ | - | LysoPC 16:1 | Lipids_Glycerophospholipids | 1.00E+05 | 1.54E+05 | 1.66E+05 | 1.64E+05 | 1.74E+05 | 1.61E+05 | 1.00E+05 | 1.01E+05 | 1.01E+05 | 1.75E+05 | 1.08E+05 | 1.42E+05 | 2.36E+05 | 6.64E+05 | 4.50E+05 |
| pmb0197 | 2.98E+02 | 1.67E+02 | 1.16E+00 | 2.97E+02 | [M+H]+ | - | N2-methylguanosine | Nucleotide and its derivates | 1.39E+06 | 1.34E+06 | 1.35E+06 | 1.30E+06 | 1.22E+06 | 1.24E+06 | 7.38E+05 | 7.51E+05 | 7.45E+05 | 6.36E+05 | 6.91E+05 | 6.64E+05 | 7.65E+05 | 7.44E+05 | 7.55E+05 |
| pmb0202 | 1.53E+02 | 1.36E+02 | 1.18E+00 | 1.52E+02 | [M+H]+ | C00385 | Xanthine | Nucleotide and its derivates | 5.92E+05 | 8.59E+05 | 6.94E+05 | 7.60E+05 | 8.78E+05 | 6.99E+05 | 1.11E+06 | 8.97E+05 | 1.00E+06 | 5.27E+05 | 1.28E+06 | 9.04E+05 | 3.82E+05 | 5.83E+05 | 4.83E+05 |
| pmb0242 | 3.10E+02 | 2.52E+02 | 3.08E+00 | 3.10E+02 | Protonated | C00933 | Sinapoylcholine | Cholines | 3.63E+05 | 3.69E+05 | 4.04E+05 | 3.77E+05 | 2.98E+05 | 2.73E+05 | 1.30E+05 | 1.11E+05 | 1.21E+05 | 1.06E+05 | 1.33E+05 | 1.20E+05 | 9.73E+04 | 9.39E+04 | 9.56E+04 |
| pmb0247 | 1.37E+02 | 9.43E+01 | 3.14E+00 | 1.36E+02 | [M+H]+ | C00568 | p-Aminobenzoate | Benzoic acid derivatives | 7.35E+05 | 7.44E+05 | 7.74E+05 | 6.33E+05 | 6.35E+05 | 7.02E+05 | 1.02E+06 | 1.01E+06 | 1.02E+06 | 8.97E+05 | 8.24E+05 | 8.61E+05 | 6.82E+05 | 6.84E+05 | 6.83E+05 |
| pmb0277 | 3.31E+02 | 3.16E+02 | 5.74E+00 | 3.30E+02 | [M+H]+ | - | Tricin | Flavone | 2.38E+04 | 2.83E+04 | 2.85E+04 | 2.47E+04 | 2.50E+04 | 2.60E+04 | 2.67E+04 | 3.55E+04 | 3.11E+04 | 5.34E+04 | 5.30E+04 | 5.32E+04 | 2.73E+04 | 2.07E+04 | 2.40E+04 |
| pmb0287 | 3.51E+02 | 2.78E+02 | 7.47E+00 | 3.50E+02 | [M+H]+ | - | MAG (18:4) isomer2 | Lipids_Glycerolipids | 4.26E+04 | 3.81E+04 | 3.85E+04 | 3.54E+04 | 3.44E+04 | 3.35E+04 | 3.97E+04 | 3.91E+04 | 3.94E+04 | 1.89E+04 | 2.54E+04 | 2.22E+04 | 1.28E+04 | 7.09E+04 | 4.19E+04 |
| pmb0292 | 3.87E+02 | 3.32E+02 | 7.65E+00 | 3.86E+02 | [M+H]+ | C00187 | Cholesterol | Others | 9.46E+05 | 8.99E+05 | 9.06E+05 | 8.71E+05 | 8.41E+05 | 8.82E+05 | 7.25E+05 | 9.68E+05 | 8.47E+05 | 5.76E+05 | 4.01E+05 | 4.89E+05 | 3.93E+05 | 4.90E+05 | 4.42E+05 |
| pmb0296 | 3.57E+02 | 3.40E+02 | 8.97E+00 | 3.56E+02 | [M+H]+ | - | MAG (18:1) isomer2 | Lipids_Glycerolipids | 8.33E+05 | 7.65E+05 | 7.52E+05 | 7.18E+05 | 6.55E+05 | 7.48E+05 | 9.94E+05 | 7.43E+05 | 8.69E+05 | 1.24E+06 | 9.73E+05 | 1.11E+06 | 8.96E+05 | 1.95E+06 | 1.42E+06 |
| pmb0302 | 1.26E+02 | 9.73E+01 | 3.69E+00 | 1.25E+02 | [M+H]+ | C03557 | 2-Aminoethylphosphonate | Others | 3.11E+04 | 4.25E+04 | 3.83E+04 | 3.07E+04 | 2.40E+04 | 2.69E+04 | 2.90E+04 | 2.82E+04 | 2.86E+04 | 4.69E+04 | 1.59E+04 | 3.14E+04 | 2.03E+04 | 2.83E+04 | 2.43E+04 |
| pmb0322 | 6.53E+02 | 3.46E+02 | 4.76E+00 | 6.52E+02 | [M+H]+ | - | "3’,4’,5’-Tricetin O-rutinoside" | Flavone | 1.22E+04 | 1.57E+04 | 1.70E+04 | 9.22E+03 | 1.15E+04 | 1.09E+04 | 6.66E+03 | 7.25E+03 | 6.96E+03 | 9.00E+00 | 1.18E+04 | 5.90E+03 | 9.00E+00 | 1.07E+04 | 5.35E+03 |
| pmb0323 | 2.51E+02 | 2.34E+02 | 1.97E+00 | 2.50E+02 | [M+H]+ | C03002 | N-Caffeoyl putrescine | Phenolamides | 8.70E+05 | 9.62E+05 | 1.17E+06 | 1.21E+06 | 9.10E+05 | 1.33E+06 | 4.90E+05 | 5.70E+05 | 5.30E+05 | 4.43E+05 | 3.15E+05 | 3.79E+05 | 2.93E+05 | 4.16E+05 | 3.55E+05 |
| pmb0374 | 1.36E+02 | 1.19E+02 | 1.82E+00 | 1.35E+02 | [M+H]+ | - | Aminopurine | Others | 1.09E+07 | 1.24E+07 | 1.29E+07 | 1.09E+07 | 1.14E+07 | 1.30E+07 | 8.54E+06 | 8.01E+06 | 8.28E+06 | 1.39E+07 | 1.10E+07 | 1.25E+07 | 1.05E+07 | 8.69E+06 | 9.60E+06 |
| pmb0382 | 3.39E+02 | 1.78E+02 | 3.88E+00 | 3.38E+02 | [M+H]+ | - | O-Feruloyl 4-hydroxylcoumarin | Coumarins | 1.43E+07 | 1.84E+07 | 2.03E+07 | 2.00E+07 | 1.72E+07 | 1.85E+07 | 2.41E+07 | 2.28E+07 | 2.35E+07 | 2.68E+07 | 1.48E+07 | 2.08E+07 | 2.33E+07 | 2.38E+07 | 2.36E+07 |
| pmb0423 | 1.95E+02 | 1.78E+02 | 3.22E+00 | 1.94E+02 | [M+H]+ | - | Hydroxy-methoxycinnamate | Hydroxycinnamoyl derivatives | 6.22E+06 | 6.36E+06 | 6.73E+06 | 6.90E+06 | 6.90E+06 | 6.49E+06 | 7.70E+06 | 7.52E+06 | 7.61E+06 | 6.07E+06 | 6.49E+06 | 6.28E+06 | 4.54E+06 | 4.90E+06 | 4.72E+06 |
| pmb0426 | 2.19E+02 | 1.90E+02 | 3.84E+00 | 2.18E+02 | [M+H]+ | C08309 | "5-Methoxy-N,N-dimethyltryptamine" | Tryptamine derivatives | 8.67E+04 | 7.75E+04 | 7.90E+04 | 6.94E+04 | 7.05E+04 | 5.98E+04 | 6.66E+04 | 1.40E+05 | 1.03E+05 | 9.08E+04 | 1.16E+05 | 1.03E+05 | 5.33E+04 | 6.58E+04 | 5.96E+04 |
| pmb0449 | 1.62E+02 | 9.83E+01 | 8.00E-01 | 1.61E+02 | [M+H]+ | C00956 | 2-Aminoadipic acid (L-Homoglutamic acid) | Amino acids | 2.99E+05 | 4.62E+05 | 4.27E+05 | 4.86E+05 | 4.75E+05 | 5.17E+05 | 7.01E+05 | 4.83E+05 | 5.92E+05 | 5.01E+05 | 4.67E+05 | 4.84E+05 | 3.54E+05 | 4.91E+05 | 4.23E+05 |
| pmb0464 | 4.58E+02 | 1.16E+02 | 8.10E-01 | 4.57E+02 | [M+H]+ | - | Aspartic acid di-O-glucoside | Amino acid derivatives | 3.28E+07 | 2.63E+07 | 3.38E+07 | 2.14E+07 | 2.19E+07 | 2.43E+07 | 2.20E+07 | 1.56E+07 | 1.88E+07 | 1.86E+07 | 1.86E+07 | 1.86E+07 | 2.36E+07 | 2.16E+07 | 2.26E+07 |
| pmb0468 | 1.78E+02 | 1.62E+02 | 1.65E+00 | 1.77E+02 | [M+H]+ | C03145 | N-formylmethionine | Amino acid derivatives | 9.34E+05 | 1.25E+06 | 1.01E+06 | 9.78E+05 | 8.32E+05 | 8.95E+05 | 1.42E+06 | 1.32E+06 | 1.37E+06 | 7.65E+05 | 6.28E+05 | 6.97E+05 | 4.59E+05 | 7.61E+05 | 6.10E+05 |
| pmb0475 | 6.71E+02 | 6.71E+02 | 2.85E+00 | 6.70E+02 | [M+H]+ | - | Gallic acid O-feruloyl-O-hexosyl-O-hexoside | Hydroxycinnamoyl derivatives | 2.29E+05 | 2.10E+05 | 1.94E+05 | 2.10E+05 | 2.74E+05 | 1.28E+05 | 3.40E+05 | 2.75E+05 | 3.08E+05 | 4.48E+05 | 2.92E+05 | 3.70E+05 | 2.96E+05 | 3.49E+05 | 3.23E+05 |
| pmb0478 | 4.55E+02 | 4.37E+02 | 1.92E+00 | 4.54E+02 | [M+H]+ | - | Coumarin O-rutinoside | Hydroxycinnamoyl derivatives | 8.76E+04 | 1.01E+05 | 1.01E+05 | 1.29E+05 | 1.37E+05 | 1.10E+05 | 9.89E+04 | 6.47E+04 | 8.18E+04 | 4.40E+04 | 7.28E+04 | 5.84E+04 | 5.99E+04 | 7.54E+04 | 6.77E+04 |
| pmb0484 | 1.04E+02 | 6.02E+01 | 7.60E-01 | 1.03E+02 | [M+H]+ | C00114 | Choline | Cholines | 9.76E+05 | 1.43E+06 | 1.62E+06 | 1.62E+06 | 1.49E+06 | 1.63E+06 | 1.86E+06 | 1.92E+06 | 1.89E+06 | 1.67E+06 | 1.44E+06 | 1.56E+06 | 1.59E+06 | 1.49E+06 | 1.54E+06 |
| pmb0489 | 3.97E+02 | 1.47E+02 | 1.73E+00 | 3.96E+02 | [M+H]+ | - | N-hexosyl-p-coumaroyl putrescine | Phenolamides | 1.48E+06 | 1.61E+06 | 1.49E+06 | 1.71E+03 | 1.61E+06 | 1.47E+06 | 8.43E+05 | 7.79E+05 | 8.11E+05 | 1.16E+06 | 9.12E+05 | 1.04E+06 | 1.26E+06 | 1.76E+06 | 1.51E+06 |
| pmb0490 | 2.35E+02 | 1.48E+02 | 2.29E+00 | 2.34E+02 | [M+H]+ | C18326 | N-p-Coumaroyl putrescine | Phenolamides | 5.53E+05 | 6.27E+05 | 6.01E+05 | 6.54E+05 | 6.63E+05 | 5.69E+05 | 9.63E+05 | 9.45E+05 | 9.54E+05 | 7.35E+05 | 6.23E+05 | 6.79E+05 | 4.90E+05 | 7.71E+05 | 6.31E+05 |
| pmb0492 | 5.84E+02 | 3.26E+02 | 2.42E+00 | 5.83E+02 | [M+H]+ | - | "N',N"",N""'-p-coumaroyl-cinnamoyl-caffeoyl spermidine" | Phenolamides | 6.88E+04 | 7.64E+04 | 7.19E+04 | 7.72E+04 | 8.71E+04 | 7.36E+04 | 1.07E+05 | 9.81E+04 | 1.03E+05 | 3.48E+04 | 4.10E+04 | 3.79E+04 | 8.07E+04 | 6.60E+04 | 7.34E+04 |
| pmb0493 | 2.77E+02 | 2.61E+02 | 2.58E+00 | 2.76E+02 | [M+H]+ | - | N'-p-Coumaroyl agmatine | Phenolamides | 9.97E+04 | 1.01E+05 | 9.42E+04 | 1.00E+05 | 7.08E+04 | 7.14E+04 | 6.54E+05 | 6.72E+05 | 6.63E+05 | 6.12E+04 | 1.31E+05 | 9.61E+04 | 3.54E+04 | 3.46E+04 | 3.50E+04 |
| pmb0494 | 2.95E+02 | 2.08E+02 | 2.77E+00 | 2.94E+02 | [M+H]+ | - | N-Sinapoyl putrescine | Phenolamides | 3.99E+05 | 4.04E+05 | 3.70E+05 | 3.84E+05 | 3.83E+05 | 3.42E+05 | 4.87E+05 | 4.73E+05 | 4.80E+05 | 9.02E+05 | 7.51E+05 | 8.27E+05 | 8.08E+05 | 9.19E+05 | 8.64E+05 |
| pmb0496 | 3.07E+02 | 1.78E+02 | 2.78E+00 | 3.06E+02 | [M+H]+ | C18325 | N-Feruloyl agmatine | Phenolamides | 5.36E+06 | 5.55E+06 | 5.37E+06 | 5.62E+06 | 5.26E+06 | 5.31E+06 | 3.35E+07 | 3.88E+07 | 3.62E+07 | 7.63E+06 | 1.11E+07 | 9.37E+06 | 5.62E+06 | 8.87E+06 | 7.25E+06 |
| pmb0501 | 1.31E+02 | 7.21E+01 | 7.50E-01 | 1.30E+02 | [M+H]+ | C00179 | Agmatine | Phenolamides | 1.51E+05 | 2.10E+05 | 2.58E+05 | 2.36E+05 | 1.69E+05 | 1.86E+05 | 2.87E+05 | 2.43E+05 | 2.65E+05 | 2.33E+05 | 1.43E+05 | 1.88E+05 | 1.90E+05 | 2.13E+05 | 2.02E+05 |
| pmb0503 | 4.39E+02 | 1.47E+02 | 2.11E+00 | 4.38E+02 | [M+H]+ | - | N-(4'-O-glycosyl)-p-coumaroyl agmatine | Phenolamides | 9.34E+04 | 1.24E+05 | 1.23E+05 | 1.11E+05 | 1.18E+05 | 9.68E+04 | 2.75E+05 | 3.48E+05 | 3.12E+05 | 4.97E+04 | 1.57E+05 | 1.03E+05 | 7.78E+04 | 7.74E+04 | 7.76E+04 |
| pmb0504 | 4.69E+02 | 3.08E+02 | 2.17E+00 | 4.68E+02 | [M+H]+ | - | N-(4'-O-glycosyl)-feruloyl agmatine | Phenolamides | 5.55E+05 | 5.45E+05 | 6.04E+05 | 5.63E+05 | 5.64E+05 | 5.12E+05 | 1.37E+06 | 1.55E+06 | 1.46E+06 | 4.62E+05 | 8.43E+05 | 6.53E+05 | 7.16E+05 | 1.25E+06 | 9.83E+05 |
| pmb0505 | 2.35E+02 | 1.47E+02 | 2.38E+00 | 2.34E+02 | [M+H]+ | - | N'-p-Coumaroyl putrescine | Phenolamides | 7.16E+05 | 7.84E+05 | 7.70E+05 | 7.27E+05 | 7.52E+05 | 6.62E+05 | 1.16E+06 | 1.27E+06 | 1.22E+06 | 8.62E+05 | 7.63E+05 | 8.13E+05 | 5.31E+05 | 9.02E+05 | 7.17E+05 |
| pmb0514 | 3.48E+02 | 1.36E+02 | 8.50E-01 | 3.47E+02 | [M+H]+ | C01367 | Adenosine 3'-monophosphate | Nucleotide and its derivates | 3.49E+06 | 3.77E+06 | 3.48E+06 | 3.60E+06 | 3.50E+06 | 3.34E+06 | 1.04E+06 | 1.40E+06 | 1.22E+06 | 1.28E+06 | 1.69E+06 | 1.49E+06 | 7.91E+05 | 9.12E+05 | 8.52E+05 |
| pmb0530 | 6.64E+02 | 5.24E+02 | 1.16E+00 | 6.63E+02 | [M+H]+ | C00003 | Nicotinic acid adenine dinucleotide | Nucleotide and its derivates | 6.81E+06 | 8.27E+06 | 8.72E+06 | 7.78E+06 | 8.07E+06 | 8.54E+06 | 3.45E+06 | 3.11E+06 | 3.28E+06 | 5.91E+06 | 4.41E+06 | 5.16E+06 | 6.15E+06 | 8.95E+06 | 7.55E+06 |
| pmb0532 | 3.49E+02 | 1.37E+02 | 1.16E+00 | 3.48E+02 | [M+H]+ | C00130 | Inosine 5'-monophosphate | Nucleotide and its derivates | 9.63E+05 | 1.29E+06 | 1.14E+06 | 1.10E+06 | 1.23E+06 | 9.83E+05 | 3.41E+05 | 2.96E+05 | 3.19E+05 | 4.06E+05 | 3.97E+05 | 4.02E+05 | 2.68E+05 | 2.24E+05 | 2.46E+05 |
| pmb0541 | 6.97E+02 | 6.97E+02 | 2.09E+00 | 6.97E+02 | Protonated | - | Cyanidin 3-O-glucosyl-malonylglucoside | Anthocyanins | 1.20E+05 | 1.15E+05 | 1.19E+05 | 5.66E+04 | 9.33E+04 | 1.07E+05 | 9.31E+04 | 8.39E+04 | 8.85E+04 | 1.48E+05 | 7.15E+04 | 1.10E+05 | 9.57E+04 | 1.14E+05 | 1.05E+05 |
| pmb0545 | 4.77E+02 | 3.16E+02 | 3.32E+00 | 4.77E+02 | Protonated | - | Rosinidin O-hexoside | Anthocyanins | 1.13E+07 | 1.03E+07 | 1.10E+07 | 1.00E+07 | 9.24E+06 | 1.04E+07 | 5.65E+06 | 4.72E+06 | 5.19E+06 | 2.74E+06 | 4.35E+06 | 3.55E+06 | 2.81E+07 | 1.73E+07 | 2.27E+07 |
| pmb0550 | 4.49E+02 | 2.87E+02 | 2.59E+00 | 4.49E+02 | Protonated | C08604 | Cyanidin 3-O-glucoside (Kuromanin) | Anthocyanins | 1.23E+05 | 1.38E+05 | 1.01E+05 | 9.58E+04 | 1.14E+05 | 1.40E+05 | 1.47E+05 | 1.31E+05 | 1.39E+05 | 1.55E+05 | 1.54E+05 | 1.55E+05 | 1.62E+05 | 1.09E+05 | 1.36E+05 |
| pmb0565 | 5.09E+02 | 3.48E+02 | 3.41E+00 | 5.08E+02 | [M+H]+ | - | Syringetin 3-O-hexoside | Flavonol | 3.00E+05 | 2.40E+05 | 2.83E+05 | 2.21E+05 | 2.38E+05 | 2.74E+05 | 4.33E+05 | 3.31E+05 | 3.82E+05 | 3.85E+05 | 2.90E+05 | 3.38E+05 | 2.11E+05 | 2.68E+05 | 2.40E+05 |
| pmb0566 | 5.81E+02 | 2.87E+02 | 3.74E+00 | 5.80E+02 | [M+H]+ | - | Luteolin O-hexosyl-O-pentoside | Flavone | 5.21E+04 | 7.13E+04 | 6.90E+04 | 5.38E+04 | 5.10E+04 | 6.19E+04 | 7.35E+04 | 8.44E+04 | 7.90E+04 | 2.59E+04 | 4.38E+04 | 3.49E+04 | 2.70E+04 | 2.86E+04 | 2.78E+04 |
| pmb0569 | 5.09E+02 | 3.48E+02 | 4.17E+00 | 5.08E+02 | [M+H]+ | - | Syringetin 5-O-hexoside | Flavone | 1.80E+05 | 1.80E+05 | 1.80E+05 | 1.45E+05 | 1.55E+05 | 1.62E+05 | 1.76E+05 | 1.95E+05 | 1.86E+05 | 2.54E+05 | 2.34E+05 | 2.44E+05 | 1.06E+05 | 1.26E+05 | 1.16E+05 |
| pmb0587 | 6.39E+02 | 6.40E+02 | 3.29E+00 | 6.38E+02 | [M+H]+ | - | Chrysoeriol O-glucuronic acid-O-hexoside | Flavone | 5.79E+05 | 5.86E+05 | 6.35E+05 | 5.67E+05 | 5.57E+05 | 4.51E+05 | 3.80E+05 | 3.41E+05 | 3.61E+05 | 1.53E+05 | 2.44E+05 | 1.99E+05 | 1.37E+06 | 1.03E+06 | 1.20E+06 |
| pmb0588 | 6.11E+02 | 4.49E+02 | 3.32E+00 | 6.10E+02 | [M+H]+ | - | "Luteolin 3',7-di-O-glucoside" | Flavone | 2.84E+05 | 2.52E+05 | 3.06E+05 | 3.10E+05 | 2.75E+05 | 2.42E+05 | 1.90E+05 | 1.75E+05 | 1.83E+05 | 2.51E+05 | 1.52E+05 | 2.02E+05 | 2.75E+05 | 1.85E+05 | 2.30E+05 |
| pmb0590 | 4.61E+02 | 2.57E+02 | 3.76E+00 | 4.60E+02 | [M+H]+ | - | Acacetin O-glucuronic acid | Flavone | 1.57E+04 | 2.64E+04 | 3.27E+04 | 2.91E+04 | 2.67E+04 | 2.63E+04 | 2.87E+04 | 2.77E+04 | 2.82E+04 | 1.18E+04 | 2.58E+04 | 1.88E+04 | 1.55E+04 | 3.40E+04 | 2.48E+04 |
| pmb0595 | 4.79E+02 | 3.18E+02 | 4.17E+00 | 4.78E+02 | [M+H]+ | - | Isorhamnetin 5-O-hexoside | Flavonol | 4.38E+06 | 5.60E+06 | 4.89E+06 | 5.40E+06 | 4.81E+06 | 4.77E+06 | 4.15E+06 | 4.45E+06 | 4.30E+06 | 5.32E+06 | 3.20E+06 | 4.26E+06 | 3.96E+06 | 3.94E+06 | 3.95E+06 |
| pmb0602 | 5.09E+02 | 2.84E+02 | 4.17E+00 | 5.08E+02 | [M+H]+ | - | Syringetin 7-O-hexoside | Flavone | 1.49E+05 | 1.25E+05 | 1.46E+05 | 1.22E+05 | 1.32E+05 | 1.57E+05 | 1.72E+05 | 2.12E+05 | 1.92E+05 | 2.24E+05 | 1.87E+05 | 2.06E+05 | 1.11E+05 | 1.22E+05 | 1.17E+05 |
| pmb0603 | 6.25E+02 | 3.32E+02 | 4.17E+00 | 6.24E+02 | [M+H]+ | - | Chrysoeriol O-hexosyl-O-hexoside | Flavone | 4.40E+05 | 4.11E+05 | 3.74E+05 | 3.36E+05 | 3.35E+05 | 2.75E+05 | 2.46E+05 | 2.30E+05 | 2.38E+05 | 3.97E+05 | 2.26E+05 | 3.12E+05 | 3.17E+05 | 2.72E+05 | 2.95E+05 |
| pmb0608 | 5.49E+02 | 3.02E+02 | 4.55E+00 | 5.48E+02 | [M+H]+ | - | Chrysoeriol O-malonylhexoside | Flavone | 3.19E+04 | 2.47E+04 | 3.76E+04 | 3.30E+04 | 3.16E+04 | 2.20E+04 | 2.17E+04 | 4.47E+04 | 3.32E+04 | 5.34E+04 | 3.50E+04 | 4.42E+04 | 4.61E+04 | 3.85E+04 | 4.23E+04 |
| pmb0615 | 7.89E+02 | 7.89E+02 | 2.58E+00 | 7.88E+02 | [M+H]+ | - | Hesperetin C-hexosyl-O-hexosyl-O-hexoside | Flavone C-glycosides | 8.46E+05 | 7.59E+05 | 6.60E+05 | 7.42E+05 | 7.49E+05 | 4.92E+05 | 3.16E+05 | 3.11E+05 | 3.14E+05 | 1.23E+06 | 5.52E+05 | 8.91E+05 | 9.88E+05 | 7.11E+05 | 8.50E+05 |
| pmb0618 | 6.27E+02 | 4.30E+02 | 2.78E+00 | 6.26E+02 | [M+H]+ | - | 8-C-hexosyl-hesperetin O-hexoside | Flavone C-glycosides | 7.08E+06 | 8.25E+06 | 8.02E+06 | 8.14E+06 | 7.40E+06 | 7.27E+06 | 7.69E+06 | 7.77E+06 | 7.73E+06 | 8.41E+06 | 8.05E+06 | 8.23E+06 | 6.76E+06 | 7.66E+06 | 7.21E+06 |
| pmb0619 | 7.75E+02 | 7.75E+02 | 2.67E+00 | 7.74E+02 | [M+H]+ | - | Eriodictiol 6-C-hexoside 8-C-hexoside-O-hexoside | Flavone C-glycosides | 7.08E+06 | 7.67E+06 | 7.76E+06 | 8.31E+06 | 7.61E+06 | 7.31E+06 | 8.25E+06 | 7.84E+06 | 8.05E+06 | 8.15E+06 | 6.73E+06 | 7.44E+06 | 6.97E+06 | 8.06E+06 | 7.52E+06 |
| pmb0624 | 6.11E+02 | 3.29E+02 | 3.09E+00 | 6.10E+02 | [M+H]+ | - | 6-C-hexosyl-luteolin O-hexoside | Flavone C-glycosides | 1.11E+05 | 1.12E+05 | 1.37E+05 | 1.14E+05 | 8.65E+04 | 9.84E+04 | 7.90E+04 | 6.40E+04 | 7.15E+04 | 7.81E+04 | 8.56E+04 | 8.19E+04 | 9.84E+04 | 1.02E+05 | 1.00E+05 |
| pmb0626 | 7.57E+02 | 7.57E+02 | 3.10E+00 | 7.56E+02 | [M+H]+ | - | 6-C-hexosyl-apigenin O-hexosyl-O-hexoside | Flavone C-glycosides | 1.24E+05 | 1.60E+05 | 1.89E+05 | 1.55E+05 | 1.40E+05 | 1.22E+05 | 1.06E+05 | 8.71E+04 | 9.66E+04 | 1.09E+05 | 8.97E+04 | 9.94E+04 | 1.20E+05 | 1.25E+05 | 1.23E+05 |
| pmb0636 | 5.81E+02 | 3.36E+02 | 3.18E+00 | 5.80E+02 | [M+H]+ | - | 6-C-hexosyl luteolin O-pentoside | Flavone C-glycosides | 6.34E+04 | 5.83E+04 | 5.80E+04 | 4.38E+04 | 4.25E+04 | 3.95E+04 | 2.98E+04 | 2.89E+04 | 2.94E+04 | 3.88E+04 | 3.19E+04 | 3.54E+04 | 1.65E+04 | 2.88E+04 | 2.27E+04 |
| pmb0639 | 7.57E+02 | 7.57E+02 | 3.13E+00 | 7.56E+02 | [M+H]+ | - | 8-C-hexosyl-apigenin O-hexosyl-O-hexoside | Flavone C-glycosides | 1.51E+06 | 1.49E+06 | 1.36E+06 | 1.49E+06 | 1.42E+06 | 1.35E+06 | 8.82E+05 | 8.66E+05 | 8.74E+05 | 1.26E+06 | 9.27E+05 | 1.09E+06 | 1.14E+06 | 1.15E+06 | 1.15E+06 |
| pmb0645 | 6.27E+02 | 4.47E+02 | 3.41E+00 | 6.26E+02 | [M+H]+ | - | 6-C-hexosyl-hesperetin O-hexoside | Flavone C-glycosides | 2.41E+06 | 2.30E+06 | 2.37E+06 | 2.22E+06 | 2.06E+06 | 2.21E+06 | 9.94E+05 | 1.10E+06 | 1.05E+06 | 2.59E+06 | 1.50E+06 | 2.05E+06 | 1.94E+06 | 1.52E+06 | 1.73E+06 |
| pmb0653 | 5.95E+02 | 4.15E+02 | 3.68E+00 | 5.94E+02 | [M+H]+ | - | "di-C,C-hexosyl-apigenin" | Flavone C-glycosides | 4.07E+04 | 5.95E+04 | 3.79E+04 | 4.53E+04 | 4.49E+04 | 5.06E+04 | 5.81E+04 | 8.24E+04 | 7.03E+04 | 3.01E+04 | 2.17E+04 | 2.59E+04 | 3.68E+04 | 4.76E+04 | 4.22E+04 |
| pmb0661 | 6.09E+02 | 4.63E+02 | 3.87E+00 | 6.08E+02 | [M+H]+ | - | Chrysoeriol C-hexosyl-O-rhamnoside | Flavone C-glycosides | 2.65E+05 | 2.59E+05 | 2.92E+05 | 2.58E+05 | 2.19E+05 | 2.13E+05 | 2.29E+05 | 1.59E+05 | 1.94E+05 | 3.04E+05 | 1.61E+05 | 2.33E+05 | 2.81E+05 | 2.98E+05 | 2.90E+05 |
| pmb0662 | 7.87E+02 | 7.33E+02 | 3.64E+00 | 7.86E+02 | [M+H]+ | - | C-hexosyl-luteolin O-feruloylhexoside | Flavone C-glycosides | 3.95E+05 | 4.21E+05 | 4.14E+05 | 3.35E+05 | 3.32E+05 | 4.34E+05 | 3.50E+05 | 3.18E+05 | 3.34E+05 | 1.13E+05 | 1.68E+05 | 1.41E+05 | 2.91E+05 | 4.60E+05 | 3.76E+05 |
| pmb0663 | 6.11E+02 | 3.00E+02 | 3.75E+00 | 6.10E+02 | [M+H]+ | - | 8-C-hexosyl-luteolin O-hexoside | Flavone C-glycosides | 1.16E+05 | 1.52E+05 | 1.14E+05 | 1.54E+05 | 7.95E+04 | 1.10E+05 | 8.04E+04 | 9.16E+04 | 8.60E+04 | 7.97E+04 | 8.62E+04 | 8.30E+04 | 8.19E+04 | 6.85E+04 | 7.52E+04 |
| pmb0665 | 6.11E+02 | 3.00E+02 | 3.78E+00 | 6.10E+02 | [M+H]+ | - | Luteolin 8-C-hexosyl-O-hexoside | Flavone C-glycosides | 2.71E+07 | 2.94E+07 | 2.57E+07 | 2.49E+07 | 2.31E+07 | 2.38E+07 | 2.12E+07 | 2.25E+07 | 2.19E+07 | 2.44E+07 | 1.74E+07 | 2.09E+07 | 2.16E+07 | 2.22E+07 | 2.19E+07 |
| pmb0677 | 8.31E+02 | 8.31E+02 | 3.84E+00 | 8.30E+02 | [M+H]+ | - | C-hexosyl-chrysoeriol O-sinapoylhexoside | Flavone C-glycosides | 2.27E+04 | 3.23E+04 | 3.32E+04 | 2.72E+04 | 2.70E+04 | 2.73E+04 | 2.52E+04 | 2.90E+04 | 2.71E+04 | 3.44E+04 | 3.56E+04 | 3.50E+04 | 1.59E+04 | 3.15E+04 | 2.37E+04 |
| pmb0678 | 7.71E+02 | 7.53E+02 | 4.26E+00 | 7.70E+02 | [M+H]+ | - | 8-C-hexosyl-apigenin O-feruloylhexoside | Flavone C-glycosides | 7.11E+04 | 6.99E+04 | 4.13E+04 | 6.02E+04 | 5.23E+04 | 3.74E+04 | 3.19E+04 | 2.21E+04 | 2.70E+04 | 1.40E+04 | 1.57E+04 | 1.49E+04 | 2.60E+04 | 2.82E+04 | 2.71E+04 |
| pmb0680 | 7.41E+02 | 7.41E+02 | 3.97E+00 | 7.40E+02 | [M+H]+ | - | C-hexosyl-apigenin O-p-coumaroylhexoside | Flavone C-glycosides | 3.57E+04 | 4.33E+04 | 5.39E+04 | 4.61E+04 | 2.37E+04 | 2.76E+04 | 9.00E+00 | 7.87E+03 | 3.94E+03 | 8.60E+03 | 8.36E+03 | 8.48E+03 | 2.08E+04 | 2.77E+03 | 1.18E+04 |
| pmb0691 | 7.57E+02 | 7.57E+02 | 3.09E+00 | 7.56E+02 | [M+H]+ | - | Luteolin C-hexosyl-O-rhamnoside O-hexoside | Flavone C-glycosides | 1.94E+05 | 1.57E+05 | 1.80E+05 | 1.88E+05 | 1.72E+05 | 1.61E+05 | 1.08E+05 | 8.33E+04 | 9.57E+04 | 1.65E+05 | 7.88E+04 | 1.22E+05 | 1.16E+05 | 1.48E+05 | 1.32E+05 |
| pmb0711 | 6.11E+02 | 3.04E+02 | 3.70E+00 | 6.10E+02 | [M+H]+ | - | Quercetin 7-O-rutinoside | Flavonol | 8.26E+06 | 9.48E+06 | 1.05E+07 | 9.53E+06 | 8.80E+06 | 9.03E+06 | 7.96E+06 | 7.82E+06 | 7.89E+06 | 8.88E+06 | 6.04E+06 | 7.46E+06 | 8.53E+06 | 8.30E+06 | 8.42E+06 |
| pmb0712 | 6.55E+02 | 3.32E+02 | 3.50E+00 | 6.54E+02 | [M+H]+ | - | Tricin 5-O-hexosyl-O-hexoside | Flavone | 1.25E+05 | 2.07E+05 | 1.89E+05 | 1.58E+05 | 1.14E+05 | 1.48E+05 | 6.69E+04 | 7.91E+04 | 7.30E+04 | 7.47E+04 | 8.67E+04 | 8.07E+04 | 1.94E+05 | 1.76E+05 | 1.85E+05 |
| pmb0713 | 6.55E+02 | 3.32E+02 | 3.37E+00 | 6.54E+02 | [M+H]+ | - | Tricin 7-O-hexosyl-O-hexoside | Flavone | 8.48E+04 | 6.42E+04 | 7.59E+04 | 9.12E+04 | 1.15E+05 | 3.65E+04 | 2.91E+04 | 8.33E+04 | 5.62E+04 | 2.22E+04 | 4.06E+04 | 3.14E+04 | 1.03E+05 | 9.04E+04 | 9.67E+04 |
| pmb0720 | 5.79E+02 | 3.32E+02 | 4.56E+00 | 5.78E+02 | [M+H]+ | - | Tricin O-malonylhexoside | Flavone | 2.47E+04 | 2.74E+04 | 2.80E+04 | 2.50E+04 | 1.58E+04 | 1.69E+04 | 4.66E+04 | 4.07E+04 | 4.37E+04 | 2.64E+04 | 1.67E+04 | 2.16E+04 | 4.05E+04 | 1.65E+04 | 2.85E+04 |
| pmb0749 | 3.69E+02 | 1.78E+02 | 2.12E+00 | 3.68E+02 | [M+H]+ | - | O-Feruloyl quinic acid | Quinate and its derivatives | 7.84E+04 | 7.09E+04 | 9.13E+04 | 8.08E+04 | 3.89E+04 | 4.11E+04 | 1.50E+05 | 1.56E+05 | 1.53E+05 | 1.14E+05 | 8.12E+04 | 9.76E+04 | 6.02E+04 | 9.88E+04 | 7.95E+04 |
| pmb0750 | 3.55E+02 | 1.45E+02 | 2.35E+00 | 3.54E+02 | [M+H]+ | C17147 | Neochlorogenic acid (5-O-Caffeoylquinic acid) | Quinate and its derivatives | 4.84E+06 | 5.95E+06 | 6.15E+06 | 1.23E+07 | 5.66E+06 | 4.45E+06 | 7.85E+06 | 6.41E+06 | 7.13E+06 | 7.58E+06 | 7.76E+06 | 7.67E+06 | 7.90E+06 | 5.96E+06 | 6.93E+06 |
| pmb0751 | 3.21E+02 | 1.48E+02 | 3.81E+00 | 3.20E+02 | [M+H]+ | - | 5-O-p-Coumaroyl shikimic acid | Quinate and its derivatives | 1.64E+05 | 1.64E+05 | 1.54E+05 | 1.78E+05 | 1.56E+05 | 1.65E+05 | 6.54E+04 | 6.44E+04 | 6.49E+04 | 1.29E+05 | 7.91E+04 | 1.04E+05 | 1.35E+05 | 1.19E+05 | 1.27E+05 |
| pmb0752 | 3.69E+02 | 1.78E+02 | 3.01E+00 | 3.68E+02 | [M+H]+ | C02572 | 3-O-Feruloyl quinic acid | Quinate and its derivatives | 8.11E+04 | 1.24E+05 | 1.20E+05 | 1.05E+05 | 9.62E+04 | 1.02E+05 | 1.98E+05 | 1.70E+05 | 1.84E+05 | 1.52E+05 | 1.38E+05 | 1.45E+05 | 1.27E+05 | 1.37E+05 | 1.32E+05 |
| pmb0764 | 1.44E+02 | 1.43E+02 | 2.60E+00 | 1.43E+02 | [M+H]+ | C04294 | 4-Methyl-5-thiazoleethanol | Others | 4.37E+04 | 5.33E+04 | 4.40E+04 | 4.36E+04 | 4.41E+04 | 5.27E+04 | 5.94E+04 | 4.80E+04 | 5.37E+04 | 3.89E+04 | 3.88E+04 | 3.89E+04 | 3.54E+04 | 3.57E+04 | 3.56E+04 |
| pmb0766 | 3.90E+02 | 2.85E+02 | 7.20E+00 | 3.89E+02 | [M+H]+ | - | Azoxystrobin acid | Others | 2.48E+06 | 2.42E+06 | 2.52E+06 | 2.35E+06 | 2.17E+06 | 2.27E+06 | 2.28E+06 | 1.85E+06 | 2.07E+06 | 1.47E+06 | 1.54E+06 | 1.51E+06 | 1.93E+06 | 2.07E+06 | 2.00E+06 |
| pmb0767 | 3.02E+02 | 2.85E+02 | 7.25E+00 | 3.01E+02 | [M+H]+ | - | D-erythro-Dihydrosphingosine | Others | 1.90E+04 | 2.01E+04 | 2.29E+04 | 1.80E+04 | 1.71E+04 | 2.12E+04 | 2.35E+04 | 2.07E+04 | 2.21E+04 | 1.18E+04 | 1.41E+04 | 1.30E+04 | 1.92E+04 | 1.93E+04 | 1.93E+04 |
| pmb0771 | 3.14E+02 | 1.78E+02 | 4.91E+00 | 3.13E+02 | [M+H]+ | C02717 | N-Feruloyl tyramine | Phenolamides | 2.98E+06 | 2.67E+06 | 2.62E+06 | 2.55E+06 | 2.40E+06 | 2.48E+06 | 1.65E+06 | 1.25E+06 | 1.45E+06 | 1.20E+06 | 2.16E+06 | 1.68E+06 | 3.67E+06 | 2.89E+06 | 3.28E+06 |
| pmb0774 | 1.77E+02 | 1.61E+02 | 1.63E+00 | 1.76E+02 | [M+H]+ | C17203 | N-hydroxy tryptamine | Tryptamine derivatives | 2.97E+07 | 3.07E+07 | 2.69E+07 | 2.66E+07 | 3.01E+07 | 2.42E+07 | 3.53E+07 | 5.14E+07 | 4.34E+07 | 2.34E+07 | 1.57E+07 | 1.96E+07 | 1.33E+07 | 2.74E+07 | 2.04E+07 |
| pmb0782 | 8.61E+01 | 5.60E+01 | 1.19E+00 | 8.51E+01 | [M+H]+ | C01746 | Piperidine | Alkaloids | 6.72E+06 | 9.29E+06 | 9.33E+06 | 9.51E+06 | 1.01E+07 | 8.95E+06 | 9.32E+06 | 9.10E+06 | 9.21E+06 | 7.24E+06 | 9.40E+06 | 8.32E+06 | 1.11E+07 | 1.10E+07 | 1.11E+07 |
| pmb0785 | 1.30E+02 | 1.28E+02 | 4.71E+00 | 1.29E+02 | [M+H]+ | C06323 | Isoquinoline | Alkaloids | 4.85E+05 | 5.28E+05 | 5.70E+05 | 5.75E+05 | 5.59E+05 | 6.25E+05 | 2.57E+05 | 2.71E+05 | 2.64E+05 | 1.46E+05 | 1.92E+05 | 1.69E+05 | 1.61E+05 | 2.09E+05 | 1.85E+05 |
| pmb0786 | 1.80E+02 | 8.42E+01 | 6.90E-01 | 1.79E+02 | [M+H]+ | C00329 | Glucosamine | Carbohydrates | 6.66E+05 | 3.93E+05 | 6.03E+05 | 4.66E+05 | 7.18E+05 | 6.22E+05 | 6.12E+05 | 8.44E+05 | 7.28E+05 | 3.46E+05 | 7.89E+05 | 5.68E+05 | 1.12E+06 | 6.49E+05 | 8.85E+05 |
| pmb0789 | 3.32E+02 | 3.15E+02 | 1.16E+00 | 3.31E+02 | [M+H]+ | - | Pyridoxine O-glucoside | Vitamins | 1.99E+07 | 1.94E+07 | 1.93E+07 | 1.91E+07 | 1.86E+07 | 1.81E+07 | 3.16E+07 | 3.13E+07 | 3.15E+07 | 3.07E+07 | 2.65E+07 | 2.86E+07 | 1.87E+07 | 1.95E+07 | 1.91E+07 |
| pmb0800 | 1.23E+02 | 8.03E+01 | 1.52E+00 | 1.22E+02 | [M+H]+ | C00153 | Niacinamide | Vitamins | 5.97E+06 | 7.65E+06 | 8.03E+06 | 8.14E+06 | 8.08E+06 | 8.47E+06 | 8.04E+06 | 7.75E+06 | 7.90E+06 | 3.75E+06 | 6.10E+06 | 4.93E+06 | 5.26E+06 | 4.67E+06 | 4.97E+06 |
| pmb0801 | 3.46E+02 | 1.85E+02 | 1.58E+00 | 3.45E+02 | [M+H]+ | - | 4-Pyridoxic acid O-hexoside | Vitamins | 3.06E+06 | 3.29E+06 | 3.13E+06 | 3.36E+06 | 3.17E+06 | 3.12E+06 | 2.60E+06 | 2.44E+06 | 2.52E+06 | 5.45E+06 | 3.19E+06 | 4.32E+06 | 3.10E+06 | 2.86E+06 | 2.98E+06 |
| pmb0802 | 2.20E+02 | 2.03E+02 | 2.15E+00 | 2.19E+02 | [M+H]+ | C00864 | D-Pantothenic acid | Vitamins | 1.25E+07 | 1.39E+07 | 1.33E+07 | 1.28E+07 | 1.25E+07 | 1.16E+07 | 1.75E+07 | 1.66E+07 | 1.71E+07 | 1.61E+07 | 1.29E+07 | 1.45E+07 | 1.14E+07 | 1.14E+07 | 1.14E+07 |
| pmb0818 | 2.06E+02 | 1.48E+02 | 2.39E+00 | 2.05E+02 | [M+H]+ | C05660 | Methoxyindoleacetic acid | Indole derivatives | 9.68E+06 | 1.15E+07 | 1.14E+07 | 1.17E+07 | 1.15E+07 | 9.85E+06 | 1.14E+07 | 1.20E+07 | 1.17E+07 | 1.46E+07 | 1.28E+07 | 1.37E+07 | 1.78E+07 | 1.39E+07 | 1.59E+07 |
| pmb0819 | 1.57E+02 | 1.30E+02 | 2.39E+00 | 1.56E+02 | [M+H]+ | C02938 | 3-Indoleacetonitrile | Indole derivatives | 8.57E+05 | 7.23E+05 | 8.09E+05 | 8.40E+05 | 9.49E+05 | 1.01E+06 | 8.77E+05 | 9.64E+05 | 9.21E+05 | 1.31E+06 | 1.16E+06 | 1.24E+06 | 1.40E+06 | 1.16E+06 | 1.28E+06 |
| pmb0824 | 5.37E+02 | 1.78E+02 | 2.68E+00 | 5.36E+02 | [M+H]+ | - | Syringic acid O-feruloyl-O-hexoside | Benzoic acid derivatives | 6.41E+04 | 7.02E+04 | 6.23E+04 | 7.41E+04 | 8.80E+04 | 6.51E+04 | 5.10E+04 | 8.02E+04 | 6.56E+04 | 1.43E+05 | 7.59E+04 | 1.09E+05 | 1.02E+05 | 1.06E+05 | 1.04E+05 |
| pmb0835 | 6.11E+02 | 6.11E+02 | 2.36E+00 | 6.10E+02 | [M+H]+ | - | Gallocatechin-gallocatechin | Catechin derivatives | 1.24E+05 | 1.25E+05 | 1.16E+05 | 1.19E+05 | 1.12E+05 | 8.63E+04 | 1.94E+04 | 3.94E+04 | 2.94E+04 | 2.56E+04 | 4.94E+04 | 3.75E+04 | 1.06E+05 | 1.02E+05 | 1.04E+05 |
| pmb0848 | 4.94E+02 | 1.85E+02 | 7.88E+00 | 4.93E+02 | [M+H]+ | - | LysoPC 16:1 (2n isomer) | Lipids_Glycerophospholipids | 1.07E+06 | 1.09E+06 | 1.03E+06 | 1.01E+06 | 9.33E+05 | 9.48E+05 | 1.70E+05 | 1.80E+05 | 1.75E+05 | 1.08E+06 | 3.52E+05 | 7.16E+05 | 2.20E+06 | 4.11E+06 | 3.16E+06 |
| pmb0852 | 5.20E+02 | 5.02E+02 | 8.43E+00 | 5.19E+02 | [M+H]+ | - | LysoPC 18:2 | Lipids_Glycerophospholipids | 1.91E+07 | 1.97E+07 | 2.03E+07 | 2.07E+07 | 2.07E+07 | 2.13E+07 | 1.38E+07 | 1.40E+07 | 1.39E+07 | 1.64E+07 | 1.27E+07 | 1.46E+07 | 1.50E+07 | 2.72E+07 | 2.11E+07 |
| pmb0854 | 5.18E+02 | 4.60E+02 | 8.79E+00 | 5.17E+02 | [M+H]+ | - | LysoPC 18:3 | Lipids_Glycerophospholipids | 1.36E+07 | 1.53E+07 | 1.50E+07 | 1.45E+07 | 1.50E+07 | 1.45E+07 | 1.49E+07 | 1.52E+07 | 1.51E+07 | 2.02E+07 | 1.30E+07 | 1.66E+07 | 1.77E+07 | 1.60E+07 | 1.69E+07 |
| pmb0855 | 4.96E+02 | 4.78E+02 | 8.80E+00 | 4.95E+02 | [M+H]+ | - | LysoPC 16:0 | Lipids_Glycerophospholipids | 2.55E+06 | 2.55E+06 | 2.81E+06 | 2.66E+06 | 2.52E+06 | 2.65E+06 | 2.41E+06 | 2.64E+06 | 2.53E+06 | 3.55E+06 | 2.26E+06 | 2.91E+06 | 3.18E+06 | 2.93E+06 | 3.06E+06 |
| pmb0856 | 4.81E+02 | 3.40E+02 | 8.86E+00 | 4.80E+02 | [M+H]+ | - | LysoPE 18:1 (2n isomer) | Lipids_Glycerophospholipids | 2.97E+06 | 3.08E+06 | 3.03E+06 | 3.05E+06 | 2.85E+06 | 3.06E+06 | 8.72E+05 | 8.08E+05 | 8.40E+05 | 1.11E+06 | 1.12E+06 | 1.12E+06 | 2.07E+06 | 1.40E+07 | 8.04E+06 |
| pmb0859 | 5.22E+02 | 1.85E+02 | 8.91E+00 | 5.21E+02 | [M+H]+ | - | LysoPC 18:1 (2n isomer) | Lipids_Glycerophospholipids | 1.71E+07 | 1.60E+07 | 1.60E+07 | 1.51E+07 | 1.45E+07 | 1.47E+07 | 4.16E+06 | 4.13E+06 | 4.15E+06 | 7.34E+06 | 6.55E+06 | 6.95E+06 | 1.77E+07 | 3.73E+07 | 2.75E+07 |
| pmb0862 | 4.40E+02 | 4.23E+02 | 6.77E+00 | 4.39E+02 | [M+H]+ | - | LysoPC 12:1 | Lipids_Glycerophospholipids | 4.38E+04 | 4.25E+04 | 3.79E+04 | 3.86E+04 | 3.73E+04 | 3.88E+04 | 5.56E+04 | 5.66E+04 | 5.61E+04 | 1.30E+04 | 3.25E+04 | 2.28E+04 | 2.36E+04 | 2.28E+04 | 2.32E+04 |
| pmb0863 | 4.92E+02 | 1.85E+02 | 7.34E+00 | 4.91E+02 | [M+H]+ | - | LysoPC 16:2 (2n isomer) | Lipids_Glycerophospholipids | 1.08E+07 | 1.06E+07 | 1.04E+07 | 9.88E+06 | 9.75E+06 | 9.74E+06 | 2.04E+06 | 1.94E+06 | 1.99E+06 | 1.73E+07 | 4.19E+06 | 1.07E+07 | 2.59E+07 | 5.27E+07 | 3.93E+07 |
| pmb0864 | 4.26E+02 | 2.86E+02 | 7.69E+00 | 4.25E+02 | [M+H]+ | - | LysoPE 14:0 | Lipids_Glycerophospholipids | 1.36E+05 | 1.35E+05 | 1.31E+05 | 1.39E+05 | 1.33E+05 | 1.23E+05 | 1.19E+05 | 1.25E+05 | 1.22E+05 | 1.30E+05 | 1.01E+05 | 1.16E+05 | 1.13E+05 | 1.15E+05 | 1.14E+05 |
| pmb0865 | 5.18E+02 | 5.00E+02 | 7.71E+00 | 5.17E+02 | [M+H]+ | - | LysoPC 18:3 (2n isomer) | Lipids_Glycerophospholipids | 1.41E+06 | 1.46E+06 | 1.50E+06 | 1.40E+06 | 1.39E+06 | 1.37E+06 | 2.57E+05 | 2.63E+05 | 2.60E+05 | 1.55E+06 | 4.58E+05 | 1.00E+06 | 2.36E+06 | 4.71E+06 | 3.54E+06 |
| pmb0866 | 4.68E+02 | 4.51E+02 | 7.74E+00 | 4.67E+02 | [M+H]+ | - | LysoPC 14:0 | Lipids_Glycerophospholipids | 8.64E+05 | 8.47E+05 | 8.25E+05 | 8.05E+05 | 7.54E+05 | 7.61E+05 | 6.78E+05 | 6.90E+05 | 6.84E+05 | 7.46E+05 | 6.08E+05 | 6.77E+05 | 6.44E+05 | 6.60E+05 | 6.52E+05 |
| pmb0873 | 5.20E+02 | 1.25E+02 | 8.25E+00 | 5.19E+02 | [M+H]+ | - | LysoPC 18:2 (2n isomer) | Lipids_Glycerophospholipids | 1.68E+06 | 1.64E+06 | 1.64E+06 | 1.55E+06 | 1.50E+06 | 1.45E+06 | 5.32E+05 | 5.08E+05 | 5.20E+05 | 1.40E+06 | 8.09E+05 | 1.10E+06 | 2.10E+06 | 3.69E+06 | 2.90E+06 |
| pmb0874 | 4.79E+02 | 3.38E+02 | 8.38E+00 | 4.78E+02 | [M+H]+ | - | LysoPE 18:2 (2n isomer) | Lipids_Glycerophospholipids | 6.73E+06 | 6.75E+06 | 7.31E+06 | 7.00E+06 | 7.21E+06 | 8.30E+06 | 4.87E+06 | 4.92E+06 | 4.90E+06 | 5.50E+06 | 4.66E+06 | 5.08E+06 | 3.85E+06 | 1.10E+07 | 7.43E+06 |
| pmb0876 | 4.54E+02 | 3.14E+02 | 8.74E+00 | 4.53E+02 | [M+H]+ | - | LysoPE 16:0 | Lipids_Glycerophospholipids | 2.64E+07 | 2.42E+07 | 2.51E+07 | 2.41E+07 | 2.30E+07 | 2.34E+07 | 3.61E+07 | 3.72E+07 | 3.67E+07 | 2.96E+07 | 2.29E+07 | 2.63E+07 | 2.43E+07 | 2.46E+07 | 2.45E+07 |
| pmb0880 | 4.82E+02 | 3.14E+02 | 8.98E+00 | 4.81E+02 | [M+H]+ | - | LysoPE 18:0 (2n isomer) | Lipids_Glycerophospholipids | 2.44E+03 | 1.33E+03 | 9.00E+00 | 3.13E+03 | 2.01E+03 | 1.78E+03 | 2.78E+03 | 1.72E+03 | 2.25E+03 | 1.76E+03 | 9.00E+00 | 8.85E+02 | 1.30E+03 | 1.28E+03 | 1.29E+03 |
| pmb0882 | 5.22E+02 | 1.26E+02 | 9.07E+00 | 5.21E+02 | [M+H]+ | - | LysoPC 18:1 | Lipids_Glycerophospholipids | 2.80E+06 | 2.83E+06 | 2.90E+06 | 2.74E+06 | 2.56E+06 | 2.67E+06 | 6.87E+05 | 7.38E+05 | 7.13E+05 | 1.29E+06 | 1.14E+06 | 1.22E+06 | 2.83E+06 | 5.84E+06 | 4.34E+06 |
| pmb0883 | 4.82E+02 | 3.42E+02 | 9.86E+00 | 4.81E+02 | [M+H]+ | - | LysoPE 18:0 | Lipids_Glycerophospholipids | 9.04E+04 | 9.50E+04 | 9.47E+04 | 9.68E+04 | 8.17E+04 | 8.95E+04 | 2.65E+05 | 2.87E+05 | 2.76E+05 | 1.18E+05 | 9.90E+04 | 1.09E+05 | 7.13E+04 | 1.13E+05 | 9.22E+04 |
| pmb0889 | 2.79E+02 | 2.02E+02 | 8.96E+00 | 2.78E+02 | [M+H]+ | C08364 | Punicic acid | Lipids_Fatty acids | 1.87E+07 | 1.70E+07 | 1.76E+07 | 1.75E+07 | 1.62E+07 | 1.69E+07 | 1.25E+07 | 1.30E+07 | 1.28E+07 | 1.61E+07 | 1.41E+07 | 1.51E+07 | 7.56E+06 | 3.50E+07 | 2.13E+07 |
| pmb0890 | 3.55E+02 | 3.38E+02 | 1.05E+01 | 3.54E+02 | [M+H]+ | - | MAG (18:2) | Lipids_Glycerolipids | 3.91E+07 | 2.83E+07 | 2.83E+07 | 2.64E+07 | 2.44E+07 | 2.63E+07 | 3.14E+07 | 3.30E+07 | 3.22E+07 | 4.70E+07 | 3.54E+07 | 4.12E+07 | 2.41E+07 | 3.96E+07 | 3.19E+07 |
| pmb0896 | 2.65E+02 | 1.78E+02 | 2.33E+00 | 2.64E+02 | [M+H]+ | - | N'-Feruloyl putrescine | Phenolamides | 6.18E+06 | 6.76E+06 | 6.31E+06 | 6.52E+06 | 7.21E+06 | 5.75E+06 | 8.93E+06 | 8.62E+06 | 8.78E+06 | 6.00E+06 | 7.02E+06 | 6.51E+06 | 1.02E+07 | 1.01E+07 | 1.02E+07 |
| pmb0962 | 2.35E+02 | 1.18E+02 | 8.00E-01 | 2.34E+02 | [M+H]+ | - | Lysine butyrate | Amino acid derivatives | 1.88E+06 | 1.86E+06 | 1.80E+06 | 1.92E+06 | 1.98E+06 | 1.69E+06 | 1.39E+06 | 1.58E+06 | 1.49E+06 | 1.64E+06 | 1.57E+06 | 1.61E+06 | 1.74E+06 | 1.51E+06 | 1.63E+06 |
| pmb0964 | 3.66E+02 | 2.04E+02 | 8.00E-01 | 3.65E+02 | [M+H]+ | - | iP7G | Nucleotide and its derivates | 7.64E+06 | 7.88E+06 | 9.30E+06 | 7.68E+06 | 9.00E+06 | 9.39E+06 | 1.02E+07 | 1.03E+07 | 1.03E+07 | 7.47E+06 | 6.04E+06 | 6.76E+06 | 5.58E+06 | 5.60E+06 | 5.59E+06 |
| pmb0981 | 3.48E+02 | 1.36E+02 | 1.16E+00 | 3.47E+02 | [M+H]+ | C00020 | Adenosine 5'-monophosphate | Nucleotide and its derivates | 1.26E+07 | 1.42E+07 | 1.40E+07 | 1.49E+07 | 1.56E+07 | 1.30E+07 | 4.19E+06 | 3.41E+06 | 3.80E+06 | 5.16E+06 | 5.75E+06 | 5.46E+06 | 3.75E+06 | 2.83E+06 | 3.29E+06 |
| pmb0998 | 3.64E+02 | 1.53E+02 | 1.01E+00 | 3.63E+02 | [M+H]+ | C00144 | Guanosine 5'-monophosphate | Nucleotide and its derivates | 6.11E+05 | 6.99E+05 | 6.62E+05 | 6.18E+05 | 6.66E+05 | 5.95E+05 | 3.68E+05 | 3.10E+05 | 3.39E+05 | 2.25E+05 | 3.95E+05 | 3.10E+05 | 3.12E+05 | 1.88E+05 | 2.50E+05 |
| pmb1096 | 1.18E+02 | 5.82E+01 | 2.39E+00 | 1.17E+02 | [M+H]+ | C00463 | Indole | Indole derivatives | 1.42E+07 | 1.76E+07 | 1.67E+07 | 1.76E+07 | 1.59E+07 | 1.52E+07 | 1.76E+07 | 1.78E+07 | 1.77E+07 | 2.14E+07 | 1.85E+07 | 2.00E+07 | 2.81E+07 | 2.31E+07 | 2.56E+07 |
| pmb1240 | 5.03E+02 | 3.12E+02 | 3.79E+00 | 5.02E+02 | [M+H]+ | - | Phellodensin F | Others | 2.08E+05 | 1.71E+05 | 1.93E+05 | 1.73E+05 | 1.59E+05 | 1.53E+05 | 2.43E+05 | 2.09E+05 | 2.26E+05 | 1.03E+05 | 1.06E+05 | 1.05E+05 | 5.41E+04 | 5.58E+04 | 5.50E+04 |
| pmb1283 | 4.48E+02 | 2.16E+02 | 4.16E+00 | 4.47E+02 | [M+H]+ | - | L-Glutaminyl-L-valyl-L-valyl-L-cysteine | Amino acid derivatives | 2.98E+04 | 2.98E+04 | 3.26E+04 | 3.28E+04 | 3.46E+04 | 2.27E+04 | 3.46E+04 | 4.07E+04 | 3.77E+04 | 3.74E+04 | 3.32E+04 | 3.53E+04 | 9.00E+00 | 2.49E+04 | 1.25E+04 |
| pmb1318 | 4.57E+02 | 1.92E+02 | 4.48E+00 | 4.56E+02 | [M+H]+ | - | 8-Methyl-2-oxo-4-phenyl-2H-chromen-7-yl 4-(hexyloxy)benzoate | Benzoic acid derivatives | 1.62E+06 | 1.51E+06 | 1.25E+06 | 1.33E+06 | 1.30E+06 | 1.29E+06 | 1.68E+06 | 1.64E+06 | 1.66E+06 | 2.24E+06 | 1.96E+06 | 2.10E+06 | 1.77E+06 | 1.69E+06 | 1.73E+06 |
| pmb1452 | 2.75E+02 | 2.58E+02 | 6.23E+00 | 2.74E+02 | [M+H]+ | - | N-Lauryldiethanolamine | Others | 9.31E+05 | 1.05E+06 | 9.34E+05 | 1.06E+06 | 1.04E+06 | 9.51E+05 | 1.22E+06 | 1.13E+06 | 1.18E+06 | 7.68E+05 | 1.05E+06 | 9.09E+05 | 1.34E+06 | 1.13E+06 | 1.24E+06 |
| pmb1562 | 3.51E+02 | 2.60E+02 | 7.80E+00 | 3.50E+02 | [M+H]+ | - | MAG (18:4) isomer3 | Lipids_Glycerolipids | 1.84E+04 | 2.06E+04 | 2.35E+04 | 1.65E+04 | 2.08E+04 | 2.27E+04 | 3.57E+04 | 3.96E+04 | 3.77E+04 | 2.36E+04 | 2.69E+04 | 2.53E+04 | 1.41E+04 | 1.53E+04 | 1.47E+04 |
| pmb1574 | 2.77E+02 | 1.38E+02 | 8.21E+00 | 2.76E+02 | [M+H]+ | - | Octadecadien-6-ynoic acid | Lipids_Fatty acids | 8.85E+05 | 8.63E+05 | 8.51E+05 | 8.40E+05 | 8.22E+05 | 8.28E+05 | 1.11E+06 | 1.14E+06 | 1.13E+06 | 1.01E+06 | 1.10E+06 | 1.06E+06 | 5.14E+05 | 9.76E+05 | 7.45E+05 |
| pmb1605 | 3.53E+02 | 3.36E+02 | 8.45E+00 | 3.52E+02 | [M+H]+ | - | MAG (18:3) isomer3 | Lipids_Glycerolipids | 4.54E+06 | 4.21E+06 | 4.23E+06 | 4.14E+06 | 3.79E+06 | 4.00E+06 | 6.94E+06 | 7.04E+06 | 6.99E+06 | 8.40E+06 | 5.73E+06 | 7.07E+06 | 5.91E+06 | 1.24E+07 | 9.16E+06 |
| pmb1626 | 5.34E+02 | 2.63E+02 | 9.22E+00 | 5.33E+02 | [M+H]+ | - | MGMG (18:2) isomer1 | Lipids_Glycerolipids | 7.72E+06 | 7.45E+06 | 6.76E+06 | 7.34E+06 | 7.37E+06 | 6.67E+06 | 9.15E+06 | 1.05E+07 | 9.83E+06 | 1.38E+07 | 1.07E+07 | 1.23E+07 | 8.90E+06 | 2.33E+07 | 1.61E+07 |
| pmb1650 | 2.79E+02 | 1.49E+02 | 9.71E+00 | 2.78E+02 | [M+H]+ | - | "Octadeca-11E,13E,15Z-trienoic acid" | Lipids_Fatty acids | 2.12E+07 | 1.91E+07 | 2.04E+07 | 2.05E+07 | 1.86E+07 | 2.05E+07 | 1.68E+07 | 1.68E+07 | 1.68E+07 | 1.73E+07 | 1.76E+07 | 1.75E+07 | 1.92E+07 | 1.96E+07 | 1.94E+07 |
| pmb1652 | 1.49E+02 | 1.21E+02 | 9.71E+00 | 1.48E+02 | [M+H]+ | - | Phthalic anhydride | Others | 2.76E+06 | 2.47E+06 | 2.59E+06 | 2.50E+06 | 2.39E+06 | 2.61E+06 | 2.26E+06 | 2.36E+06 | 2.31E+06 | 2.31E+06 | 2.41E+06 | 2.36E+06 | 2.65E+06 | 2.47E+06 | 2.56E+06 |
| pmb1656 | 3.53E+02 | 1.74E+02 | 9.90E+00 | 3.52E+02 | [M+H]+ | - | MAG (18:3) isomer4 | Lipids_Glycerolipids | 3.55E+05 | 2.64E+05 | 2.80E+05 | 2.67E+05 | 2.39E+05 | 2.62E+05 | 3.74E+05 | 3.55E+05 | 3.65E+05 | 5.30E+05 | 3.47E+05 | 4.39E+05 | 2.67E+05 | 4.62E+05 | 3.65E+05 |
| pmb1912 | 4.74E+02 | 3.28E+02 | 2.30E+00 | 4.73E+02 | [M+H]+ | C00234 | 10-Formyl-THF | Others | 1.34E+06 | 1.43E+06 | 1.35E+06 | 1.03E+06 | 1.18E+06 | 1.53E+06 | 1.22E+06 | 1.52E+06 | 1.37E+06 | 4.72E+06 | 2.59E+06 | 3.66E+06 | 3.07E+06 | 2.97E+06 | 3.02E+06 |
| pmb2211 | 3.43E+02 | 2.41E+02 | 6.59E+00 | 3.42E+02 | [M+H]+ | - | Cocamidopropyl betaine | Others | 1.58E+06 | 1.50E+06 | 1.46E+06 | 1.38E+06 | 1.38E+06 | 1.39E+06 | 1.49E+06 | 1.39E+06 | 1.44E+06 | 1.31E+06 | 1.30E+06 | 1.31E+06 | 2.76E+06 | 1.57E+06 | 2.17E+06 |
| pmb2221 | 3.18E+02 | 3.01E+02 | 6.95E+00 | 3.17E+02 | [M+H]+ | C12144 | 4-Hydroxysphinganine | Lipids_Fatty acids | 8.17E+06 | 8.07E+06 | 8.30E+06 | 7.81E+06 | 7.80E+06 | 7.54E+06 | 8.25E+06 | 7.92E+06 | 8.09E+06 | 7.07E+06 | 6.25E+06 | 6.66E+06 | 7.09E+06 | 6.79E+06 | 6.94E+06 |
| pmb2222 | 3.19E+02 | 3.02E+02 | 7.07E+00 | 3.18E+02 | [M+H]+ | - | Phytocassane C | Terpenoids | 5.16E+05 | 5.24E+05 | 5.58E+05 | 4.91E+05 | 5.44E+05 | 5.26E+05 | 5.15E+05 | 5.37E+05 | 5.26E+05 | 4.57E+05 | 3.86E+05 | 4.22E+05 | 4.70E+05 | 4.34E+05 | 4.52E+05 |
| pmb2228 | 5.38E+02 | 1.85E+02 | 6.84E+00 | 5.37E+02 | [M+H]+ | - | LysoPC 19:0 | Lipids_Glycerophospholipids | 1.52E+05 | 1.43E+05 | 1.53E+05 | 1.59E+05 | 1.34E+05 | 1.25E+05 | 8.44E+04 | 8.23E+04 | 8.34E+04 | 1.36E+05 | 8.66E+04 | 1.11E+05 | 1.81E+05 | 3.95E+05 | 2.88E+05 |
| pmb2251 | 6.94E+02 | 3.36E+02 | 7.60E+00 | 6.93E+02 | [M+H]+ | - | DGMG (18:1) | Lipids_Glycerolipids | 4.46E+05 | 3.95E+05 | 3.70E+05 | 3.91E+05 | 3.54E+05 | 3.39E+05 | 3.20E+05 | 3.19E+05 | 3.20E+05 | 2.42E+05 | 2.09E+05 | 2.26E+05 | 3.13E+05 | 3.90E+06 | 2.11E+06 |
| pmb2260 | 4.80E+02 | 1.85E+02 | 7.76E+00 | 4.79E+02 | [M+H]+ | - | LysoPC 15:1 | Lipids_Glycerophospholipids | 8.88E+04 | 9.41E+04 | 8.65E+04 | 1.00E+05 | 9.21E+04 | 8.98E+04 | 9.34E+04 | 8.67E+04 | 9.01E+04 | 1.02E+05 | 7.85E+04 | 9.03E+04 | 8.33E+04 | 1.03E+05 | 9.32E+04 |
| pmb2319 | 4.82E+02 | 1.85E+02 | 8.26E+00 | 4.81E+02 | [M+H]+ | - | LysoPC 15:0 | Lipids_Glycerophospholipids | 5.99E+05 | 5.76E+05 | 6.32E+05 | 5.90E+05 | 5.84E+05 | 5.30E+05 | 5.83E+05 | 6.35E+05 | 6.09E+05 | 7.32E+05 | 5.43E+05 | 6.38E+05 | 4.34E+05 | 2.89E+05 | 3.62E+05 |
| pmb2325 | 3.53E+02 | 2.62E+02 | 8.42E+00 | 3.52E+02 | [M+H]+ | - | MAG (18:3) isomer2 | Lipids_Glycerolipids | 6.38E+06 | 5.73E+06 | 6.17E+06 | 5.64E+06 | 5.36E+06 | 6.01E+06 | 9.66E+06 | 1.02E+07 | 9.93E+06 | 1.16E+07 | 8.16E+06 | 9.88E+06 | 8.07E+06 | 1.52E+07 | 1.16E+07 |
| pmb2363 | 3.57E+02 | 3.40E+02 | 8.76E+00 | 3.56E+02 | [M+H]+ | - | MAG (18:1) isomer1 | Lipids_Glycerolipids | 1.06E+06 | 9.25E+05 | 9.24E+05 | 9.46E+05 | 8.54E+05 | 8.75E+05 | 4.35E+05 | 3.94E+05 | 4.15E+05 | 6.88E+05 | 6.18E+05 | 6.53E+05 | 8.62E+05 | 4.40E+06 | 2.63E+06 |
| pmb2369 | 7.72E+02 | 4.78E+02 | 8.79E+00 | 7.71E+02 | [M+H]+ | - | PC 19:2/16:0 | Lipids_Glycerophospholipids | 3.07E+03 | 5.02E+03 | 7.07E+03 | 7.76E+03 | 6.29E+03 | 6.50E+03 | 5.04E+03 | 5.08E+03 | 5.06E+03 | 1.07E+04 | 4.21E+03 | 7.46E+03 | 9.84E+03 | 6.46E+03 | 8.15E+03 |
| pmb2383 | 5.34E+02 | 3.38E+02 | 9.04E+00 | 5.33E+02 | [M+H]+ | - | MGMG (18:2) isomer2 | Lipids_Glycerolipids | 2.54E+06 | 2.31E+06 | 2.61E+06 | 2.44E+06 | 2.23E+06 | 2.23E+06 | 3.10E+06 | 2.97E+06 | 3.04E+06 | 4.24E+06 | 3.84E+06 | 4.04E+06 | 3.05E+06 | 7.31E+06 | 5.18E+06 |
| pmb2388 | 5.24E+02 | 5.07E+02 | 8.91E+00 | 5.23E+02 | [M+H]+ | - | LysoPC 18:0 (2n isomer) | Lipids_Glycerophospholipids | 2.56E+06 | 2.37E+06 | 2.45E+06 | 2.36E+06 | 2.21E+06 | 2.38E+06 | 5.93E+05 | 6.13E+05 | 6.03E+05 | 1.10E+06 | 9.76E+05 | 1.04E+06 | 2.45E+06 | 5.46E+06 | 3.96E+06 |
| pmb2406 | 5.10E+02 | 5.10E+02 | 9.34E+00 | 5.09E+02 | [M+H]+ | - | LysoPC 17:0 | Lipids_Glycerophospholipids | 1.11E+06 | 1.27E+06 | 1.18E+06 | 1.21E+06 | 1.20E+06 | 1.17E+06 | 1.74E+06 | 1.77E+06 | 1.76E+06 | 1.78E+06 | 8.89E+05 | 1.33E+06 | 1.11E+06 | 1.35E+06 | 1.23E+06 |
| pmb2444 | 3.53E+02 | 2.62E+02 | 1.01E+01 | 3.52E+02 | [M+H]+ | - | MAG (18:3) isomer1 | Lipids_Glycerolipids | 5.03E+06 | 4.66E+06 | 5.23E+06 | 5.16E+06 | 4.57E+06 | 5.34E+06 | 4.56E+06 | 4.29E+06 | 4.43E+06 | 7.23E+06 | 5.14E+06 | 6.19E+06 | 3.09E+06 | 3.19E+06 | 3.14E+06 |
| pmb2497 | 1.97E+02 | 1.35E+02 | 7.10E-01 | 1.98E+02 | [M-H]- | C05584 | 4-Hydroxy-3-methoxymandelate | Organic acids | 8.38E+04 | 7.76E+04 | 7.75E+04 | 8.87E+04 | 8.25E+04 | 8.87E+04 | 1.08E+05 | 1.15E+05 | 1.12E+05 | 9.52E+04 | 9.34E+04 | 9.43E+04 | 8.52E+04 | 8.85E+04 | 8.69E+04 |
| pmb2507 | 2.13E+02 | 7.89E+01 | 9.20E-01 | 2.14E+02 | [M-H]- | C00672 | 2-Deoxyribose 1-phosphate | Carbohydrates | 4.12E+04 | 3.81E+04 | 3.92E+04 | 4.10E+04 | 3.12E+04 | 2.75E+04 | 3.19E+04 | 3.01E+04 | 3.10E+04 | 2.95E+04 | 3.15E+04 | 3.05E+04 | 2.61E+04 | 4.24E+04 | 3.43E+04 |
| pmb2554 | 5.29E+02 | 3.56E+02 | 2.16E+00 | 5.30E+02 | [M-H]- | - | 5-O-Feruloyl quinic acid glucoside | Quinate and its derivatives | 7.06E+04 | 6.87E+04 | 6.98E+04 | 7.00E+04 | 7.42E+04 | 6.90E+04 | 5.49E+04 | 5.99E+04 | 5.74E+04 | 8.23E+04 | 9.45E+04 | 8.84E+04 | 6.83E+04 | 8.29E+04 | 7.56E+04 |
| pmb2561 | 1.90E+02 | 8.40E+01 | 2.37E+00 | 1.91E+02 | [M-H]- | C02712 | N-Acetylmethionine | Amino acid derivatives | 3.50E+04 | 2.95E+04 | 4.05E+04 | 2.62E+04 | 2.91E+04 | 3.79E+04 | 4.07E+04 | 4.77E+04 | 4.42E+04 | 9.36E+04 | 5.19E+04 | 7.28E+04 | 7.99E+04 | 1.33E+05 | 1.06E+05 |
| pmb2586 | 5.93E+02 | 5.49E+02 | 3.72E+00 | 5.94E+02 | [M-H]- | - | Gallocatechin-catechin | Catechin derivatives | 6.89E+05 | 6.71E+05 | 7.03E+05 | 6.74E+05 | 7.16E+05 | 7.21E+05 | 4.94E+05 | 4.52E+05 | 4.73E+05 | 6.64E+05 | 4.49E+05 | 5.57E+05 | 6.15E+05 | 6.49E+05 | 6.32E+05 |
| pmb2591 | 2.45E+02 | 2.03E+02 | 3.87E+00 | 2.46E+02 | [M-H]- | C03137 | Acetyl tryptophan | Amino acid derivatives | 4.86E+06 | 4.38E+06 | 4.29E+06 | 4.14E+06 | 3.90E+06 | 3.95E+06 | 4.96E+06 | 4.45E+06 | 4.71E+06 | 3.44E+06 | 3.58E+06 | 3.51E+06 | 3.41E+06 | 4.27E+06 | 3.84E+06 |
| pmb2620 | 2.07E+02 | 1.92E+02 | 5.52E+00 | 2.08E+02 | [M-H]- | - | "3,4-Dimethoxycinnamic acid" | Hydroxycinnamoyl derivatives | 1.57E+06 | 1.64E+06 | 1.80E+06 | 1.78E+06 | 1.96E+06 | 1.89E+06 | 1.43E+06 | 1.41E+06 | 1.42E+06 | 1.31E+06 | 1.14E+06 | 1.23E+06 | 7.01E+05 | 8.82E+05 | 7.92E+05 |
| pmb2636 | 3.35E+02 | 2.53E+02 | 6.89E+00 | 3.36E+02 | [M-H]- | - | "8,15-DiHETE" | Lipids_Fatty acids | 1.15E+04 | 1.11E+04 | 1.15E+04 | 1.24E+04 | 1.18E+04 | 1.20E+04 | 1.32E+04 | 1.18E+04 | 1.25E+04 | 1.54E+04 | 1.19E+04 | 1.37E+04 | 1.13E+04 | 1.22E+04 | 1.18E+04 |
| pmb2640 | 1.99E+02 | 1.81E+02 | 9.66E+00 | 2.00E+02 | [M-H]- | C02679 | Lauric acid (C12:0) | Lipids_Fatty acids | 1.52E+04 | 1.43E+04 | 1.58E+04 | 1.44E+04 | 1.49E+04 | 1.61E+04 | 2.11E+04 | 1.56E+04 | 1.84E+04 | 1.91E+04 | 1.70E+04 | 1.81E+04 | 1.86E+04 | 3.14E+04 | 2.50E+04 |
| pmb2643 | 2.25E+02 | 2.07E+02 | 1.01E+01 | 2.26E+02 | [M-H]- | C08322 | Myristoleic acid (C14:1) | Lipids_Fatty acids | 4.16E+04 | 5.59E+04 | 6.03E+04 | 6.31E+04 | 7.41E+04 | 6.67E+04 | 1.18E+05 | 1.17E+05 | 1.18E+05 | 3.91E+04 | 5.52E+04 | 4.72E+04 | 1.11E+05 | 1.56E+05 | 1.34E+05 |
| pmb2653 | 6.49E+02 | 5.03E+02 | 7.60E-01 | 6.50E+02 | [M-H]- | - | D(+)-Melezitose O-rhamnoside | Carbohydrates | 3.40E+05 | 3.49E+05 | 3.01E+05 | 3.64E+05 | 3.49E+05 | 2.78E+05 | 4.42E+05 | 4.35E+05 | 4.39E+05 | 2.13E+05 | 4.33E+05 | 3.23E+05 | 1.56E+05 | 1.48E+05 | 1.52E+05 |
| pmb2654 | 4.60E+02 | 1.18E+02 | 7.80E-01 | 4.61E+02 | [M-H]- | - | Anthranilate O-hexosyl-O-hexoside | Benzoic acid derivatives | 1.97E+06 | 1.54E+06 | 2.27E+06 | 1.68E+06 | 1.54E+06 | 1.83E+06 | 2.54E+06 | 2.06E+06 | 2.30E+06 | 3.96E+06 | 1.76E+06 | 2.86E+06 | 2.75E+06 | 2.41E+06 | 2.58E+06 |
| pmb2657 | 2.89E+02 | 1.91E+02 | 7.10E-01 | 2.90E+02 | [M-H]- | C03406 | Argininosuccinate | Organic acids | 6.12E+05 | 6.30E+05 | 6.24E+05 | 6.04E+05 | 5.72E+05 | 5.94E+05 | 2.41E+05 | 2.50E+05 | 2.46E+05 | 4.58E+05 | 4.30E+05 | 4.44E+05 | 2.63E+05 | 3.23E+05 | 2.93E+05 |
| pmb2673 | 2.10E+02 | 1.35E+02 | 1.50E+00 | 2.11E+02 | [M-H]- | - | "3-(6-Hydroxy-3,4-dioxo-1,5-cyclohexadien-1-yl)-L-alanine" | Amino acid derivatives | 5.96E+04 | 5.53E+04 | 5.57E+04 | 5.27E+04 | 5.20E+04 | 5.26E+04 | 5.43E+04 | 5.28E+04 | 5.36E+04 | 3.81E+04 | 5.21E+04 | 4.51E+04 | 4.35E+04 | 6.30E+04 | 5.33E+04 |
| pmb2684 | 3.28E+02 | 1.34E+02 | 1.68E+00 | 3.29E+02 | [M-H]- | C00575 | Cyclic AMP | Nucleotide and its derivates | 3.02E+06 | 2.83E+06 | 2.97E+06 | 2.85E+06 | 2.70E+06 | 2.87E+06 | 7.60E+06 | 6.99E+06 | 7.30E+06 | 1.79E+06 | 6.80E+06 | 4.30E+06 | 8.72E+06 | 4.95E+06 | 6.84E+06 |
| pmb2723 | 4.85E+02 | 3.23E+02 | 3.44E+00 | 4.86E+02 | [M-H]- | - | 4-hydroxycoumarin di-glucoside | Coumarins | 1.58E+05 | 1.36E+05 | 1.21E+05 | 1.13E+05 | 1.33E+05 | 1.11E+05 | 9.00E+00 | 9.00E+00 | 9.00E+00 | 9.00E+00 | 4.94E+04 | 2.47E+04 | 1.12E+05 | 8.20E+04 | 9.70E+04 |
| pmb2778 | 2.95E+02 | 1.83E+02 | 8.96E+00 | 2.96E+02 | [M-H]- | - | "9,10-EODE" | Lipids_Fatty acids | 1.49E+07 | 1.50E+07 | 1.52E+07 | 1.49E+07 | 1.49E+07 | 1.49E+07 | 1.10E+07 | 1.02E+07 | 1.06E+07 | 1.22E+07 | 1.12E+07 | 1.17E+07 | 6.72E+06 | 4.17E+07 | 2.42E+07 |
| pmb2786 | 2.93E+02 | 2.75E+02 | 8.42E+00 | 2.94E+02 | [M-H]- | - | 9-HOTrE | Lipids_Fatty acids | 1.17E+05 | 1.15E+05 | 1.22E+05 | 1.15E+05 | 1.10E+05 | 1.15E+05 | 1.69E+05 | 1.66E+05 | 1.68E+05 | 1.42E+05 | 1.51E+05 | 1.47E+05 | 7.45E+04 | 2.03E+05 | 1.39E+05 |
| pmb2787 | 2.93E+02 | 2.50E+02 | 9.19E+00 | 2.94E+02 | [M-H]- | - | 9-KODE | Lipids_Fatty acids | 7.37E+04 | 7.80E+04 | 8.26E+04 | 8.30E+04 | 8.11E+04 | 8.08E+04 | 4.35E+04 | 3.74E+04 | 4.05E+04 | 2.62E+04 | 4.00E+04 | 3.31E+04 | 2.67E+04 | 2.66E+05 | 1.46E+05 |
| pmb2789 | 3.09E+02 | 2.92E+02 | 7.54E+00 | 3.10E+02 | [M-H]- | - | 13-HpOTrE(r) | Lipids_Fatty acids | 4.00E+05 | 4.18E+05 | 4.24E+05 | 4.02E+05 | 4.04E+05 | 4.10E+05 | 2.95E+05 | 9.20E+04 | 1.94E+05 | 3.08E+05 | 3.63E+05 | 3.36E+05 | 2.48E+05 | 4.76E+05 | 3.62E+05 |
| pmb2791 | 3.09E+02 | 2.91E+02 | 7.55E+00 | 3.10E+02 | [M-H]- | - | 9-HpOTrE | Lipids_Fatty acids | 2.33E+05 | 2.44E+05 | 2.43E+05 | 2.40E+05 | 2.36E+05 | 2.38E+05 | 1.74E+05 | 6.42E+04 | 1.19E+05 | 1.79E+05 | 2.13E+05 | 1.96E+05 | 1.43E+05 | 2.82E+05 | 2.13E+05 |
| pmb2792 | 2.93E+02 | 1.93E+02 | 7.81E+00 | 2.94E+02 | [M-H]- | - | 13-HOTrE(r) | Lipids_Fatty acids | 2.33E+05 | 2.45E+05 | 2.42E+05 | 2.42E+05 | 2.32E+05 | 2.38E+05 | 3.49E+05 | 2.18E+05 | 2.84E+05 | 2.40E+05 | 2.30E+05 | 2.35E+05 | 2.21E+05 | 6.44E+05 | 4.33E+05 |
| pmb2795 | 1.77E+02 | 1.45E+02 | 5.50E+00 | 1.78E+02 | [M-H]- | - | 4-Methoxycinnamic acid | Hydroxycinnamoyl derivatives | 2.88E+06 | 3.06E+06 | 3.23E+06 | 3.26E+06 | 3.47E+06 | 3.56E+06 | 1.48E+06 | 1.45E+06 | 1.47E+06 | 7.59E+05 | 1.22E+06 | 9.90E+05 | 1.14E+06 | 1.51E+06 | 1.33E+06 |
| pmb2799 | 2.95E+02 | 2.78E+02 | 9.74E+00 | 2.96E+02 | [M-H]- | - | "12,13-EODE" | Lipids_Fatty acids | 2.19E+06 | 2.33E+06 | 2.45E+06 | 2.52E+06 | 2.46E+06 | 2.40E+06 | 4.00E+05 | 3.23E+05 | 3.62E+05 | 3.93E+05 | 6.05E+05 | 4.99E+05 | 1.06E+06 | 8.71E+06 | 4.89E+06 |
| pmb2804 | 3.11E+02 | 2.93E+02 | 7.87E+00 | 3.12E+02 | [M-H]- | C04717 | 13-HPODE | Lipids_Fatty acids | 9.51E+05 | 9.46E+05 | 9.80E+05 | 9.01E+05 | 8.71E+05 | 9.07E+05 | 1.33E+06 | 1.18E+06 | 1.26E+06 | 4.17E+05 | 7.64E+05 | 5.91E+05 | 3.49E+05 | 3.41E+06 | 1.88E+06 |
| pmb2819 | 6.65E+02 | 3.23E+02 | 2.16E+00 | 6.66E+02 | [M-H]- | - | O-Caffeoyl maltotriose | Hydroxycinnamoyl derivatives | 9.07E+04 | 7.16E+04 | 7.29E+04 | 6.56E+04 | 6.78E+04 | 7.02E+04 | 3.91E+04 | 3.84E+04 | 3.88E+04 | 3.67E+04 | 4.36E+04 | 4.02E+04 | 4.13E+04 | 4.49E+04 | 4.31E+04 |
| pmb2826 | 1.47E+02 | 8.50E+01 | 1.03E+00 | 1.48E+02 | [M-H]- | C00815 | Citramalate | Organic acids | 2.93E+06 | 3.94E+06 | 3.54E+06 | 3.44E+06 | 3.35E+06 | 5.04E+06 | 3.74E+06 | 2.43E+06 | 3.09E+06 | 3.37E+06 | 2.73E+06 | 3.05E+06 | 3.49E+06 | 2.38E+06 | 2.94E+06 |
| pmb2831 | 3.15E+02 | 1.53E+02 | 2.44E+00 | 3.16E+02 | [M-H]- | - | Protocatechuic acid O-glucoside | Catechin derivatives | 7.31E+05 | 7.52E+05 | 7.34E+05 | 6.80E+05 | 7.65E+05 | 6.50E+05 | 3.97E+04 | 7.78E+04 | 5.88E+04 | 3.20E+05 | 1.16E+05 | 2.18E+05 | 1.25E+05 | 7.16E+05 | 4.21E+05 |
| pmb2833 | 5.29E+02 | 3.29E+02 | 2.13E+00 | 5.30E+02 | [M-H]- | - | 3-O-Feruloyl quinic acid glucoside | Quinate and its derivatives | 6.94E+04 | 6.29E+04 | 6.85E+04 | 6.74E+04 | 6.51E+04 | 6.79E+04 | 5.62E+04 | 5.93E+04 | 5.78E+04 | 8.43E+04 | 7.75E+04 | 8.09E+04 | 7.91E+04 | 8.00E+04 | 7.96E+04 |
| pmb2835 | 1.81E+02 | 1.51E+02 | 3.95E+00 | 1.82E+02 | [M-H]- | - | Syringaldehyde | Hydroxycinnamoyl derivatives | 5.34E+04 | 4.43E+04 | 4.61E+04 | 5.27E+04 | 5.50E+04 | 4.68E+04 | 7.44E+04 | 8.43E+04 | 7.94E+04 | 3.89E+04 | 4.95E+04 | 4.42E+04 | 4.23E+04 | 3.51E+04 | 3.87E+04 |
| pmb2849 | 3.47E+02 | 3.19E+02 | 4.63E+00 | 3.48E+02 | [M-H]- | C01897 | Camptothecin | Alkaloids | 9.72E+03 | 8.17E+03 | 9.45E+03 | 8.66E+03 | 9.05E+03 | 7.94E+03 | 1.77E+04 | 2.45E+04 | 2.11E+04 | 1.57E+04 | 1.89E+04 | 1.73E+04 | 6.77E+03 | 1.40E+04 | 1.04E+04 |
| pmb2855 | 3.07E+02 | 1.45E+02 | 7.70E-01 | 3.08E+02 | [M-H]- | - | L-Glutamine O-hexside | Amino acid derivatives | 1.57E+06 | 1.65E+06 | 1.48E+06 | 1.66E+06 | 1.58E+06 | 1.33E+06 | 2.14E+06 | 1.86E+06 | 2.00E+06 | 3.57E+05 | 1.56E+06 | 9.59E+05 | 5.30E+05 | 6.27E+05 | 5.79E+05 |
| pmb2857 | 3.08E+02 | 1.28E+02 | 8.00E-01 | 3.09E+02 | [M-H]- | - | L-Glutamic acid O-glucoside | Amino acid derivatives | 5.42E+05 | 5.61E+05 | 5.09E+05 | 5.66E+05 | 5.90E+05 | 5.46E+05 | 6.43E+05 | 5.55E+05 | 5.99E+05 | 3.38E+05 | 5.23E+05 | 4.31E+05 | 4.53E+05 | 4.71E+05 | 4.62E+05 |
| pmb2867 | 3.50E+02 | 1.44E+02 | 1.72E+00 | 3.51E+02 | [M-H]- | - | Kynurenic acid O-hexside | Organic acids | 2.05E+05 | 2.20E+05 | 1.96E+05 | 2.05E+05 | 2.11E+05 | 1.90E+05 | 2.34E+05 | 2.04E+05 | 2.19E+05 | 2.81E+05 | 2.34E+05 | 2.58E+05 | 2.24E+05 | 2.98E+05 | 2.61E+05 |
| pmb2871 | 3.15E+02 | 1.52E+02 | 1.84E+00 | 3.16E+02 | [M-H]- | - | "2,5-dihydroxy benzoic acid O-hexside" | Benzoic acid derivatives | 1.36E+07 | 1.23E+07 | 1.21E+07 | 1.18E+07 | 1.09E+07 | 1.23E+07 | 3.81E+06 | 3.31E+06 | 3.56E+06 | 7.16E+06 | 6.03E+06 | 6.60E+06 | 4.92E+06 | 1.47E+07 | 9.81E+06 |
| pmb2873 | 2.14E+02 | 1.34E+02 | 3.65E+00 | 2.15E+02 | [M-H]- | - | 3-(2-Naphthyl)-D-alanine | Amino acid derivatives | 2.07E+05 | 1.81E+05 | 1.94E+05 | 1.85E+05 | 1.80E+05 | 1.65E+05 | 1.51E+05 | 1.39E+05 | 1.45E+05 | 6.32E+04 | 5.06E+05 | 2.85E+05 | 1.46E+05 | 1.59E+05 | 1.53E+05 |
| pmb2922 | 5.65E+02 | 3.23E+02 | 1.11E+00 | 5.66E+02 | [M-H]- | C00029 | Uridine 5'-diphospho-D-glucose | Nucleotide and its derivates | 1.07E+06 | 1.14E+06 | 1.30E+06 | 1.54E+06 | 1.61E+06 | 1.50E+06 | 1.80E+06 | 1.32E+06 | 1.56E+06 | 1.06E+06 | 1.31E+06 | 1.19E+06 | 7.89E+05 | 8.11E+05 | 8.00E+05 |
| pmb2928 | 3.31E+02 | 3.14E+02 | 2.02E+00 | 3.32E+02 | [M-H]- | - | Gallic acid O-Hexoside | Benzoic acid derivatives | 8.48E+05 | 7.73E+05 | 7.22E+05 | 7.69E+05 | 7.51E+05 | 6.73E+05 | 7.09E+04 | 6.78E+04 | 6.94E+04 | 3.98E+05 | 2.30E+05 | 3.14E+05 | 1.73E+05 | 6.96E+05 | 4.35E+05 |
| pmb2933 | 3.11E+02 | 1.49E+02 | 2.16E+00 | 3.12E+02 | [M-H]- | - | Caftaric acid | Hydroxycinnamoyl derivatives | 2.39E+05 | 2.12E+05 | 2.35E+05 | 2.14E+05 | 2.09E+05 | 2.25E+05 | 4.38E+05 | 3.54E+05 | 3.96E+05 | 3.43E+05 | 3.32E+05 | 3.38E+05 | 3.48E+05 | 3.96E+05 | 3.72E+05 |
| pmb2936 | 5.91E+02 | 3.67E+02 | 4.95E+00 | 5.92E+02 | [M-H]- | - | Disinapoyl hexoside | Hydroxycinnamoyl derivatives | 2.12E+04 | 2.06E+04 | 2.56E+04 | 2.80E+04 | 2.25E+04 | 2.15E+04 | 5.08E+04 | 4.72E+04 | 4.90E+04 | 1.16E+04 | 1.66E+04 | 1.41E+04 | 4.28E+04 | 2.66E+04 | 3.47E+04 |
| pmb2938 | 3.53E+02 | 1.91E+02 | 3.00E+00 | 3.54E+02 | [M-H]- | - | 4-O-Caffeoyl quinic acid (criptochlorogenic acid) | Quinate and its derivatives | 3.40E+04 | 2.95E+04 | 3.29E+04 | 3.14E+04 | 3.36E+04 | 2.95E+04 | 1.35E+04 | 1.49E+04 | 1.42E+04 | 1.55E+04 | 1.33E+04 | 1.44E+04 | 1.60E+04 | 1.44E+04 | 1.52E+04 |
| pmb2940 | 3.85E+02 | 2.23E+02 | 3.26E+00 | 3.86E+02 | [M-H]- | - | 1-O-beta-D-Glucopyranosyl sinapate | Hydroxycinnamoyl derivatives | 8.61E+06 | 8.63E+06 | 8.62E+06 | 8.31E+06 | 8.40E+06 | 8.31E+06 | 1.69E+07 | 1.70E+07 | 1.70E+07 | 6.63E+06 | 8.65E+06 | 7.64E+06 | 9.38E+06 | 9.64E+06 | 9.51E+06 |
| pmb2952 | 2.51E+02 | 1.35E+02 | 1.63E+00 | 2.52E+02 | [M-H]- | C05512 | 2'-Deoxyinosine | Nucleotide and its derivates | 1.64E+04 | 1.44E+04 | 1.32E+04 | 1.55E+04 | 1.69E+04 | 9.49E+03 | 9.00E+00 | 9.00E+00 | 9.00E+00 | 9.00E+00 | 9.00E+00 | 9.00E+00 | 9.00E+00 | 9.00E+00 | 9.00E+00 |
| pmb2954 | 7.71E+02 | 6.10E+02 | 2.83E+00 | 7.72E+02 | [M-H]- | - | Luteolin O-hexosyl-O-hexosyl-O-hexoside | Flavone | 7.75E+06 | 8.33E+06 | 7.85E+06 | 7.69E+06 | 7.95E+06 | 7.53E+06 | 8.61E+06 | 8.08E+06 | 8.35E+06 | 7.85E+06 | 7.48E+06 | 7.67E+06 | 6.89E+06 | 7.87E+06 | 7.38E+06 |
| pmb2957 | 4.65E+02 | 2.85E+02 | 2.59E+00 | 4.66E+02 | [M-H]- | - | Cyanidin O-syringic acid | Anthocyanins | 1.34E+04 | 1.23E+04 | 1.49E+04 | 1.09E+04 | 1.54E+04 | 1.08E+04 | 2.15E+04 | 2.63E+04 | 2.39E+04 | 2.11E+04 | 8.88E+03 | 1.50E+04 | 9.11E+03 | 1.57E+04 | 1.24E+04 |
| pmb2961 | 5.47E+02 | 5.03E+02 | 3.00E+00 | 5.48E+02 | [M-H]- | - | Peonidin O-malonylhexoside | Anthocyanins | 4.81E+05 | 5.14E+05 | 5.54E+05 | 5.37E+05 | 4.71E+05 | 4.12E+05 | 7.50E+05 | 7.19E+05 | 7.35E+05 | 6.98E+05 | 6.15E+05 | 6.57E+05 | 5.41E+05 | 6.21E+05 | 5.81E+05 |
| pmb2970 | 6.25E+02 | 4.63E+02 | 2.19E+00 | 6.26E+02 | [M-H]- | - | Hesperetin O-hexosyl-O-hexoside | Flavanone | 3.02E+04 | 1.85E+04 | 1.69E+04 | 2.01E+04 | 1.83E+04 | 1.71E+04 | 2.35E+04 | 1.57E+04 | 1.96E+04 | 3.34E+04 | 2.01E+04 | 2.68E+04 | 2.20E+04 | 2.50E+04 | 2.35E+04 |
| pmb2975 | 4.77E+02 | 3.57E+02 | 3.53E+00 | 4.78E+02 | [M-H]- | - | Hesperetin O-Glucuronic acid | Flavanone | 1.36E+04 | 1.56E+04 | 2.06E+04 | 2.02E+04 | 1.29E+04 | 1.37E+04 | 4.17E+04 | 3.86E+04 | 4.02E+04 | 1.59E+04 | 2.21E+04 | 1.90E+04 | 2.07E+04 | 1.52E+04 | 1.80E+04 |
| pmb2979 | 5.49E+02 | 3.87E+02 | 3.91E+00 | 5.50E+02 | [M-H]- | - | Hesperetin O-malonylhexoside | Flavanone | 1.34E+05 | 1.31E+05 | 1.09E+05 | 1.21E+05 | 1.48E+05 | 9.73E+04 | 7.29E+04 | 1.16E+05 | 9.45E+04 | 1.04E+05 | 1.40E+05 | 1.22E+05 | 2.40E+05 | 2.19E+05 | 2.30E+05 |
| pmb2987 | 4.87E+02 | 2.83E+02 | 2.63E+00 | 4.88E+02 | [M-H]- | - | Acacetin O-acetyl hexoside | Flavone | 6.44E+06 | 6.82E+06 | 6.87E+06 | 7.17E+06 | 7.08E+06 | 6.54E+06 | 2.11E+06 | 2.15E+06 | 2.13E+06 | 2.58E+06 | 2.98E+06 | 2.78E+06 | 7.29E+06 | 6.74E+06 | 7.02E+06 |
| pmb2991 | 7.39E+02 | 2.69E+02 | 3.79E+00 | 7.40E+02 | [M-H]- | - | Apigenin O-hexosyl-O-rutinoside | Flavone | 6.94E+03 | 4.23E+03 | 6.15E+03 | 1.08E+04 | 6.60E+03 | 4.93E+03 | 1.32E+04 | 1.12E+04 | 1.22E+04 | 6.97E+03 | 7.79E+03 | 7.38E+03 | 6.01E+03 | 1.02E+04 | 8.11E+03 |
| pmb2999 | 4.61E+02 | 2.85E+02 | 3.87E+00 | 4.62E+02 | [M-H]- | - | Chrysoeriol 5-O-hexoside | Flavone | 2.47E+05 | 2.84E+05 | 2.74E+05 | 2.94E+05 | 3.06E+05 | 3.53E+05 | 2.48E+05 | 2.28E+05 | 2.38E+05 | 1.40E+05 | 1.49E+05 | 1.45E+05 | 2.59E+05 | 3.75E+05 | 3.17E+05 |
| pmb3002 | 6.07E+02 | 2.99E+02 | 3.98E+00 | 6.08E+02 | [M-H]- | - | Chrysoeriol 7-O-rutinoside | Flavone | 1.14E+07 | 1.04E+07 | 1.13E+07 | 1.10E+07 | 1.12E+07 | 1.22E+07 | 9.24E+06 | 9.09E+06 | 9.17E+06 | 1.02E+07 | 7.99E+06 | 9.10E+06 | 9.78E+06 | 1.01E+07 | 9.94E+06 |
| pmb3006 | 4.31E+02 | 2.69E+02 | 4.21E+00 | 4.32E+02 | [M-H]- | C04608 | Apigenin 7-O-glucoside (Cosmosiin) | Flavone | 7.44E+05 | 7.39E+05 | 7.21E+05 | 7.28E+05 | 7.40E+05 | 7.36E+05 | 2.49E+05 | 1.84E+05 | 2.17E+05 | 2.85E+05 | 2.55E+05 | 2.70E+05 | 2.04E+05 | 1.05E+05 | 1.55E+05 |
| pmb3012 | 4.61E+02 | 4.61E+02 | 4.32E+00 | 4.62E+02 | [M-H]- | - | Chrysoeriol 7-O-hexoside | Flavone | 1.07E+05 | 1.11E+05 | 1.08E+05 | 1.23E+05 | 1.09E+05 | 1.12E+05 | 5.78E+04 | 5.47E+04 | 5.63E+04 | 1.34E+05 | 8.24E+04 | 1.08E+05 | 8.91E+04 | 1.02E+05 | 9.56E+04 |
| pmb3023 | 4.49E+02 | 3.29E+02 | 3.37E+00 | 4.50E+02 | [M-H]- | - | Eriodictyol C-hexoside | Flavone C-glycosides | 1.63E+05 | 1.78E+05 | 1.52E+05 | 1.52E+05 | 1.48E+05 | 1.49E+05 | 2.17E+05 | 2.19E+05 | 2.18E+05 | 2.18E+05 | 1.12E+05 | 1.65E+05 | 1.07E+05 | 1.61E+05 | 1.34E+05 |
| pmb3041 | 5.21E+02 | 3.29E+02 | 3.81E+00 | 5.22E+02 | [M-H]- | - | Tricin O-saccharic acid | Flavone | 6.93E+05 | 6.84E+05 | 7.11E+05 | 6.63E+05 | 6.23E+05 | 6.65E+05 | 1.10E+06 | 1.12E+06 | 1.11E+06 | 7.38E+05 | 7.54E+05 | 7.46E+05 | 4.93E+05 | 8.80E+05 | 6.87E+05 |
| pmb3042 | 4.91E+02 | 3.29E+02 | 4.00E+00 | 4.92E+02 | [M-H]- | - | Tricin 5-O-hexoside | Flavone | 5.01E+04 | 5.92E+04 | 6.46E+04 | 4.71E+04 | 5.50E+04 | 5.12E+04 | 3.21E+04 | 3.36E+04 | 3.29E+04 | 1.67E+04 | 3.22E+04 | 2.45E+04 | 9.93E+04 | 8.96E+04 | 9.45E+04 |
| pmb3046 | 4.91E+02 | 4.31E+02 | 4.30E+00 | 4.92E+02 | [M-H]- | - | Tricin 7-O-hexoside | Flavone | 1.19E+05 | 1.29E+05 | 1.45E+05 | 1.38E+05 | 1.28E+05 | 1.22E+05 | 1.55E+05 | 1.32E+05 | 1.44E+05 | 1.34E+05 | 1.20E+05 | 1.27E+05 | 1.55E+05 | 2.97E+05 | 2.26E+05 |
| pmb3049 | 6.57E+02 | 3.29E+02 | 4.72E+00 | 6.58E+02 | [M-H]- | - | Tricin 4'-O-(syringyl alcohol) ether 7-O-hexoside | Flavonolignan | 3.36E+03 | 2.41E+03 | 3.67E+03 | 2.81E+03 | 1.77E+03 | 4.33E+03 | 6.35E+03 | 6.39E+03 | 6.37E+03 | 9.00E+00 | 4.54E+03 | 2.27E+03 | 9.00E+00 | 2.74E+03 | 1.37E+03 |
| pmb3052 | 5.25E+02 | 3.15E+02 | 5.84E+00 | 5.26E+02 | [M-H]- | - | Tricin 4'-O-β-guaiacylglycerol | Flavonolignan | 4.09E+03 | 4.11E+03 | 5.06E+03 | 3.94E+03 | 3.76E+03 | 3.39E+03 | 3.67E+03 | 4.15E+03 | 3.91E+03 | 6.33E+03 | 5.32E+03 | 5.83E+03 | 4.42E+03 | 5.92E+03 | 5.17E+03 |
| pmb3053 | 5.23E+02 | 3.29E+02 | 6.00E+00 | 5.24E+02 | [M-H]- | - | Tricin O-eudesmic acid | Flavone | 5.55E+03 | 5.39E+03 | 4.62E+03 | 5.01E+03 | 5.63E+03 | 5.33E+03 | 7.46E+03 | 6.44E+03 | 6.95E+03 | 3.78E+03 | 3.26E+03 | 3.52E+03 | 4.54E+03 | 1.23E+04 | 8.42E+03 |
| pmb3061 | 4.99E+02 | 1.63E+02 | 2.10E+00 | 5.00E+02 | [M-H]- | - | 5-O-p-coumaroyl quinic acid O-hexoside | Quinate and its derivatives | 3.49E+04 | 4.76E+04 | 4.30E+04 | 5.32E+04 | 5.11E+04 | 4.83E+04 | 2.42E+04 | 1.81E+04 | 2.12E+04 | 5.69E+04 | 3.37E+04 | 4.53E+04 | 4.44E+04 | 3.02E+04 | 3.73E+04 |
| pmb3062 | 3.85E+02 | 1.52E+02 | 2.12E+00 | 3.86E+02 | [M-H]- | - | Eudesmoyl quinic acid | Quinate and its derivatives | 3.07E+04 | 3.50E+04 | 2.84E+04 | 2.90E+04 | 3.83E+04 | 3.49E+04 | 3.63E+04 | 2.55E+04 | 3.09E+04 | 6.61E+04 | 3.83E+04 | 5.22E+04 | 3.92E+04 | 5.39E+04 | 4.66E+04 |
| pmb3064 | 4.99E+02 | 1.73E+02 | 2.50E+00 | 5.00E+02 | [M-H]- | - | 3-O-p-coumaroyl quinic acid O-hexoside | Quinate and its derivatives | 8.62E+04 | 4.57E+04 | 4.48E+04 | 4.67E+04 | 4.72E+04 | 4.36E+04 | 1.10E+04 | 1.98E+04 | 1.54E+04 | 1.38E+04 | 1.68E+04 | 1.53E+04 | 3.47E+04 | 3.87E+04 | 3.67E+04 |
| pmb3066 | 4.81E+02 | 4.45E+02 | 2.62E+00 | 4.82E+02 | [M-H]- | - | 5-O-p-coumaroyl shikimic acid O-hexoside | Quinate and its derivatives | 2.25E+05 | 2.35E+05 | 2.49E+05 | 2.25E+05 | 2.34E+05 | 2.21E+05 | 3.03E+05 | 3.20E+05 | 3.12E+05 | 3.61E+05 | 2.47E+05 | 3.04E+05 | 3.86E+05 | 3.56E+05 | 3.71E+05 |
| pmb3072 | 4.81E+02 | 3.19E+02 | 3.23E+00 | 4.82E+02 | [M-H]- | - | 3-O-p-coumaroyl shikimic acid O-hexoside | Quinate and its derivatives | 5.80E+04 | 6.21E+04 | 5.06E+04 | 5.50E+04 | 5.64E+04 | 4.50E+04 | 3.38E+04 | 3.85E+04 | 3.62E+04 | 3.32E+04 | 3.91E+04 | 3.62E+04 | 9.11E+04 | 9.14E+04 | 9.13E+04 |
| pmb3074 | 3.37E+02 | 1.91E+02 | 3.30E+00 | 3.38E+02 | [M-H]- | - | 3-O-p-Coumaroyl quinic acid | Quinate and its derivatives | 2.33E+06 | 2.20E+06 | 2.34E+06 | 2.28E+06 | 2.25E+06 | 2.23E+06 | 1.82E+06 | 1.65E+06 | 1.74E+06 | 9.16E+05 | 1.25E+06 | 1.08E+06 | 8.73E+05 | 1.39E+06 | 1.13E+06 |
| pmb3075 | 3.19E+02 | 1.45E+02 | 3.93E+00 | 3.20E+02 | [M-H]- | - | 3-O-p-Coumaroyl shikimic acid | Quinate and its derivatives | 2.49E+04 | 2.49E+04 | 1.98E+04 | 1.89E+04 | 2.19E+04 | 2.50E+04 | 1.51E+04 | 1.34E+04 | 1.43E+04 | 1.17E+04 | 1.63E+04 | 1.40E+04 | 1.95E+04 | 1.91E+04 | 1.93E+04 |
| pmb3079 | 3.00E+02 | 7.90E+01 | 1.15E+00 | 3.01E+02 | [M-H]- | C04256 | N-Acetylglucosamine 1-phosphate | Others | 1.88E+04 | 2.98E+04 | 1.80E+04 | 4.59E+04 | 3.61E+04 | 1.36E+04 | 2.47E+04 | 5.00E+04 | 3.74E+04 | 9.00E+00 | 2.63E+04 | 1.32E+04 | 9.00E+00 | 2.92E+04 | 1.46E+04 |
| pmb3081 | 2.89E+02 | 9.70E+01 | 1.08E+00 | 2.90E+02 | [M-H]- | - | Glucarate O-Phosphoric acid | Carbohydrates | 2.18E+06 | 2.59E+06 | 2.78E+06 | 2.46E+06 | 2.55E+06 | 3.14E+06 | 4.12E+06 | 3.37E+06 | 3.75E+06 | 3.30E+06 | 2.31E+06 | 2.81E+06 | 3.11E+06 | 2.04E+06 | 2.58E+06 |
| pmb3088 | 4.21E+02 | 2.41E+02 | 7.10E-01 | 4.22E+02 | [M-H]- | C00689 | Trehalose 6-phosphate | Carbohydrates | 2.49E+05 | 2.37E+05 | 2.46E+05 | 2.19E+05 | 2.29E+05 | 2.37E+05 | 2.30E+05 | 2.10E+05 | 2.20E+05 | 1.96E+05 | 2.10E+05 | 2.03E+05 | 1.78E+05 | 2.13E+05 | 1.96E+05 |
| pmb3094 | 7.03E+02 | 3.51E+02 | 2.35E+00 | 7.04E+02 | [M-H]- | - | Esculetin O-quinacyl esculetin O-quinic acid | Coumarins | 8.23E+03 | 4.80E+03 | 7.62E+03 | 5.55E+03 | 7.09E+03 | 6.78E+03 | 7.95E+03 | 9.04E+03 | 8.50E+03 | 2.26E+03 | 9.23E+03 | 5.75E+03 | 3.16E+04 | 2.11E+04 | 2.64E+04 |
| pmb3099 | 1.53E+02 | 7.89E+01 | 1.56E+00 | 1.54E+02 | [M-H]- | C06608 | Diethyl phosphate | Organic acids | 6.93E+05 | 7.00E+05 | 6.82E+05 | 6.47E+05 | 6.51E+05 | 6.31E+05 | 9.92E+05 | 8.88E+05 | 9.40E+05 | 6.59E+05 | 4.88E+05 | 5.74E+05 | 1.03E+06 | 1.21E+06 | 1.12E+06 |
| pmb3101 | 1.75E+02 | 1.31E+02 | 2.75E+00 | 1.76E+02 | [M-H]- | C02504 | 2-Isopropylmalate | Organic acids | 1.64E+07 | 1.74E+07 | 1.70E+07 | 1.68E+07 | 1.66E+07 | 1.60E+07 | 1.39E+07 | 1.36E+07 | 1.38E+07 | 6.38E+06 | 6.90E+06 | 6.64E+06 | 1.14E+07 | 1.10E+07 | 1.12E+07 |
| pmb3107 | 3.59E+02 | 1.97E+02 | 2.26E+00 | 3.60E+02 | [M-H]- | - | Syringic acid O-glucoside | Benzoic acid derivatives | 1.68E+05 | 1.47E+05 | 1.53E+05 | 1.33E+05 | 1.37E+05 | 1.51E+05 | 1.77E+05 | 1.72E+05 | 1.75E+05 | 1.89E+05 | 1.50E+05 | 1.70E+05 | 1.64E+05 | 1.70E+05 | 1.67E+05 |
| pmb3110 | 1.67E+02 | 1.52E+02 | 3.31E+00 | 1.68E+02 | [M-H]- | C06672 | Vanillic acid | Hydroxycinnamoyl derivatives | 8.89E+04 | 9.36E+04 | 8.61E+04 | 8.44E+04 | 8.79E+04 | 8.28E+04 | 2.17E+05 | 2.05E+05 | 2.11E+05 | 8.97E+04 | 1.34E+05 | 1.12E+05 | 1.12E+05 | 9.46E+04 | 1.03E+05 |
| pmb3116 | 4.74E+02 | 2.77E+02 | 7.72E+00 | 4.75E+02 | [M-H]- | - | LysoPE 18:3 | Lipids_Glycerophospholipids | 1.82E+03 | 1.94E+03 | 2.45E+03 | 2.02E+03 | 1.83E+03 | 2.11E+03 | 9.00E+00 | 9.00E+00 | 9.00E+00 | 1.92E+03 | 5.62E+02 | 1.24E+03 | 2.81E+03 | 5.16E+03 | 3.99E+03 |
| pmb3121 | 4.78E+02 | 2.81E+02 | 9.03E+00 | 4.79E+02 | [M-H]- | - | LysoPE 18:1 | Lipids_Glycerophospholipids | 7.41E+06 | 7.27E+06 | 7.38E+06 | 7.52E+06 | 6.85E+06 | 7.31E+06 | 3.98E+06 | 3.99E+06 | 3.99E+06 | 3.24E+06 | 3.38E+06 | 3.31E+06 | 3.29E+06 | 2.19E+07 | 1.26E+07 |
| pmb3317 | 3.71E+02 | 1.79E+02 | 1.85E+00 | 3.72E+02 | [M-H]- | - | Quinacyl syringic acid | Quinate and its derivatives | 1.73E+04 | 1.90E+04 | 1.39E+04 | 9.01E+03 | 1.14E+04 | 7.24E+03 | 1.42E+04 | 1.76E+04 | 1.59E+04 | 1.65E+04 | 2.02E+04 | 1.84E+04 | 3.97E+04 | 3.06E+04 | 3.52E+04 |
| pmb3374 | 2.89E+02 | 1.19E+02 | 2.23E+00 | 2.90E+02 | [M-H]- | - | N-p-Coumaroyl hydroxydehydroagmatine | Phenolamides | 5.08E+04 | 5.65E+04 | 5.70E+04 | 6.49E+04 | 5.90E+04 | 5.89E+04 | 2.93E+04 | 2.78E+04 | 2.86E+04 | 2.83E+04 | 3.47E+04 | 3.15E+04 | 5.13E+04 | 5.11E+04 | 5.12E+04 |
| pmb3894 | 3.29E+02 | 3.14E+02 | 5.91E+00 | 3.30E+02 | [M-H]- | - | Di-O-methylquercetin | Flavonol | 3.76E+06 | 3.85E+06 | 3.82E+06 | 3.65E+06 | 3.81E+06 | 3.82E+06 | 2.08E+06 | 2.03E+06 | 2.06E+06 | 5.32E+05 | 1.12E+06 | 8.26E+05 | 1.09E+06 | 1.16E+07 | 6.35E+06 |
| pmb5304 | 2.77E+02 | 2.34E+02 | 1.06E+01 | 2.78E+02 | [M-H]- | C06427 | α-Linolenic acid | Lipids_Fatty acids | 7.43E+03 | 6.48E+03 | 9.84E+03 | 8.39E+03 | 9.02E+03 | 1.04E+04 | 1.25E+04 | 1.24E+04 | 1.25E+04 | 1.09E+04 | 9.88E+03 | 1.04E+04 | 7.82E+03 | 1.25E+04 | 1.02E+04 |
| pmc0066 | 3.33E+02 | 1.37E+02 | 7.00E-01 | 3.32E+02 | [M+H]+ | C06196 | 2'-Deoxyinosine-5'-monophosphate | Nucleotide and its derivates | 5.62E+05 | 4.66E+05 | 5.21E+05 | 4.59E+05 | 4.56E+05 | 4.99E+05 | 6.95E+05 | 6.05E+05 | 6.50E+05 | 8.23E+05 | 6.05E+05 | 7.14E+05 | 7.34E+05 | 4.31E+05 | 5.83E+05 |
| pmc0274 | 1.67E+02 | 1.21E+02 | 1.92E+00 | 1.66E+02 | [M+H]+ | C16614 | 6-Methylmercaptopurine | Nucleotide and its derivates | 3.57E+06 | 4.83E+06 | 4.92E+06 | 4.83E+06 | 5.00E+06 | 4.44E+06 | 7.07E+06 | 7.56E+06 | 7.32E+06 | 5.42E+06 | 5.31E+06 | 5.37E+06 | 3.56E+06 | 4.67E+06 | 4.12E+06 |
| pmc0281 | 4.00E+02 | 1.36E+02 | 1.94E+00 | 3.99E+02 | [M+H]+ | - | Adenosine O-ribose | Nucleotide and its derivates | 2.01E+05 | 2.98E+05 | 2.41E+05 | 3.54E+05 | 3.08E+05 | 2.75E+05 | 2.57E+05 | 3.67E+05 | 3.12E+05 | 2.78E+05 | 4.15E+05 | 3.47E+05 | 2.13E+05 | 3.60E+05 | 2.87E+05 |
| pmc0304 | 3.84E+02 | 2.52E+02 | 2.06E+00 | 3.83E+02 | [M+H]+ | - | Succinyladenosine | Nucleotide and its derivates | 6.58E+06 | 8.37E+06 | 8.00E+06 | 8.40E+06 | 7.29E+06 | 7.79E+06 | 9.50E+06 | 1.05E+07 | 1.00E+07 | 8.94E+06 | 8.63E+06 | 8.79E+06 | 7.38E+06 | 8.26E+06 | 7.82E+06 |
| pmc0960 | 5.44E+02 | 5.44E+02 | 8.91E+00 | 5.43E+02 | [M+H]+ | - | LysoPC 20:4 | Lipids_Glycerophospholipids | 2.09E+06 | 1.97E+06 | 2.15E+06 | 2.01E+06 | 1.90E+06 | 2.09E+06 | 6.11E+05 | 6.01E+05 | 6.06E+05 | 1.14E+06 | 9.98E+05 | 1.07E+06 | 2.00E+06 | 3.77E+06 | 2.89E+06 |
| pmc1990 | 2.99E+02 | 7.48E+01 | 6.78E+00 | 3.00E+02 | [M-H]- | - | "4'-Hydroxy-5,7-dimethoxyflavanone" | Flavanone | 1.78E+05 | 1.85E+05 | 1.83E+05 | 1.92E+05 | 1.90E+05 | 2.02E+05 | 6.95E+04 | 1.02E+05 | 8.58E+04 | 7.42E+04 | 9.23E+04 | 8.33E+04 | 6.11E+04 | 8.47E+04 | 7.29E+04 |
| pmd0017 | 2.03E+02 | 1.12E+02 | 6.20E-01 | 2.02E+02 | [M+H]+ | C00750 | Spermine | Phenolamides | 1.14E+06 | 1.70E+06 | 1.95E+06 | 2.34E+06 | 2.91E+06 | 3.18E+06 | 2.79E+06 | 3.04E+06 | 2.92E+06 | 3.35E+06 | 3.48E+06 | 3.42E+06 | 3.07E+06 | 3.34E+06 | 3.21E+06 |
| pmd0023 | 2.66E+02 | 1.34E+02 | 1.81E+00 | 2.67E+02 | [M-H]- | C00212 | Adenosine | Nucleotide and its derivates | 1.01E+06 | 8.66E+05 | 1.00E+06 | 8.55E+05 | 8.85E+05 | 9.82E+05 | 8.15E+05 | 7.42E+05 | 7.79E+05 | 1.09E+06 | 9.04E+05 | 9.97E+05 | 8.07E+05 | 5.92E+05 | 7.00E+05 |
| pmd0130 | 4.68E+02 | 4.51E+02 | 7.73E+00 | 4.67E+02 | [M+H]+ | - | LysoPC 14:0 (2n isomer) | Lipids_Glycerophospholipids | 1.17E+06 | 1.22E+06 | 1.22E+06 | 1.14E+06 | 1.17E+06 | 1.14E+06 | 1.03E+06 | 1.00E+06 | 1.02E+06 | 1.09E+06 | 8.96E+05 | 9.93E+05 | 9.54E+05 | 9.74E+05 | 9.64E+05 |
| pmd0132 | 4.96E+02 | 4.78E+02 | 8.80E+00 | 4.95E+02 | [M+H]+ | - | LysoPC 16:0 (2n isomer) | Lipids_Glycerophospholipids | 3.35E+07 | 3.40E+07 | 3.49E+07 | 3.41E+07 | 3.33E+07 | 3.45E+07 | 3.44E+07 | 3.46E+07 | 3.45E+07 | 4.48E+07 | 3.04E+07 | 3.76E+07 | 4.07E+07 | 6.45E+07 | 5.26E+07 |
| pmd0136 | 5.24E+02 | 5.07E+02 | 9.91E+00 | 5.23E+02 | [M+H]+ | - | LysoPC 18:0 | Lipids_Glycerophospholipids | 1.73E+07 | 1.78E+07 | 1.75E+07 | 1.74E+07 | 1.67E+07 | 1.69E+07 | 3.28E+07 | 3.57E+07 | 3.43E+07 | 2.69E+07 | 1.86E+07 | 2.28E+07 | 2.01E+07 | 2.06E+07 | 2.04E+07 |
| pmd0144 | 5.50E+02 | 2.45E+02 | 9.96E+00 | 5.49E+02 | [M+H]+ | - | LysoPC 20:1 (2n isomer) | Lipids_Glycerophospholipids | 2.20E+06 | 2.30E+06 | 2.25E+06 | 2.22E+06 | 2.13E+06 | 1.44E+06 | 6.89E+05 | 6.38E+05 | 6.64E+05 | 1.40E+06 | 9.24E+05 | 1.16E+06 | 2.96E+06 | 7.99E+06 | 5.48E+06 |
| pmd0145 | 5.50E+02 | 2.45E+02 | 1.00E+01 | 5.49E+02 | [M+H]+ | - | LysoPC 20:1 | Lipids_Glycerophospholipids | 2.15E+06 | 2.29E+06 | 2.25E+06 | 1.48E+06 | 2.09E+06 | 2.07E+06 | 6.36E+05 | 6.33E+05 | 6.35E+05 | 1.40E+06 | 9.47E+05 | 1.17E+06 | 2.57E+06 | 7.90E+06 | 5.24E+06 |
| pmd0158 | 4.24E+02 | 2.86E+02 | 7.51E+00 | 4.25E+02 | [M-H]- | - | LysoPE 14:0 (2n isomer) | Lipids_Glycerophospholipids | 6.78E+03 | 7.36E+03 | 7.62E+03 | 8.11E+03 | 7.67E+03 | 6.79E+03 | 3.70E+03 | 3.73E+03 | 3.72E+03 | 5.25E+03 | 3.68E+03 | 4.47E+03 | 6.12E+03 | 3.35E+04 | 1.98E+04 |
| pmd0160 | 4.52E+02 | 2.55E+02 | 8.73E+00 | 4.53E+02 | [M-H]- | - | LysoPE 16:0 (2n isomer) | Lipids_Glycerophospholipids | 1.10E+07 | 1.11E+07 | 1.13E+07 | 1.13E+07 | 1.10E+07 | 1.10E+07 | 1.42E+07 | 1.72E+07 | 1.57E+07 | 1.36E+07 | 1.06E+07 | 1.21E+07 | 1.17E+07 | 1.28E+07 | 1.23E+07 |
| pme0004 | 1.90E+02 | 8.40E+01 | 8.40E-01 | 1.89E+02 | [M+H]+ | C02427 | L-Homocitrulline | Amino acids | 3.89E+05 | 3.80E+05 | 4.58E+05 | 3.96E+05 | 4.23E+05 | 3.83E+05 | 5.73E+05 | 5.56E+05 | 5.65E+05 | 4.03E+05 | 5.07E+05 | 4.55E+05 | 4.66E+05 | 4.47E+05 | 4.57E+05 |
| pme0005 | 1.21E+02 | 1.21E+02 | 1.44E+00 | 1.20E+02 | [M+H]+ | C15587 | Purine | Nucleotide and its derivates | 2.72E+06 | 3.12E+06 | 3.15E+06 | 3.57E+06 | 3.25E+06 | 3.08E+06 | 2.17E+06 | 2.15E+06 | 2.16E+06 | 1.83E+06 | 2.46E+06 | 2.15E+06 | 5.64E+06 | 6.04E+06 | 5.84E+06 |
| pme0007 | 1.74E+02 | 1.31E+02 | 7.90E-01 | 1.75E+02 | [M-H]- | C00327 | L-Citrulline | Amino acids | 1.90E+06 | 1.90E+06 | 1.58E+06 | 1.75E+06 | 1.80E+06 | 1.63E+06 | 1.47E+06 | 1.38E+06 | 1.43E+06 | 3.39E+05 | 1.46E+06 | 9.00E+05 | 7.14E+05 | 9.01E+05 | 8.08E+05 |
| pme0009 | 1.04E+02 | 7.39E+01 | 7.90E-01 | 1.05E+02 | [M-H]- | C00065 | L-Serine | Amino acids | 1.83E+07 | 1.73E+07 | 1.69E+07 | 1.76E+07 | 1.76E+07 | 1.62E+07 | 1.83E+07 | 1.80E+07 | 1.82E+07 | 2.21E+07 | 1.89E+07 | 2.05E+07 | 2.21E+07 | 2.40E+07 | 2.31E+07 |
| pme0011 | 1.32E+02 | 9.99E+01 | 8.00E-01 | 1.33E+02 | [M-H]- | C00049 | L-Aspartic acid | Amino acids | 1.51E+05 | 1.79E+05 | 1.68E+05 | 2.21E+05 | 1.93E+05 | 1.54E+05 | 1.27E+05 | 1.46E+05 | 1.37E+05 | 1.26E+05 | 2.23E+05 | 1.75E+05 | 1.49E+05 | 1.17E+05 | 1.33E+05 |
| pme0013 | 1.46E+02 | 1.02E+02 | 7.50E-01 | 1.47E+02 | [M-H]- | C00025 | L-Glutamic acid | Amino acids | 1.32E+07 | 1.32E+07 | 1.19E+07 | 1.21E+07 | 1.25E+07 | 1.13E+07 | 1.09E+07 | 1.06E+07 | 1.08E+07 | 1.14E+07 | 1.31E+07 | 1.23E+07 | 1.10E+07 | 1.06E+07 | 1.08E+07 |
| pme0018 | 1.30E+02 | 1.30E+02 | 1.27E+00 | 1.31E+02 | [M-H]- | C00123 | L-Leucine | Amino acids | 2.90E+06 | 2.81E+06 | 2.75E+06 | 2.77E+06 | 2.70E+06 | 2.66E+06 | 3.09E+06 | 2.96E+06 | 3.03E+06 | 1.80E+06 | 2.95E+06 | 2.38E+06 | 3.31E+06 | 3.48E+06 | 3.40E+06 |
| pme0020 | 1.64E+02 | 1.47E+02 | 1.92E+00 | 1.65E+02 | [M-H]- | C00079 | L-Phenylalanine | Amino acids | 3.70E+07 | 3.81E+07 | 3.52E+07 | 3.74E+07 | 3.82E+07 | 3.20E+07 | 5.14E+07 | 5.22E+07 | 5.18E+07 | 3.62E+07 | 3.81E+07 | 3.72E+07 | 2.16E+07 | 3.46E+07 | 2.81E+07 |
| pme0022 | 1.18E+02 | 1.01E+02 | 7.60E-01 | 1.19E+02 | [M-H]- | C00188 | L-Threonine | Amino acids | 1.20E+07 | 1.04E+07 | 9.94E+06 | 1.08E+07 | 1.11E+07 | 9.39E+06 | 1.31E+07 | 1.21E+07 | 1.26E+07 | 1.39E+07 | 1.14E+07 | 1.27E+07 | 1.50E+07 | 1.19E+07 | 1.35E+07 |
| pme0024 | 1.24E+02 | 7.97E+01 | 8.30E-01 | 1.25E+02 | [M-H]- | C00245 | 2-Aminoethanesulfonic acid | Organic acids | 7.73E+04 | 6.07E+04 | 5.76E+04 | 7.90E+04 | 5.61E+04 | 6.23E+04 | 6.66E+04 | 5.38E+04 | 6.02E+04 | 6.17E+04 | 8.15E+04 | 7.16E+04 | 5.46E+04 | 1.18E+05 | 8.63E+04 |
| pme0026 | 1.47E+02 | 8.32E+01 | 7.80E-01 | 1.46E+02 | [M+H]+ | C00047 | L-(+)-Lysine | Amino acids | 4.74E+04 | 6.60E+04 | 5.67E+04 | 8.93E+04 | 1.16E+05 | 1.06E+05 | 7.55E+04 | 6.82E+04 | 7.19E+04 | 5.08E+04 | 9.71E+04 | 7.40E+04 | 1.09E+05 | 1.12E+05 | 1.11E+05 |
| pme0028 | 2.45E+02 | 1.13E+02 | 1.22E+00 | 2.44E+02 | [M+H]+ | C00299 | Uridine | Nucleotide and its derivates | 8.02E+06 | 9.04E+06 | 9.85E+06 | 9.89E+06 | 9.95E+06 | 8.81E+06 | 2.17E+07 | 2.24E+07 | 2.21E+07 | 1.01E+07 | 1.41E+07 | 1.21E+07 | 7.41E+06 | 6.73E+06 | 7.07E+06 |
| pme0030 | 1.82E+02 | 1.36E+02 | 1.16E+00 | 1.81E+02 | [M+H]+ | C00082 | L-(-)-Tyrosine | Amino acids | 6.21E+06 | 8.76E+06 | 8.43E+06 | 8.21E+06 | 8.99E+06 | 8.03E+06 | 1.22E+07 | 1.11E+07 | 1.17E+07 | 6.66E+06 | 8.61E+06 | 7.64E+06 | 9.23E+06 | 8.82E+06 | 9.03E+06 |
| pme0031 | 1.27E+02 | 1.10E+02 | 1.59E+00 | 1.26E+02 | [M+H]+ | C00178 | Thymine | Nucleotide and its derivates | 6.29E+04 | 9.46E+04 | 7.63E+04 | 1.29E+05 | 5.77E+04 | 7.26E+04 | 6.53E+04 | 9.01E+04 | 7.77E+04 | 7.35E+04 | 9.70E+04 | 8.53E+04 | 4.84E+04 | 8.19E+04 | 6.52E+04 |
| pme0033 | 1.37E+02 | 1.10E+02 | 1.16E+00 | 1.36E+02 | [M+H]+ | C00262 | Hypoxanthine | Nucleotide and its derivates | 1.35E+06 | 1.58E+06 | 1.40E+06 | 1.45E+06 | 1.48E+06 | 1.34E+06 | 2.36E+06 | 2.20E+06 | 2.28E+06 | 1.12E+06 | 1.71E+06 | 1.42E+06 | 1.42E+06 | 1.39E+06 | 1.41E+06 |
| pme0036 | 1.54E+02 | 9.29E+01 | 7.20E-01 | 1.55E+02 | [M-H]- | C00135 | L-Histidine | Amino acids | 5.90E+06 | 6.01E+06 | 5.56E+06 | 5.89E+06 | 5.71E+06 | 5.41E+06 | 4.11E+06 | 4.02E+06 | 4.07E+06 | 3.02E+06 | 4.86E+06 | 3.94E+06 | 5.18E+06 | 5.91E+06 | 5.55E+06 |
| pme0038 | 1.12E+02 | 9.50E+01 | 8.20E-01 | 1.11E+02 | [M+H]+ | C00380 | Cytosine | Nucleotide and its derivates | 2.36E+06 | 2.76E+06 | 4.47E+06 | 4.25E+06 | 3.04E+06 | 4.41E+06 | 6.72E+06 | 4.93E+06 | 5.83E+06 | 2.92E+06 | 4.87E+06 | 3.90E+06 | 4.04E+06 | 2.64E+06 | 3.34E+06 |
| pme0039 | 1.18E+02 | 7.18E+01 | 8.50E-01 | 1.17E+02 | [M+H]+ | C00183 | L-Valine | Amino acids | 4.00E+07 | 6.28E+07 | 6.03E+07 | 6.14E+07 | 6.06E+07 | 5.67E+07 | 5.01E+07 | 4.64E+07 | 4.83E+07 | 4.07E+07 | 5.37E+07 | 4.72E+07 | 6.85E+07 | 7.33E+07 | 7.09E+07 |
| pme0042 | 1.73E+02 | 1.31E+02 | 7.30E-01 | 1.74E+02 | [M-H]- | C00062 | L-(+)-Arginine | Amino acids | 2.72E+07 | 2.55E+07 | 2.47E+07 | 2.50E+07 | 2.49E+07 | 2.48E+07 | 2.34E+07 | 2.29E+07 | 2.32E+07 | 1.29E+07 | 2.44E+07 | 1.87E+07 | 2.19E+07 | 1.88E+07 | 2.04E+07 |
| pme0048 | 1.08E+02 | 6.37E+01 | 7.60E-01 | 1.09E+02 | [M-H]- | C00519 | 2-Aminoethanesulfinic acid | Organic acids | 2.23E+04 | 2.34E+04 | 1.93E+04 | 2.35E+04 | 2.16E+04 | 1.19E+04 | 5.78E+03 | 3.19E+03 | 4.49E+03 | 2.31E+04 | 2.41E+04 | 2.36E+04 | 3.35E+04 | 3.38E+04 | 3.37E+04 |
| pme0050 | 2.03E+02 | 1.16E+02 | 2.39E+00 | 2.04E+02 | [M-H]- | C00078 | L-Tryptophan | Amino acids | 1.83E+07 | 1.92E+07 | 1.89E+07 | 1.90E+07 | 1.94E+07 | 1.72E+07 | 2.02E+07 | 2.13E+07 | 2.08E+07 | 2.28E+07 | 2.11E+07 | 2.20E+07 | 2.77E+07 | 2.52E+07 | 2.65E+07 |
| pme0056 | 1.45E+02 | 1.01E+02 | 2.57E+00 | 1.46E+02 | [M-H]- | - | "2,3-dimethylsuccinic acid" | Amino acid derivatives | 2.80E+05 | 2.83E+05 | 2.89E+05 | 2.87E+05 | 2.84E+05 | 2.95E+05 | 3.48E+05 | 3.25E+05 | 3.37E+05 | 3.05E+05 | 2.64E+05 | 2.85E+05 | 2.60E+05 | 2.86E+05 | 2.73E+05 |
| pme0063 | 5.17E+02 | 4.81E+02 | 5.88E+00 | 5.16E+02 | [M+H]+ | C08796 | Cucurbitacin D | Terpenoids | 4.48E+05 | 4.22E+05 | 4.14E+05 | 4.21E+05 | 4.56E+05 | 4.37E+05 | 1.99E+05 | 2.50E+05 | 2.25E+05 | 1.38E+05 | 4.35E+05 | 2.87E+05 | 3.08E+05 | 2.70E+05 | 2.89E+05 |
| pme0075 | 1.88E+02 | 1.02E+02 | 1.18E+00 | 1.89E+02 | [M-H]- | C00624 | N-Acetyl-L-glutamic acid | Amino acid derivatives | 5.80E+05 | 5.97E+05 | 5.21E+05 | 6.36E+05 | 6.42E+05 | 5.44E+05 | 2.59E+05 | 2.50E+05 | 2.55E+05 | 1.16E+05 | 2.49E+05 | 1.83E+05 | 1.29E+05 | 1.77E+05 | 1.53E+05 |
| pme0085 | 3.59E+02 | 1.61E+02 | 3.87E+00 | 3.60E+02 | [M-H]- | C01850 | Rosmarinic acid | Organic acids | 1.65E+05 | 1.73E+05 | 1.54E+05 | 1.63E+05 | 1.63E+05 | 1.61E+05 | 2.41E+05 | 2.31E+05 | 2.36E+05 | 1.09E+05 | 1.05E+05 | 1.07E+05 | 6.89E+04 | 1.53E+05 | 1.11E+05 |
| pme0089 | 2.87E+02 | 2.87E+02 | 5.00E+00 | 2.86E+02 | [M+H]+ | C01514 | Luteolin | Flavone | 1.18E+04 | 1.39E+04 | 2.06E+04 | 2.84E+04 | 2.06E+04 | 2.34E+04 | 2.68E+04 | 2.29E+04 | 2.49E+04 | 1.41E+04 | 1.27E+04 | 1.34E+04 | 2.53E+04 | 3.21E+04 | 2.87E+04 |
| pme0108 | 1.26E+02 | 1.26E+02 | 1.16E+00 | 1.25E+02 | [M+H]+ | C02376 | 5-Methylcytosine | Nucleotide and its derivates | 1.26E+05 | 1.43E+05 | 1.64E+05 | 1.58E+05 | 1.25E+05 | 1.56E+05 | 1.42E+05 | 1.24E+05 | 1.33E+05 | 1.29E+05 | 1.32E+05 | 1.31E+05 | 1.32E+05 | 1.14E+05 | 1.23E+05 |
| pme0109 | 2.12E+02 | 1.66E+02 | 1.45E+00 | 2.11E+02 | [M+H]+ | C07194 | "(-)-3-(3,4-Dihydroxyphenyl)-2-methylalanine" | Amino acid derivatives | 2.33E+06 | 2.71E+06 | 2.65E+06 | 2.94E+06 | 2.65E+06 | 2.29E+06 | 2.33E+06 | 2.35E+06 | 2.34E+06 | 1.87E+06 | 2.39E+06 | 2.13E+06 | 2.05E+06 | 2.60E+06 | 2.33E+06 |
| pme0118 | 1.10E+02 | 6.59E+01 | 3.00E+00 | 1.11E+02 | [M-H]- | C05942 | Pyrrole-2-carboxylic acid | Amino acid derivatives | 1.77E+04 | 1.81E+04 | 2.01E+04 | 1.56E+04 | 1.87E+04 | 1.92E+04 | 2.87E+04 | 3.24E+04 | 3.06E+04 | 2.40E+04 | 2.90E+04 | 2.65E+04 | 2.17E+04 | 2.76E+04 | 2.47E+04 |
| pme0120 | 1.18E+02 | 1.01E+02 | 8.40E-01 | 1.17E+02 | [M+H]+ | C00431 | 5-Aminovaleric acid | Amino acid derivatives | 3.66E+04 | 1.76E+04 | 3.19E+04 | 4.28E+04 | 2.07E+04 | 3.85E+04 | 5.27E+04 | 4.52E+04 | 4.90E+04 | 2.92E+04 | 4.19E+04 | 3.56E+04 | 3.65E+04 | 4.29E+04 | 3.97E+04 |
| pme0122 | 1.89E+02 | 8.40E+01 | 1.14E+00 | 1.88E+02 | [M+H]+ | C02727 | N6-Acetyl-L-lysine | Amino acid derivatives | 2.64E+06 | 2.75E+06 | 3.30E+06 | 3.31E+06 | 2.90E+06 | 3.09E+06 | 4.15E+06 | 3.78E+06 | 3.97E+06 | 3.31E+06 | 4.66E+06 | 3.99E+06 | 4.71E+06 | 3.35E+06 | 4.03E+06 |
| pme0128 | 1.59E+02 | 8.80E+01 | 7.50E-01 | 1.60E+02 | [M-H]- | C00993 | D-Alanyl-D-Alanine | Amino acid derivatives | 1.17E+04 | 8.73E+03 | 1.02E+04 | 9.48E+03 | 1.26E+04 | 9.20E+03 | 1.31E+04 | 1.31E+04 | 1.31E+04 | 1.40E+04 | 1.25E+04 | 1.33E+04 | 1.45E+04 | 1.58E+04 | 1.52E+04 |
| pme0132 | 2.79E+02 | 1.47E+02 | 2.37E+00 | 2.80E+02 | [M-H]- | - | Asp-phe | Amino acid derivatives | 1.08E+05 | 1.21E+05 | 1.17E+05 | 1.16E+05 | 1.19E+05 | 1.11E+05 | 1.61E+05 | 1.60E+05 | 1.61E+05 | 9.08E+04 | 1.23E+05 | 1.07E+05 | 1.38E+05 | 1.23E+05 | 1.31E+05 |
| pme0137 | 1.87E+02 | 1.27E+02 | 1.11E+00 | 1.88E+02 | [M-H]- | - | Nα-Acetyl-L-glutamine | Amino acid derivatives | 9.68E+05 | 1.07E+06 | 9.29E+05 | 1.08E+06 | 1.02E+06 | 8.66E+05 | 1.36E+06 | 1.37E+06 | 1.37E+06 | 1.09E+06 | 1.43E+06 | 1.26E+06 | 1.09E+06 | 1.05E+06 | 1.07E+06 |
| pme0151 | 1.15E+02 | 1.15E+02 | 1.27E+00 | 1.14E+02 | [M+H]+ | C00429 | Dihydrouracil | Nucleotide and its derivates | 1.58E+04 | 1.89E+04 | 3.20E+04 | 2.48E+04 | 1.80E+04 | 1.76E+04 | 4.82E+04 | 4.45E+04 | 4.64E+04 | 2.35E+04 | 3.85E+04 | 3.10E+04 | 3.51E+04 | 2.56E+04 | 3.04E+04 |
| pme0161 | 1.18E+02 | 1.01E+02 | 7.50E-01 | 1.19E+02 | [M-H]- | C00263 | L-Homoserine | Amino acids | 2.37E+05 | 2.62E+05 | 2.10E+05 | 2.30E+05 | 2.47E+05 | 2.18E+05 | 2.66E+05 | 2.43E+05 | 2.55E+05 | 3.29E+05 | 2.49E+05 | 2.89E+05 | 3.31E+05 | 3.03E+05 | 3.17E+05 |
| pme0163 | 3.35E+02 | 1.23E+02 | 8.00E-01 | 3.34E+02 | [M+H]+ | C00455 | β-Nicotinamide mononucleotide | Nucleotide and its derivates | 1.84E+06 | 1.97E+06 | 2.27E+06 | 1.97E+06 | 2.15E+06 | 1.72E+06 | 1.10E+06 | 9.42E+05 | 1.02E+06 | 1.03E+06 | 9.63E+05 | 9.97E+05 | 8.12E+05 | 6.82E+05 | 7.47E+05 |
| pme0164 | 2.63E+02 | 1.89E+02 | 3.90E+00 | 2.64E+02 | [M-H]- | C05642 | N-γ-Acetyl-N-2-Formyl-5-methoxykynurenamine | Amino acid derivatives | 2.27E+04 | 2.09E+04 | 2.22E+04 | 2.36E+04 | 2.37E+04 | 3.42E+04 | 1.43E+04 | 1.18E+04 | 1.31E+04 | 2.23E+04 | 1.81E+04 | 2.02E+04 | 1.67E+04 | 2.30E+04 | 1.99E+04 |
| pme0170 | 2.17E+02 | 1.58E+02 | 1.14E+00 | 2.16E+02 | [M+H]+ | - | Nα-Acetyl-L-arginine | Amino acid derivatives | 1.18E+05 | 1.90E+05 | 1.79E+05 | 2.30E+05 | 1.99E+05 | 1.93E+05 | 2.93E+05 | 2.22E+05 | 2.58E+05 | 1.89E+05 | 1.88E+05 | 1.89E+05 | 1.44E+05 | 9.95E+04 | 1.22E+05 |
| pme0174 | 1.32E+02 | 7.28E+01 | 1.29E+00 | 1.31E+02 | [M+H]+ | - | N-Propionylglycine | Amino acid derivatives | 1.60E+06 | 1.88E+06 | 2.28E+06 | 2.39E+06 | 2.41E+06 | 1.71E+06 | 2.20E+06 | 2.33E+06 | 2.27E+06 | 1.86E+06 | 2.35E+06 | 2.11E+06 | 2.34E+06 | 2.38E+06 | 2.36E+06 |
| pme0180 | 1.68E+02 | 1.51E+02 | 7.20E-01 | 1.69E+02 | [M-H]- | C01152 | 1-Methylhistidine | Amino acid derivatives | 1.70E+04 | 1.69E+04 | 1.69E+04 | 1.81E+04 | 2.06E+04 | 1.59E+04 | 1.75E+04 | 1.71E+04 | 1.73E+04 | 1.34E+04 | 1.66E+04 | 1.50E+04 | 1.66E+04 | 1.84E+04 | 1.75E+04 |
| pme0190 | 1.87E+02 | 1.30E+02 | 1.91E+00 | 1.88E+02 | [M-H]- | C02155 | N-Glycyl-L-leucine | Amino acid derivatives | 8.79E+04 | 8.32E+04 | 7.94E+04 | 9.84E+04 | 8.73E+04 | 7.66E+04 | 7.92E+04 | 1.03E+05 | 9.11E+04 | 6.17E+04 | 7.80E+04 | 6.99E+04 | 7.07E+04 | 9.60E+04 | 8.34E+04 |
| pme0197 | 6.09E+02 | 6.09E+02 | 3.70E+00 | 6.10E+02 | [M-H]- | C05625 | Quercetin 3-O-rutinoside (Rutin) | Flavonol | 5.33E+06 | 5.34E+06 | 5.09E+06 | 5.06E+06 | 4.95E+06 | 4.70E+06 | 4.73E+06 | 4.61E+06 | 4.67E+06 | 4.77E+06 | 3.84E+06 | 4.31E+06 | 5.15E+06 | 4.92E+06 | 5.04E+06 |
| pme0199 | 3.01E+02 | 1.51E+02 | 5.12E+00 | 3.02E+02 | [M-H]- | C00389 | Quercetin | Flavonol | 5.02E+04 | 3.21E+04 | 3.45E+04 | 2.47E+04 | 2.72E+04 | 3.39E+04 | 3.64E+04 | 4.05E+04 | 3.85E+04 | 2.11E+04 | 2.32E+04 | 2.22E+04 | 2.76E+04 | 3.03E+04 | 2.90E+04 |
| pme0200 | 2.87E+02 | 2.87E+02 | 5.73E+00 | 2.86E+02 | [M+H]+ | C05903 | Kaempferol | Flavonol | 1.01E+04 | 1.33E+04 | 1.80E+04 | 1.61E+04 | 9.00E+00 | 1.16E+04 | 2.03E+04 | 2.25E+04 | 2.14E+04 | 1.18E+04 | 1.49E+04 | 1.34E+04 | 8.33E+03 | 1.77E+04 | 1.30E+04 |
| pme0207 | 1.03E+02 | 1.03E+02 | 1.29E+00 | 1.04E+02 | [M-H]- | - | 3-Hydroxybutyrate | Organic acids | 5.81E+05 | 5.50E+05 | 5.48E+05 | 5.13E+05 | 5.86E+05 | 5.05E+05 | 1.55E+06 | 1.64E+06 | 1.60E+06 | 3.66E+05 | 1.25E+06 | 8.08E+05 | 6.30E+05 | 8.56E+05 | 7.43E+05 |
| pme0226 | 1.31E+02 | 7.18E+01 | 7.80E-01 | 1.32E+02 | [M-H]- | C00152 | L-Asparagine | Amino acids | 2.11E+07 | 2.05E+07 | 1.98E+07 | 2.07E+07 | 2.03E+07 | 1.82E+07 | 1.71E+07 | 1.69E+07 | 1.70E+07 | 1.08E+07 | 1.86E+07 | 1.47E+07 | 1.71E+07 | 1.67E+07 | 1.69E+07 |
| pme0231 | 2.47E+02 | 1.30E+02 | 4.07E+00 | 2.46E+02 | [M+H]+ | - | N-(3-Indolylacetyl)-L-alanine | Amino acid derivatives | 5.28E+05 | 5.00E+05 | 5.57E+05 | 5.14E+05 | 4.18E+05 | 4.38E+05 | 4.65E+05 | 4.65E+05 | 4.65E+05 | 3.75E+05 | 3.70E+05 | 3.73E+05 | 4.04E+05 | 4.78E+05 | 4.41E+05 |
| pme0233 | 1.88E+02 | 1.44E+02 | 2.64E+00 | 1.89E+02 | [M-H]- | C01717 | Kynurenic acid | Organic acids | 2.11E+05 | 2.26E+05 | 2.13E+05 | 2.16E+05 | 2.31E+05 | 2.18E+05 | 4.31E+05 | 4.19E+05 | 4.25E+05 | 2.75E+05 | 3.48E+05 | 3.12E+05 | 2.28E+05 | 3.10E+05 | 2.69E+05 |
| pme0237 | 1.83E+02 | 1.37E+02 | 4.77E+00 | 1.82E+02 | [M+H]+ | - | "Ethyl 3,4-Dihydroxybenzoate (Ethyl protocatechuate)" | Organic acids | 5.38E+04 | 5.73E+04 | 7.23E+04 | 6.26E+04 | 4.39E+04 | 5.02E+04 | 6.98E+04 | 6.91E+04 | 6.95E+04 | 4.45E+04 | 5.44E+04 | 4.95E+04 | 6.77E+04 | 6.58E+04 | 6.68E+04 |
| pme0240 | 1.21E+02 | 7.70E+01 | 4.57E+00 | 1.22E+02 | [M-H]- | C00180 | Benzoic acid | Benzoic acid derivatives | 6.30E+05 | 6.69E+05 | 6.76E+05 | 7.61E+05 | 7.53E+05 | 7.69E+05 | 4.05E+05 | 4.56E+05 | 4.31E+05 | 3.61E+05 | 5.08E+05 | 4.35E+05 | 3.48E+05 | 7.24E+05 | 5.36E+05 |
| pme0243 | 1.31E+02 | 8.69E+01 | 1.86E+00 | 1.32E+02 | [M-H]- | C00489 | Glutaric acid | Organic acids | 4.02E+05 | 3.55E+05 | 3.70E+05 | 3.40E+05 | 3.75E+05 | 3.55E+05 | 5.13E+05 | 4.28E+05 | 4.71E+05 | 2.87E+05 | 3.86E+05 | 3.37E+05 | 4.99E+05 | 6.48E+05 | 5.74E+05 |
| pme0245 | 1.45E+02 | 8.09E+01 | 2.58E+00 | 1.46E+02 | [M-H]- | C06104 | Adipic acid | Organic acids | 3.11E+05 | 3.56E+05 | 3.42E+05 | 3.62E+05 | 3.61E+05 | 3.26E+05 | 4.01E+05 | 4.11E+05 | 4.06E+05 | 3.42E+05 | 2.83E+05 | 3.13E+05 | 2.96E+05 | 3.32E+05 | 3.14E+05 |
| pme0246 | 1.22E+02 | 8.01E+01 | 7.70E-01 | 1.21E+02 | [M+H]+ | C00097 | L-Cysteine | Amino acids | 1.34E+04 | 2.49E+04 | 3.17E+04 | 3.23E+04 | 2.96E+04 | 3.73E+04 | 2.98E+04 | 4.05E+04 | 3.52E+04 | 3.66E+04 | 3.00E+04 | 3.33E+04 | 3.45E+04 | 4.52E+04 | 3.99E+04 |
| pme0250 | 1.87E+02 | 1.25E+02 | 4.47E+00 | 1.88E+02 | [M-H]- | C08261 | Azelaic Acid | Organic acids | 2.88E+06 | 2.80E+06 | 2.78E+06 | 2.79E+06 | 2.75E+06 | 2.70E+06 | 3.31E+06 | 2.95E+06 | 3.13E+06 | 1.77E+06 | 3.13E+06 | 2.45E+06 | 4.96E+06 | 9.89E+06 | 7.43E+06 |
| pme0252 | 1.72E+02 | 1.30E+02 | 3.51E+00 | 1.73E+02 | [M-H]- | C02710 | N-Acetyl-l-leucine | Amino acid derivatives | 5.61E+06 | 5.74E+06 | 5.68E+06 | 5.49E+06 | 5.28E+06 | 5.19E+06 | 6.41E+06 | 6.26E+06 | 6.34E+06 | 5.46E+06 | 5.12E+06 | 5.29E+06 | 4.82E+06 | 5.41E+06 | 5.12E+06 |
| pme0258 | 1.13E+02 | 9.59E+01 | 1.23E+00 | 1.12E+02 | [M+H]+ | C00106 | Uracil | Nucleotide and its derivates | 3.51E+06 | 4.59E+06 | 4.76E+06 | 5.05E+06 | 4.76E+06 | 4.64E+06 | 1.09E+07 | 1.08E+07 | 1.09E+07 | 4.96E+06 | 7.31E+06 | 6.14E+06 | 3.93E+06 | 3.75E+06 | 3.84E+06 |
| pme0265 | 2.03E+02 | 9.30E+01 | 5.06E+00 | 2.02E+02 | [M+H]+ | C08277 | Sebacate | Organic acids | 1.50E+04 | 2.45E+04 | 2.39E+04 | 2.50E+04 | 1.67E+04 | 1.69E+04 | 1.44E+04 | 2.13E+04 | 1.79E+04 | 7.37E+03 | 8.79E+03 | 8.08E+03 | 1.87E+04 | 2.13E+04 | 2.00E+04 |
| pme0267 | 1.31E+02 | 8.69E+01 | 2.12E+00 | 1.32E+02 | [M-H]- | - | 2-Methylsuccinic acid | Organic acids | 3.27E+06 | 3.20E+06 | 3.31E+06 | 3.03E+06 | 3.21E+06 | 3.18E+06 | 2.85E+06 | 2.40E+06 | 2.63E+06 | 3.43E+06 | 2.61E+06 | 3.02E+06 | 2.86E+06 | 2.97E+06 | 2.92E+06 |
| pme0270 | 1.17E+02 | 9.89E+01 | 1.33E+00 | 1.16E+02 | [M+H]+ | C01384 | Maleic acid | Organic acids | 9.20E+06 | 1.24E+07 | 1.24E+07 | 1.50E+07 | 1.34E+07 | 1.56E+06 | 1.12E+07 | 1.08E+07 | 1.10E+07 | 8.38E+06 | 1.10E+07 | 9.69E+06 | 1.76E+07 | 1.45E+07 | 1.61E+07 |
| pme0274 | 1.32E+02 | 7.69E+01 | 1.20E+00 | 1.31E+02 | [M+H]+ | C02378 | 6-Aminocaproic acid | Organic acids | 1.08E+06 | 1.33E+06 | 1.27E+06 | 1.24E+06 | 1.31E+06 | 1.16E+06 | 1.20E+06 | 1.20E+06 | 1.20E+06 | 9.44E+05 | 1.34E+06 | 1.14E+06 | 1.38E+06 | 1.38E+06 | 1.38E+06 |
| pme0275 | 1.15E+02 | 9.79E+01 | 1.93E+00 | 1.16E+02 | [M-H]- | - | 4-Oxopentanoate | Organic acids | 2.18E+04 | 1.67E+04 | 1.46E+04 | 1.99E+04 | 1.86E+04 | 1.10E+04 | 3.05E+04 | 2.93E+04 | 2.99E+04 | 1.10E+04 | 1.63E+04 | 1.37E+04 | 2.66E+04 | 2.45E+04 | 2.56E+04 |
| pme0279 | 1.89E+02 | 1.28E+02 | 7.90E-01 | 1.90E+02 | [M-H]- | C00666 | "2,6-Diaminooimelic acid" | Amino acid derivatives | 6.02E+05 | 5.24E+05 | 5.05E+05 | 5.62E+05 | 5.72E+05 | 5.28E+05 | 5.53E+05 | 5.48E+05 | 5.51E+05 | 6.09E+05 | 5.73E+05 | 5.91E+05 | 6.37E+05 | 7.05E+05 | 6.71E+05 |
| pme0281 | 1.65E+02 | 1.21E+02 | 2.96E+00 | 1.66E+02 | [M-H]- | C06337 | Terephthalic acid | Organic acids | 2.99E+06 | 2.99E+06 | 2.96E+06 | 3.00E+06 | 2.95E+06 | 2.86E+06 | 4.13E+06 | 3.91E+06 | 4.02E+06 | 3.77E+06 | 3.04E+06 | 3.41E+06 | 3.35E+06 | 3.35E+06 | 3.35E+06 |
| pme0282 | 1.65E+02 | 7.69E+01 | 2.95E+00 | 1.66E+02 | [M-H]- | C01606 | Phthalic acid | Organic acids | 2.76E+05 | 2.96E+05 | 2.86E+05 | 3.02E+05 | 2.80E+05 | 2.88E+05 | 4.17E+05 | 3.97E+05 | 4.07E+05 | 3.74E+05 | 2.86E+05 | 3.30E+05 | 3.09E+05 | 3.04E+05 | 3.07E+05 |
| pme0285 | 1.46E+02 | 8.59E+01 | 8.40E-01 | 1.45E+02 | [M+H]+ | C01035 | 4-Guanidinobutyric acid | Organic acids | 6.33E+05 | 7.94E+05 | 7.66E+05 | 1.10E+06 | 8.53E+05 | 1.06E+06 | 1.24E+06 | 1.27E+06 | 1.26E+06 | 7.06E+05 | 8.01E+05 | 7.54E+05 | 5.04E+05 | 6.88E+05 | 5.96E+05 |
| pme0289 | 1.51E+02 | 7.88E+01 | 5.93E+00 | 1.50E+02 | [M+H]+ | - | 4-Ethylbenzoic acid | Organic acids | 5.40E+04 | 4.44E+04 | 4.87E+04 | 4.69E+04 | 4.56E+04 | 4.37E+04 | 5.59E+04 | 4.56E+04 | 5.08E+04 | 7.21E+04 | 5.74E+04 | 6.48E+04 | 7.49E+04 | 4.74E+04 | 6.12E+04 |
| pme0291 | 1.60E+02 | 1.14E+02 | 3.13E+00 | 1.59E+02 | [M+H]+ | - | Dl-2-Aminooctanoic acid | Organic acids | 2.57E+04 | 5.10E+04 | 3.96E+04 | 4.79E+04 | 4.82E+04 | 4.27E+04 | 2.83E+04 | 4.27E+04 | 3.55E+04 | 4.83E+04 | 5.36E+04 | 5.10E+04 | 7.61E+04 | 4.11E+04 | 5.86E+04 |
| pme0292 | 1.95E+02 | 1.21E+02 | 3.95E+00 | 1.96E+02 | [M-H]- | - | "(3,4-Dimethoxyphenyl) acetic acid" | Organic acids | 5.89E+04 | 7.28E+04 | 5.97E+04 | 5.92E+04 | 5.16E+04 | 4.65E+04 | 1.04E+05 | 9.29E+04 | 9.85E+04 | 6.63E+04 | 7.18E+04 | 6.91E+04 | 4.96E+04 | 4.25E+04 | 4.61E+04 |
| pme0299 | 1.47E+02 | 6.18E+01 | 5.45E+00 | 1.48E+02 | [M-H]- | C00423 | Cinnamic acid | Hydroxycinnamoyl derivatives | 3.23E+04 | 3.57E+04 | 3.35E+04 | 3.14E+04 | 3.22E+04 | 3.31E+04 | 1.97E+04 | 1.97E+04 | 1.97E+04 | 6.04E+03 | 1.99E+04 | 1.30E+04 | 2.47E+04 | 4.04E+04 | 3.26E+04 |
| pme0303 | 1.79E+02 | 1.35E+02 | 3.23E+00 | 1.80E+02 | [M-H]- | C01197 | Caffeic acid | Hydroxycinnamoyl derivatives | 6.47E+05 | 6.11E+05 | 6.02E+05 | 5.40E+05 | 5.47E+05 | 5.50E+05 | 4.81E+05 | 4.84E+05 | 4.83E+05 | 1.11E+06 | 6.22E+05 | 8.66E+05 | 7.41E+05 | 7.21E+05 | 7.31E+05 |
| pme0305 | 1.93E+02 | 1.34E+02 | 4.07E+00 | 1.94E+02 | [M-H]- | C01494 | Ferulic acid | Hydroxycinnamoyl derivatives | 1.00E+07 | 1.02E+07 | 1.01E+07 | 9.62E+06 | 9.47E+06 | 9.27E+06 | 1.61E+07 | 1.57E+07 | 1.59E+07 | 9.18E+06 | 1.34E+07 | 1.13E+07 | 8.90E+06 | 8.37E+06 | 8.64E+06 |
| pme0307 | 2.27E+02 | 1.43E+02 | 4.78E+00 | 2.28E+02 | [M-H]- | C03582 | Resveratrol | Hydroxycinnamoyl derivatives | 9.10E+03 | 1.10E+04 | 1.19E+04 | 1.06E+04 | 8.79E+03 | 1.35E+04 | 2.79E+03 | 1.47E+03 | 2.13E+03 | 2.74E+03 | 3.90E+03 | 3.32E+03 | 2.24E+03 | 3.69E+03 | 2.97E+03 |
| pme0309 | 1.83E+02 | 1.24E+02 | 3.14E+00 | 1.84E+02 | [M-H]- | - | Methyl gallate | Benzoic acid derivatives | 2.95E+04 | 2.57E+04 | 2.70E+04 | 2.84E+04 | 2.71E+04 | 2.88E+04 | 1.36E+04 | 1.47E+04 | 1.42E+04 | 1.19E+04 | 1.50E+04 | 1.35E+04 | 1.60E+04 | 1.66E+04 | 1.63E+04 |
| pme0330 | 5.79E+02 | 1.35E+02 | 4.17E+00 | 5.80E+02 | [M-H]- | C09789 | Naringenin 7-O-neohesperidoside (Naringin) | Flavanone | 4.62E+04 | 4.29E+04 | 3.91E+04 | 3.59E+04 | 4.85E+04 | 3.26E+04 | 3.17E+04 | 3.21E+04 | 3.19E+04 | 1.37E+04 | 1.50E+04 | 1.44E+04 | 9.90E+03 | 1.11E+04 | 1.05E+04 |
| pme0332 | 5.77E+02 | 2.69E+02 | 4.01E+00 | 5.78E+02 | [M-H]- | C12627 | Apigenin 7-O-neohesperidoside (Rhoifolin) | Flavone | 2.65E+04 | 2.13E+04 | 2.57E+04 | 2.54E+04 | 2.08E+04 | 2.79E+04 | 3.32E+04 | 3.69E+04 | 3.51E+04 | 2.32E+04 | 2.91E+04 | 2.62E+04 | 2.74E+04 | 5.92E+04 | 4.33E+04 |
| pme0355 | 2.55E+02 | 1.99E+02 | 4.97E+00 | 2.54E+02 | [M+H]+ | C10208 | Daidzein | Isoflavone | 4.73E+03 | 7.43E+03 | 1.11E+04 | 1.02E+04 | 1.01E+04 | 8.57E+03 | 1.13E+04 | 6.05E+03 | 8.68E+03 | 6.16E+03 | 4.63E+03 | 5.40E+03 | 1.03E+04 | 7.22E+03 | 8.76E+03 |
| pme0359 | 4.33E+02 | 3.13E+02 | 3.81E+00 | 4.32E+02 | [M+H]+ | - | Apigenin 5-O-glucoside | Flavone | 1.38E+06 | 1.32E+06 | 1.18E+06 | 1.27E+06 | 1.27E+06 | 1.10E+06 | 3.44E+05 | 3.17E+05 | 3.31E+05 | 5.23E+05 | 4.60E+05 | 4.92E+05 | 3.74E+05 | 1.65E+05 | 2.70E+05 |
| pme0363 | 2.99E+02 | 2.84E+02 | 5.76E+00 | 3.00E+02 | [M-H]- | C04293 | Chrysoeriol | Flavone | 2.37E+04 | 2.13E+04 | 2.60E+04 | 2.54E+04 | 2.41E+04 | 2.13E+04 | 1.34E+04 | 1.42E+04 | 1.38E+04 | 3.36E+04 | 3.76E+04 | 3.56E+04 | 1.69E+04 | 2.10E+04 | 1.90E+04 |
| pme0367 | 5.77E+02 | 2.69E+02 | 4.01E+00 | 5.78E+02 | [M-H]- | - | Apigenin 7-rutinoside (Isorhoifolin) | Flavone | 2.82E+04 | 3.18E+04 | 1.89E+04 | 2.58E+04 | 2.41E+04 | 2.29E+04 | 4.19E+04 | 3.89E+04 | 4.04E+04 | 2.67E+04 | 3.58E+04 | 3.13E+04 | 4.22E+04 | 5.77E+04 | 5.00E+04 |
| pme0369 | 5.93E+02 | 2.85E+02 | 3.83E+00 | 5.94E+02 | [M-H]- | - | Kaempferol 3-O-rutinoside (Nicotiflorin) | Flavonol | 2.16E+06 | 2.04E+06 | 2.06E+06 | 2.09E+06 | 2.07E+06 | 1.91E+06 | 1.57E+06 | 1.45E+06 | 1.51E+06 | 1.90E+06 | 1.21E+06 | 1.56E+06 | 1.92E+06 | 1.95E+06 | 1.94E+06 |
| pme0371 | 4.33E+02 | 1.23E+02 | 4.22E+00 | 4.34E+02 | [M-H]- | C09099 | Naringenin 7-O-glucoside (Prunin) | Flavanone | 8.86E+05 | 8.56E+05 | 8.85E+05 | 9.25E+05 | 8.97E+05 | 9.36E+05 | 9.92E+05 | 1.00E+06 | 9.96E+05 | 3.40E+05 | 5.00E+05 | 4.20E+05 | 7.53E+05 | 5.95E+05 | 6.74E+05 |
| pme0374 | 4.31E+02 | 4.31E+02 | 3.79E+00 | 4.32E+02 | [M-H]- | C01714 | Isovitexin | Flavone C-glycosides | 1.50E+04 | 2.05E+04 | 2.53E+04 | 1.81E+04 | 2.10E+04 | 2.47E+04 | 3.92E+04 | 3.10E+04 | 3.51E+04 | 3.91E+04 | 3.41E+04 | 3.66E+04 | 3.39E+04 | 4.32E+04 | 3.86E+04 |
| pme0376 | 2.71E+02 | 1.51E+02 | 5.59E+00 | 2.72E+02 | [M-H]- | C00509 | Naringenin | Flavanone | 4.29E+06 | 4.47E+06 | 4.49E+06 | 4.28E+06 | 4.43E+06 | 4.31E+06 | 6.61E+06 | 6.94E+06 | 6.78E+06 | 2.80E+06 | 3.32E+06 | 3.06E+06 | 2.47E+06 | 2.63E+06 | 2.55E+06 |
| pme0387 | 1.81E+02 | 1.37E+02 | 3.04E+00 | 1.82E+02 | [M-H]- | C05582 | Homovanillic acid | Hydroxycinnamoyl derivatives | 6.86E+03 | 5.44E+03 | 6.28E+03 | 5.94E+03 | 8.57E+03 | 5.93E+03 | 8.79E+03 | 1.02E+04 | 9.50E+03 | 7.46E+03 | 7.08E+03 | 7.27E+03 | 5.82E+03 | 5.95E+03 | 5.89E+03 |
| pme0397 | 1.73E+02 | 9.29E+01 | 8.10E-01 | 1.74E+02 | [M-H]- | C00493 | Shikimic acid | Organic acids | 1.23E+05 | 1.00E+05 | 1.15E+05 | 1.04E+05 | 9.80E+04 | 1.08E+05 | 1.23E+05 | 9.67E+04 | 1.10E+05 | 5.08E+04 | 5.64E+04 | 5.36E+04 | 4.54E+04 | 4.19E+04 | 4.37E+04 |
| pme0399 | 3.55E+02 | 3.55E+02 | 2.72E+00 | 3.54E+02 | [M+H]+ | C00852 | Chlorogenic acid (3-O-Caffeoylquinic acid) | Quinate and its derivatives | 1.21E+06 | 1.18E+06 | 1.13E+06 | 1.11E+06 | 1.14E+06 | 9.89E+05 | 2.01E+06 | 2.04E+06 | 2.03E+06 | 2.51E+06 | 2.05E+06 | 2.28E+06 | 1.93E+06 | 1.46E+06 | 1.70E+06 |
| pme0408 | 1.97E+02 | 1.23E+02 | 3.41E+00 | 1.98E+02 | [M-H]- | C10833 | Syringic acid | Hydroxycinnamoyl derivatives | 9.36E+03 | 1.19E+04 | 8.38E+03 | 7.99E+03 | 1.00E+04 | 8.29E+03 | 9.87E+03 | 7.96E+03 | 8.92E+03 | 9.25E+03 | 7.30E+03 | 8.28E+03 | 7.84E+03 | 8.44E+03 | 8.14E+03 |
| pme0413 | 1.51E+02 | 1.36E+02 | 4.00E+00 | 1.52E+02 | [M-H]- | C00755 | Vanillin | Benzoic acid derivatives | 6.48E+05 | 6.32E+05 | 6.32E+05 | 6.18E+05 | 6.94E+05 | 6.03E+05 | 6.89E+05 | 6.42E+05 | 6.66E+05 | 5.08E+05 | 5.77E+05 | 5.43E+05 | 5.22E+05 | 5.90E+05 | 5.56E+05 |
| pme0418 | 1.65E+02 | 9.29E+01 | 3.71E+00 | 1.66E+02 | [M-H]- | C01744 | 3-(4-Hydroxyphenyl)propionic acid | Hydroxycinnamoyl derivatives | 2.16E+05 | 2.16E+05 | 2.23E+05 | 2.22E+05 | 2.04E+05 | 2.23E+05 | 2.02E+05 | 1.82E+05 | 1.92E+05 | 6.18E+04 | 1.78E+05 | 1.20E+05 | 2.49E+05 | 2.10E+05 | 2.30E+05 |
| pme0422 | 1.93E+02 | 1.34E+02 | 4.08E+00 | 1.94E+02 | [M-H]- | - | 3-Hydroxy-4-methoxycinnamic acid | Hydroxycinnamoyl derivatives | 9.42E+06 | 9.48E+06 | 9.13E+06 | 8.91E+06 | 8.86E+06 | 8.39E+06 | 1.49E+07 | 1.45E+07 | 1.47E+07 | 8.17E+06 | 1.22E+07 | 1.02E+07 | 8.11E+06 | 7.53E+06 | 7.82E+06 |
| pme0424 | 1.33E+02 | 1.15E+02 | 5.97E+00 | 1.32E+02 | [M+H]+ | C00903 | trans-cinnamaldehyde | Hydroxycinnamoyl derivatives | 3.24E+04 | 2.70E+04 | 2.40E+04 | 3.13E+04 | 2.78E+04 | 1.83E+04 | 3.18E+04 | 2.50E+04 | 2.84E+04 | 3.08E+04 | 3.41E+04 | 3.25E+04 | 5.05E+04 | 2.61E+04 | 3.83E+04 |
| pme0433 | 5.77E+02 | 4.26E+02 | 4.06E+00 | 5.76E+02 | [M+H]+ | C10237 | Procyanidin A2 | Proanthocyanidins | 7.31E+04 | 6.78E+04 | 5.97E+04 | 5.14E+04 | 5.04E+04 | 5.18E+04 | 5.30E+04 | 4.82E+04 | 5.06E+04 | 5.13E+04 | 3.80E+04 | 4.47E+04 | 5.31E+04 | 3.27E+04 | 4.29E+04 |
| pme0434 | 5.77E+02 | 4.07E+02 | 3.03E+00 | 5.78E+02 | [M-H]- | - | Procyanidin B2 | Proanthocyanidins | 4.45E+04 | 3.45E+04 | 2.90E+04 | 2.24E+04 | 1.97E+04 | 2.12E+04 | 1.93E+04 | 2.28E+04 | 2.11E+04 | 1.41E+04 | 2.19E+04 | 1.80E+04 | 1.88E+04 | 3.35E+04 | 2.62E+04 |
| pme0442 | 3.03E+02 | 1.49E+02 | 2.98E+00 | 3.03E+02 | Protonated | C05908 | Delphinidin | Anthocyanins | 6.13E+06 | 5.71E+06 | 5.41E+06 | 5.88E+06 | 4.68E+06 | 5.62E+06 | 5.26E+06 | 5.27E+06 | 5.27E+06 | 5.76E+06 | 5.34E+06 | 5.55E+06 | 4.94E+06 | 4.86E+06 | 4.90E+06 |
| pme0450 | 2.89E+02 | 7.88E+01 | 3.32E+00 | 2.90E+02 | [M-H]- | C09727 | L-Epicatechin | Catechin derivatives | 1.32E+05 | 1.45E+05 | 1.46E+05 | 1.25E+05 | 1.32E+05 | 1.33E+05 | 1.58E+05 | 1.28E+05 | 1.43E+05 | 5.10E+04 | 8.24E+04 | 6.67E+04 | 8.00E+04 | 1.37E+05 | 1.09E+05 |
| pme0486 | 1.17E+02 | 1.00E+02 | 1.33E+00 | 1.18E+02 | [M-H]- | C02170 | Methylmalonic acid | Organic acids | 1.84E+07 | 1.78E+07 | 1.69E+07 | 1.54E+07 | 1.60E+07 | 1.56E+07 | 3.21E+07 | 3.24E+07 | 3.23E+07 | 1.80E+07 | 2.31E+07 | 2.06E+07 | 1.24E+07 | 1.81E+07 | 1.53E+07 |
| pme0490 | 1.24E+02 | 7.80E+01 | 1.16E+00 | 1.23E+02 | [M+H]+ | C00253 | Nicotinic acid | Nicotinic acid derivatives | 9.83E+06 | 1.32E+07 | 1.14E+07 | 1.16E+07 | 1.22E+07 | 1.13E+07 | 1.25E+07 | 9.83E+06 | 1.12E+07 | 1.97E+07 | 1.23E+07 | 1.60E+07 | 1.37E+07 | 1.38E+07 | 1.38E+07 |
| pme0491 | 1.38E+02 | 9.40E+01 | 1.47E+00 | 1.39E+02 | [M-H]- | C01020 | 6-hydroxynicotinic acid | Nicotinic acid derivatives | 3.03E+05 | 3.24E+05 | 2.84E+05 | 3.01E+05 | 3.19E+05 | 2.83E+05 | 2.79E+05 | 2.47E+05 | 2.63E+05 | 5.41E+05 | 4.01E+05 | 4.71E+05 | 5.10E+05 | 4.23E+05 | 4.67E+05 |
| pme0500 | 5.03E+02 | 3.23E+02 | 8.10E-01 | 5.04E+02 | [M-H]- | - | D(+)-Melezitose | Carbohydrates | 3.48E+04 | 3.43E+04 | 2.97E+04 | 3.63E+04 | 3.10E+04 | 3.23E+04 | 5.52E+04 | 5.32E+04 | 5.42E+04 | 7.46E+04 | 5.12E+04 | 6.29E+04 | 4.34E+04 | 3.83E+04 | 4.09E+04 |
| pme0516 | 1.79E+02 | 8.70E+01 | 7.40E-01 | 1.80E+02 | [M-H]- | C00137 | Inositol | Others | 3.05E+06 | 2.95E+06 | 2.79E+06 | 3.05E+06 | 2.98E+06 | 2.72E+06 | 2.56E+06 | 2.41E+06 | 2.49E+06 | 2.76E+06 | 2.31E+06 | 2.54E+06 | 2.22E+06 | 2.21E+06 | 2.22E+06 |
| pme0519 | 3.41E+02 | 5.88E+01 | 8.00E-01 | 3.42E+02 | [M-H]- | - | D-(+)-Sucrose | Carbohydrates | 9.95E+06 | 1.04E+07 | 9.87E+06 | 9.97E+06 | 9.88E+06 | 9.23E+06 | 1.15E+07 | 1.26E+07 | 1.21E+07 | 1.11E+07 | 1.06E+07 | 1.09E+07 | 7.97E+06 | 8.65E+06 | 8.31E+06 |
| pme0534 | 1.95E+02 | 7.51E+01 | 6.30E-01 | 1.96E+02 | [M-H]- | C00257 | Gluconic acid | Carbohydrates | 4.59E+06 | 4.52E+06 | 4.39E+06 | 4.71E+06 | 4.52E+06 | 4.32E+06 | 4.34E+06 | 4.05E+06 | 4.20E+06 | 5.68E+06 | 4.49E+06 | 5.09E+06 | 5.34E+06 | 5.23E+06 | 5.29E+06 |
| pme0543 | 1.60E+02 | 1.16E+02 | 4.37E+00 | 1.61E+02 | [M-H]- | - | Indole-5-carboxylic acid | Indole derivatives | 4.26E+04 | 4.43E+04 | 4.98E+04 | 4.38E+04 | 4.03E+04 | 4.43E+04 | 5.35E+04 | 5.09E+04 | 5.22E+04 | 3.34E+04 | 4.98E+04 | 4.16E+04 | 9.00E+00 | 2.11E+04 | 1.06E+04 |
| pme1002 | 1.38E+02 | 1.03E+02 | 1.26E+00 | 1.37E+02 | [M+H]+ | C00483 | L-Tyramine | Amino acids | 1.03E+07 | 1.11E+07 | 1.18E+07 | 1.25E+07 | 1.14E+07 | 1.16E+07 | 8.23E+06 | 7.71E+06 | 7.97E+06 | 7.74E+06 | 9.63E+06 | 8.69E+06 | 2.47E+07 | 2.27E+07 | 2.37E+07 |
| pme1014 | 4.45E+02 | 2.81E+02 | 1.08E+01 | 4.44E+02 | [M+H]+ | C00828 | Menaquinone (K2) | Vitamins | 2.38E+06 | 2.18E+06 | 2.69E+06 | 2.80E+06 | 2.89E+06 | 3.70E+06 | 6.04E+05 | 4.25E+05 | 5.15E+05 | 6.36E+05 | 5.78E+05 | 6.07E+05 | 4.25E+05 | 4.58E+05 | 4.42E+05 |
| pme1018 | 6.13E+02 | 3.56E+02 | 1.16E+00 | 6.12E+02 | [M+H]+ | C00127 | Glutathione oxidized | Amino acid derivatives | 1.86E+06 | 2.19E+06 | 2.10E+06 | 1.83E+06 | 2.16E+06 | 1.94E+06 | 2.57E+06 | 2.41E+06 | 2.49E+06 | 1.37E+06 | 1.76E+06 | 1.57E+06 | 2.05E+06 | 1.57E+06 | 1.81E+06 |
| pme1021 | 1.77E+02 | 7.08E+01 | 8.60E-01 | 1.78E+02 | [M-H]- | C00198 | "D-(+)-Glucono-1,5-lactone" | Carbohydrates | 2.58E+06 | 2.56E+06 | 2.61E+06 | 2.62E+06 | 2.60E+06 | 2.53E+06 | 2.24E+06 | 2.78E+06 | 2.51E+06 | 2.14E+06 | 1.95E+06 | 2.05E+06 | 2.46E+06 | 2.42E+06 | 2.44E+06 |
| pme1055 | 2.07E+02 | 1.44E+02 | 1.92E+00 | 2.08E+02 | [M-H]- | C00328 | L-Kynurenine | Amino acid derivatives | 9.50E+04 | 9.47E+04 | 8.69E+04 | 9.68E+04 | 9.61E+04 | 8.76E+04 | 7.76E+04 | 7.73E+04 | 7.75E+04 | 6.82E+04 | 1.07E+05 | 8.76E+04 | 9.62E+04 | 1.23E+05 | 1.10E+05 |
| pme1090 | 3.08E+02 | 1.62E+02 | 1.16E+00 | 3.07E+02 | [M+H]+ | C00051 | Glutathione reduced form | Amino acid derivatives | 9.47E+05 | 1.03E+06 | 8.85E+05 | 8.63E+05 | 9.42E+05 | 7.58E+05 | 1.10E+05 | 1.21E+05 | 1.16E+05 | 1.34E+05 | 1.12E+05 | 1.23E+05 | 2.25E+05 | 1.74E+05 | 2.00E+05 |
| pme1097 | 1.34E+02 | 1.34E+02 | 1.17E+00 | 1.35E+02 | [M-H]- | C00147 | Adenine | Nucleotide and its derivates | 1.69E+06 | 1.75E+06 | 1.50E+06 | 1.39E+06 | 1.44E+06 | 1.35E+06 | 2.79E+06 | 2.35E+06 | 2.57E+06 | 1.30E+06 | 1.77E+06 | 1.54E+06 | 1.84E+06 | 1.46E+06 | 1.65E+06 |
| pme1104 | 1.30E+02 | 1.30E+02 | 1.26E+00 | 1.31E+02 | [M-H]- | C00407 | L-Isoleucine | Amino acids | 1.30E+06 | 1.27E+06 | 1.30E+06 | 1.22E+06 | 1.23E+06 | 1.22E+06 | 1.43E+06 | 1.34E+06 | 1.39E+06 | 8.21E+05 | 1.32E+06 | 1.07E+06 | 1.58E+06 | 1.52E+06 | 1.55E+06 |
| pme1109 | 1.52E+02 | 1.35E+02 | 1.32E+00 | 1.51E+02 | [M+H]+ | C00242 | Guanine | Nucleotide and its derivates | 9.90E+05 | 9.88E+05 | 8.66E+05 | 8.39E+05 | 8.47E+05 | 8.27E+05 | 1.26E+06 | 9.80E+05 | 1.12E+06 | 4.56E+05 | 6.96E+05 | 5.76E+05 | 4.65E+05 | 1.30E+06 | 8.83E+05 |
| pme1119 | 2.69E+02 | 1.37E+02 | 8.30E-01 | 2.68E+02 | [M+H]+ | C00294 | Inosine | Nucleotide and its derivates | 6.12E+04 | 4.68E+04 | 4.65E+04 | 6.12E+04 | 5.66E+04 | 5.20E+04 | 5.37E+04 | 5.56E+04 | 5.47E+04 | 4.74E+04 | 3.65E+04 | 4.20E+04 | 4.35E+04 | 3.68E+04 | 4.02E+04 |
| pme1178 | 2.84E+02 | 1.35E+02 | 1.52E+00 | 2.83E+02 | [M+H]+ | C00387 | Guanosine | Nucleotide and its derivates | 1.03E+07 | 1.19E+07 | 1.19E+07 | 1.15E+07 | 1.13E+07 | 1.23E+07 | 1.71E+07 | 1.73E+07 | 1.72E+07 | 7.94E+06 | 1.30E+07 | 1.05E+07 | 1.03E+07 | 9.47E+06 | 9.89E+06 |
| pme1184 | 2.68E+02 | 1.52E+02 | 1.67E+00 | 2.67E+02 | [M+H]+ | C00330 | Deoxyguanosine | Nucleotide and its derivates | 9.14E+05 | 9.02E+05 | 8.99E+05 | 8.95E+05 | 9.01E+05 | 7.75E+05 | 1.08E+06 | 9.50E+05 | 1.02E+06 | 2.87E+05 | 4.82E+05 | 3.85E+05 | 1.67E+05 | 3.14E+05 | 2.41E+05 |
| pme1187 | 2.57E+02 | 1.25E+02 | 1.76E+00 | 2.58E+02 | [M-H]- | - | 5-Methyluridine | Nucleotide and its derivates | 1.16E+04 | 1.04E+04 | 1.07E+04 | 1.08E+04 | 1.10E+04 | 1.12E+04 | 2.45E+04 | 1.71E+04 | 2.08E+04 | 1.73E+04 | 1.69E+04 | 1.71E+04 | 1.53E+04 | 1.54E+04 | 1.54E+04 |
| pme1194 | 2.28E+02 | 1.12E+02 | 1.16E+00 | 2.27E+02 | [M+H]+ | C00881 | Deoxycytidine | Nucleotide and its derivates | 2.41E+05 | 2.51E+05 | 2.36E+05 | 2.58E+05 | 2.54E+05 | 2.35E+05 | 4.12E+05 | 3.50E+05 | 3.81E+05 | 1.96E+05 | 2.58E+05 | 2.27E+05 | 1.00E+05 | 1.29E+05 | 1.15E+05 |
| pme1210 | 1.50E+02 | 7.28E+01 | 1.15E+00 | 1.49E+02 | [M+H]+ | C00073 | L-Methionine | Amino acids | 5.35E+06 | 6.29E+06 | 5.91E+06 | 5.95E+06 | 6.34E+06 | 5.94E+06 | 4.09E+06 | 3.49E+06 | 3.79E+06 | 5.76E+06 | 5.54E+06 | 5.65E+06 | 8.22E+06 | 7.04E+06 | 7.63E+06 |
| pme1218 | 1.24E+02 | 7.78E+01 | 1.15E+00 | 1.23E+02 | [M+H]+ | C10164 | 2-Picolinic acid | Organic acids | 1.74E+06 | 2.08E+06 | 2.06E+06 | 1.88E+06 | 2.10E+06 | 1.81E+06 | 1.74E+06 | 1.63E+06 | 1.69E+06 | 3.52E+06 | 2.09E+06 | 2.81E+06 | 2.45E+06 | 2.35E+06 | 2.40E+06 |
| pme1228 | 2.21E+02 | 1.15E+02 | 1.66E+00 | 2.20E+02 | [M+H]+ | C00643 | 5-Hydroxy-L-tryptophan | Amino acid derivatives | 2.28E+05 | 2.63E+05 | 2.07E+05 | 3.12E+05 | 2.15E+05 | 2.11E+05 | 1.78E+05 | 1.52E+05 | 1.65E+05 | 2.47E+05 | 2.75E+05 | 2.61E+05 | 4.88E+05 | 5.37E+05 | 5.13E+05 |
| pme1239 | 3.20E+02 | 1.28E+02 | 1.27E+00 | 3.21E+02 | [M-H]- | C11347 | S-(methyl)glutathione | Amino acid derivatives | 9.99E+04 | 1.14E+05 | 8.76E+04 | 1.15E+05 | 1.02E+05 | 9.25E+04 | 5.11E+04 | 5.67E+04 | 5.39E+04 | 9.38E+04 | 9.42E+04 | 9.40E+04 | 9.59E+04 | 9.54E+04 | 9.57E+04 |
| pme1261 | 2.06E+02 | 1.88E+02 | 2.18E+00 | 2.05E+02 | [M+H]+ | C05944 | Pantothenol | Alcohols and polyols | 1.18E+04 | 1.23E+04 | 1.60E+04 | 1.50E+04 | 1.29E+04 | 1.50E+04 | 1.54E+04 | 1.50E+04 | 1.52E+04 | 1.39E+04 | 8.74E+03 | 1.13E+04 | 1.85E+04 | 3.63E+04 | 2.74E+04 |
| pme1286 | 3.85E+02 | 1.36E+02 | 1.38E+00 | 3.84E+02 | [M+H]+ | C00021 | S-(5'-Adenosy)-L-homocysteine | Amino acid derivatives | 3.63E+05 | 3.96E+05 | 3.57E+05 | 3.82E+05 | 3.93E+05 | 4.24E+05 | 3.22E+05 | 2.82E+05 | 3.02E+05 | 3.49E+05 | 3.04E+05 | 3.27E+05 | 3.04E+05 | 3.01E+05 | 3.03E+05 |
| pme1294 | 2.83E+02 | 1.51E+02 | 1.75E+00 | 2.84E+02 | [M-H]- | C01762 | Xanthosine | Nucleotide and its derivates | 5.36E+06 | 5.20E+06 | 5.06E+06 | 4.83E+06 | 5.05E+06 | 5.27E+06 | 5.43E+06 | 5.57E+06 | 5.50E+06 | 1.72E+06 | 2.79E+06 | 2.26E+06 | 1.70E+06 | 2.88E+06 | 2.29E+06 |
| pme1306 | 2.50E+02 | 1.34E+02 | 1.23E+00 | 2.49E+02 | [M+H]+ | C00627 | Pyridoxine 5'-phosphate | Vitamins | 1.07E+05 | 1.24E+05 | 1.06E+05 | 8.62E+04 | 1.15E+05 | 1.10E+05 | 9.49E+04 | 8.79E+04 | 9.14E+04 | 8.08E+04 | 7.63E+04 | 7.86E+04 | 8.09E+04 | 1.11E+05 | 9.60E+04 |
| pme1313 | 2.37E+02 | 1.46E+02 | 1.98E+00 | 2.36E+02 | [M+H]+ | C02700 | N'-Formylkynurenine | Amino acid derivatives | 7.91E+04 | 1.03E+05 | 8.41E+04 | 1.06E+05 | 1.09E+05 | 1.09E+05 | 9.47E+04 | 1.22E+05 | 1.08E+05 | 8.52E+04 | 1.57E+05 | 1.21E+05 | 9.63E+04 | 1.54E+05 | 1.25E+05 |
| pme1346 | 3.00E+02 | 1.68E+02 | 1.59E+00 | 2.99E+02 | [M+H]+ | - | 8-Hydroxyguanosine | Nucleotide and its derivates | 3.41E+05 | 3.48E+05 | 2.71E+05 | 3.24E+05 | 3.46E+05 | 3.09E+05 | 3.27E+05 | 3.52E+05 | 3.40E+05 | 1.99E+05 | 5.08E+05 | 3.54E+05 | 3.19E+05 | 3.36E+05 | 3.28E+05 |
| pme1368 | 1.28E+02 | 1.26E+02 | 1.16E+00 | 1.29E+02 | [M-H]- | C00408 | L-Pipecolic acid | Amino acid derivatives | 3.33E+05 | 3.51E+05 | 3.16E+05 | 3.69E+05 | 4.18E+05 | 3.43E+05 | 1.75E+05 | 1.62E+05 | 1.69E+05 | 8.58E+04 | 2.17E+05 | 1.51E+05 | 1.21E+05 | 7.03E+04 | 9.57E+04 |
| pme1373 | 3.06E+02 | 7.88E+01 | 1.17E+00 | 3.07E+02 | [M-H]- | C00239 | 2'-Deoxycytidine-5'-monophosphate | Nucleotide and its derivates | 6.69E+04 | 6.85E+04 | 7.64E+04 | 7.28E+04 | 8.82E+04 | 8.61E+04 | 4.16E+04 | 3.97E+04 | 4.07E+04 | 6.33E+04 | 7.56E+04 | 6.95E+04 | 9.57E+04 | 7.33E+04 | 8.45E+04 |
| pme1378 | 2.82E+02 | 1.50E+02 | 2.27E+00 | 2.81E+02 | [M+H]+ | C02494 | 1-Methyladenosine | Nucleotide and its derivates | 5.10E+03 | 5.42E+03 | 4.79E+03 | 2.78E+03 | 4.04E+03 | 5.23E+03 | 6.99E+03 | 3.89E+03 | 5.44E+03 | 1.49E+04 | 8.19E+03 | 1.15E+04 | 1.47E+04 | 6.15E+03 | 1.04E+04 |
| pme1383 | 1.70E+02 | 1.34E+02 | 1.16E+00 | 1.69E+02 | [M+H]+ | C00314 | Pyridoxine | Vitamins | 4.10E+06 | 4.72E+06 | 4.54E+06 | 4.64E+06 | 4.47E+06 | 4.31E+06 | 6.47E+06 | 6.13E+06 | 6.30E+06 | 1.38E+06 | 3.26E+06 | 2.32E+06 | 8.08E+05 | 1.22E+06 | 1.01E+06 |
| pme1397 | 2.71E+02 | 2.15E+02 | 3.85E+00 | 2.71E+02 | Protonated | C05904 | Pelargonidin | Anthocyanins | 3.88E+04 | 3.26E+04 | 3.00E+04 | 4.86E+04 | 4.34E+04 | 4.69E+04 | 2.04E+04 | 1.22E+04 | 1.63E+04 | 1.56E+04 | 3.27E+04 | 2.42E+04 | 2.06E+04 | 1.35E+04 | 1.71E+04 |
| pme1398 | 4.65E+02 | 3.03E+02 | 2.26E+00 | 4.65E+02 | Protonated | C12138 | Delphinidin 3-O-glucoside (Mirtillin) | Anthocyanins | 2.11E+05 | 2.26E+05 | 2.34E+05 | 2.61E+05 | 2.48E+05 | 2.14E+05 | 2.81E+05 | 3.35E+05 | 3.08E+05 | 3.84E+05 | 2.89E+05 | 3.37E+05 | 1.87E+05 | 3.01E+05 | 2.44E+05 |
| pme1399 | 3.55E+02 | 1.79E+02 | 8.40E+00 | 3.54E+02 | [M+H]+ | C16417 | Xanthohumol | Flavanone | 3.79E+04 | 3.85E+04 | 4.01E+04 | 3.69E+04 | 3.58E+04 | 3.44E+04 | 6.16E+04 | 6.49E+04 | 6.33E+04 | 6.78E+04 | 4.97E+04 | 5.88E+04 | 5.38E+04 | 9.47E+04 | 7.43E+04 |
| pme1408 | 1.45E+02 | 1.27E+02 | 7.60E-01 | 1.46E+02 | [M-H]- | C00064 | L-Glutamine | Amino acids | 1.44E+07 | 1.43E+07 | 1.32E+07 | 1.41E+07 | 1.44E+07 | 1.24E+07 | 1.34E+07 | 1.25E+07 | 1.30E+07 | 4.10E+06 | 1.27E+07 | 8.40E+06 | 3.92E+06 | 3.84E+06 | 3.88E+06 |
| pme1417 | 1.61E+02 | 1.44E+02 | 2.74E+00 | 1.60E+02 | [M+H]+ | C00398 | L-Tryptamine | Tryptamine derivatives | 5.00E+06 | 5.87E+06 | 5.63E+06 | 5.98E+06 | 6.70E+06 | 5.67E+06 | 2.23E+06 | 2.37E+06 | 2.30E+06 | 4.76E+06 | 2.71E+06 | 3.74E+06 | 1.17E+06 | 3.65E+06 | 2.41E+06 |
| pme1419 | 1.64E+02 | 6.10E+01 | 1.77E+00 | 1.63E+02 | [M+H]+ | - | L-Methionine methyl ester | Amino acid derivatives | 3.33E+05 | 3.31E+05 | 4.22E+05 | 2.59E+05 | 3.02E+05 | 4.64E+05 | 2.03E+05 | 1.51E+05 | 1.77E+05 | 2.95E+05 | 1.69E+05 | 2.32E+05 | 4.89E+05 | 3.33E+05 | 4.11E+05 |
| pme1424 | 1.77E+02 | 1.77E+02 | 4.64E+00 | 1.78E+02 | [M-H]- | C02666 | Coniferylaldehyde | Hydroxycinnamoyl derivatives | 1.47E+06 | 1.54E+06 | 1.53E+06 | 1.46E+06 | 1.46E+06 | 1.42E+06 | 1.63E+06 | 1.62E+06 | 1.63E+06 | 1.41E+06 | 1.30E+06 | 1.36E+06 | 9.47E+05 | 1.23E+06 | 1.09E+06 |
| pme1434 | 3.71E+02 | 2.09E+02 | 2.58E+00 | 3.72E+02 | [M-H]- | C01533 | Syringin | Hydroxycinnamoyl derivatives | 1.11E+05 | 1.19E+05 | 1.25E+05 | 1.21E+05 | 1.09E+05 | 1.16E+05 | 9.65E+04 | 8.70E+04 | 9.18E+04 | 6.98E+04 | 7.38E+04 | 7.18E+04 | 5.49E+04 | 8.23E+04 | 6.86E+04 |
| pme1436 | 1.63E+02 | 1.19E+02 | 3.86E+00 | 1.64E+02 | [M-H]- | C00811 | p-Coumaric acid | Hydroxycinnamoyl derivatives | 8.98E+05 | 8.65E+05 | 8.80E+05 | 8.76E+05 | 8.71E+05 | 8.53E+05 | 8.02E+05 | 8.28E+05 | 8.15E+05 | 2.16E+05 | 6.79E+05 | 4.48E+05 | 8.99E+05 | 8.29E+05 | 8.64E+05 |
| pme1473 | 2.96E+02 | 1.34E+02 | 2.90E+00 | 2.97E+02 | [M-H]- | C00170 | 5'-Deoxy-5'-(methylthio)adenosine | Nucleotide and its derivates | 2.02E+05 | 2.17E+05 | 2.18E+05 | 2.12E+05 | 2.09E+05 | 2.20E+05 | 2.48E+05 | 2.38E+05 | 2.43E+05 | 1.81E+05 | 1.91E+05 | 1.86E+05 | 1.31E+05 | 1.18E+05 | 1.25E+05 |
| pme1478 | 3.17E+02 | 1.79E+02 | 4.70E+00 | 3.18E+02 | [M-H]- | C10107 | Myricetin | Flavonol | 4.98E+04 | 5.02E+04 | 5.34E+04 | 4.95E+04 | 4.85E+04 | 4.44E+04 | 8.86E+04 | 7.86E+04 | 8.36E+04 | 5.64E+04 | 5.21E+04 | 5.43E+04 | 2.26E+04 | 3.42E+04 | 2.84E+04 |
| pme1486 | 4.57E+02 | 1.69E+02 | 3.33E+00 | 4.58E+02 | [M-H]- | C09731 | Epigallate catechin gallate (EGCG) | Catechin derivatives | 1.29E+05 | 8.38E+04 | 7.54E+04 | 7.71E+04 | 6.93E+04 | 6.58E+04 | 7.67E+03 | 5.85E+03 | 6.76E+03 | 1.24E+04 | 1.61E+04 | 1.43E+04 | 1.68E+04 | 2.48E+05 | 1.32E+05 |
| pme1496 | 2.67E+02 | 2.52E+02 | 6.33E+00 | 2.68E+02 | [M-H]- | C00858 | Formononetin (4'-O-methyldaidzein) | Isoflavone | 5.32E+03 | 7.29E+03 | 6.65E+03 | 6.35E+03 | 5.09E+03 | 5.78E+03 | 4.87E+03 | 4.25E+03 | 4.56E+03 | 5.07E+03 | 4.72E+03 | 4.90E+03 | 4.85E+03 | 7.05E+03 | 5.95E+03 |
| pme1502 | 3.15E+02 | 3.00E+02 | 7.23E+00 | 3.14E+02 | [M+H]+ | - | Kumatakenin | Flavonol | 1.17E+04 | 1.19E+04 | 1.31E+04 | 1.26E+04 | 1.14E+04 | 1.15E+04 | 2.79E+04 | 1.76E+04 | 2.28E+04 | 9.00E+00 | 4.51E+04 | 2.26E+04 | 4.66E+03 | 1.65E+04 | 1.06E+04 |
| pme1514 | 3.05E+02 | 1.25E+02 | 2.76E+00 | 3.06E+02 | [M-H]- | C12136 | Epigallocatechin (EGC) | Catechin derivatives | 5.96E+04 | 6.89E+04 | 5.93E+04 | 6.84E+04 | 6.37E+04 | 6.85E+04 | 4.91E+04 | 6.84E+04 | 5.88E+04 | 4.91E+04 | 8.30E+04 | 6.61E+04 | 8.95E+04 | 5.70E+04 | 7.33E+04 |
| pme1518 | 4.03E+02 | 3.73E+02 | 7.06E+00 | 4.02E+02 | [M+H]+ | C10112 | Nobiletin | Flavone | 7.40E+04 | 7.57E+04 | 7.12E+04 | 7.27E+04 | 7.33E+04 | 7.51E+04 | 5.46E+04 | 5.11E+04 | 5.29E+04 | 4.87E+04 | 5.61E+04 | 5.24E+04 | 6.24E+04 | 4.85E+04 | 5.55E+04 |
| pme1521 | 3.03E+02 | 1.25E+02 | 4.15E+00 | 3.04E+02 | [M-H]- | C01617 | Dihydroquercetin (Taxifolin) | Flavonol | 7.58E+04 | 8.14E+04 | 7.11E+04 | 8.31E+04 | 7.38E+04 | 6.79E+04 | 8.92E+04 | 1.08E+05 | 9.86E+04 | 4.81E+04 | 4.07E+04 | 4.44E+04 | 3.31E+04 | 5.56E+04 | 4.44E+04 |
| pme1537 | 3.07E+02 | 2.48E+02 | 2.27E+00 | 3.06E+02 | [M+H]+ | C12127 | (+)-Gallocatechin (GC) | Catechin derivatives | 1.96E+04 | 3.09E+04 | 2.63E+04 | 2.53E+04 | 2.12E+04 | 2.71E+04 | 6.82E+03 | 4.09E+03 | 5.46E+03 | 9.00E+00 | 9.00E+00 | 9.00E+00 | 7.50E+03 | 1.86E+04 | 1.31E+04 |
| pme1540 | 6.25E+02 | 3.17E+02 | 3.66E+00 | 6.24E+02 | [M+H]+ | - | Isorhamnetin 3-O-neohesperidoside | Flavonol | 4.72E+05 | 4.15E+05 | 4.13E+05 | 4.41E+05 | 3.54E+05 | 3.61E+05 | 1.92E+05 | 1.72E+05 | 1.82E+05 | 5.80E+05 | 2.66E+05 | 4.23E+05 | 3.18E+05 | 2.10E+05 | 2.64E+05 |
| pme1541 | 2.83E+02 | 2.68E+02 | 7.06E+00 | 2.84E+02 | [M-H]- | C01470 | Acacetin | Flavone | 2.50E+03 | 2.98E+03 | 2.03E+03 | 3.00E+03 | 2.66E+03 | 2.66E+03 | 9.00E+00 | 3.26E+03 | 1.63E+03 | 2.32E+03 | 8.14E+03 | 5.23E+03 | 3.36E+03 | 4.34E+03 | 3.85E+03 |
| pme1550 | 3.73E+02 | 3.73E+02 | 7.54E+00 | 3.72E+02 | [M+H]+ | C10190 | Tangeretin | Flavone | 5.92E+04 | 5.15E+04 | 5.91E+04 | 6.18E+04 | 6.02E+04 | 6.32E+04 | 5.20E+04 | 4.65E+04 | 4.93E+04 | 4.83E+04 | 4.45E+04 | 4.64E+04 | 5.81E+04 | 4.87E+04 | 5.34E+04 |
| pme1562 | 4.41E+02 | 2.89E+02 | 3.89E+00 | 4.42E+02 | [M-H]- | - | Epicatechin gallate (ECG) | Catechin derivatives | 2.77E+04 | 1.91E+04 | 2.15E+04 | 2.09E+04 | 1.80E+04 | 2.46E+04 | 9.00E+00 | 9.00E+00 | 9.00E+00 | 1.47E+04 | 9.00E+00 | 7.35E+03 | 9.00E+00 | 6.78E+04 | 3.39E+04 |
| pme1583 | 2.89E+02 | 1.53E+02 | 5.05E+00 | 2.88E+02 | [M+H]+ | C05631 | Eriodictyol | Flavanone | 3.48E+04 | 2.13E+04 | 2.25E+04 | 1.84E+04 | 1.73E+04 | 1.27E+04 | 2.02E+04 | 1.53E+04 | 1.78E+04 | 9.00E+00 | 9.00E+00 | 9.00E+00 | 9.00E+00 | 9.00E+00 | 9.00E+00 |
| pme1588 | 3.15E+02 | 3.00E+02 | 5.85E+00 | 3.16E+02 | [M-H]- | C10084 | Isorhamnetin | Flavonol | 4.06E+03 | 4.92E+03 | 4.30E+03 | 4.59E+03 | 5.33E+03 | 5.73E+03 | 4.81E+03 | 5.74E+03 | 5.28E+03 | 2.81E+03 | 5.62E+03 | 4.22E+03 | 5.55E+03 | 6.44E+03 | 6.00E+03 |
| pme1598 | 4.63E+02 | 3.01E+02 | 3.85E+00 | 4.64E+02 | [M-H]- | - | Hesperetin 5-O-glucoside | Flavanone | 1.73E+06 | 1.76E+06 | 1.63E+06 | 1.65E+06 | 1.64E+06 | 1.53E+06 | 1.24E+06 | 1.17E+06 | 1.21E+06 | 7.75E+05 | 8.60E+05 | 8.18E+05 | 1.55E+06 | 1.90E+06 | 1.73E+06 |
| pme1601 | 4.17E+02 | 2.71E+02 | 4.90E+00 | 4.16E+02 | [M+H]+ | - | Apigenin 4-O-rhamnoside | Flavone | 4.99E+04 | 4.54E+04 | 5.27E+04 | 4.08E+04 | 4.09E+04 | 3.18E+04 | 8.79E+04 | 4.57E+04 | 6.68E+04 | 1.17E+05 | 7.22E+04 | 9.46E+04 | 7.45E+04 | 9.16E+04 | 8.31E+04 |
| pme1605 | 5.93E+02 | 2.85E+02 | 3.76E+00 | 5.94E+02 | [M-H]- | - | Kaempferol 3-O-robinobioside (Biorobin) | Flavonol | 2.16E+06 | 2.05E+06 | 2.14E+06 | 2.26E+06 | 2.11E+06 | 1.94E+06 | 1.50E+06 | 1.56E+06 | 1.53E+06 | 1.84E+06 | 1.25E+06 | 1.55E+06 | 1.97E+06 | 1.90E+06 | 1.94E+06 |
| pme1611 | 4.33E+02 | 4.33E+02 | 3.75E+00 | 4.34E+02 | [M-H]- | - | Isohemiphloin | Alkaloids | 6.06E+04 | 6.08E+04 | 5.64E+04 | 6.44E+04 | 4.83E+04 | 4.61E+04 | 7.88E+04 | 6.06E+04 | 6.97E+04 | 3.29E+04 | 4.10E+04 | 3.70E+04 | 8.26E+04 | 8.85E+04 | 8.56E+04 |
| pme1622 | 4.47E+02 | 2.87E+02 | 3.93E+00 | 4.48E+02 | [M-H]- | C12249 | Kaempferol 3-O-glucoside (Astragalin) | Flavonol | 7.17E+05 | 6.22E+05 | 6.99E+05 | 6.73E+05 | 6.51E+05 | 5.88E+05 | 4.72E+05 | 4.82E+05 | 4.77E+05 | 2.43E+05 | 2.20E+05 | 2.32E+05 | 2.36E+05 | 2.48E+05 | 2.42E+05 |
| pme1635 | 5.33E+02 | 3.71E+02 | 4.85E+00 | 5.34E+02 | [M-H]- | C16915 | Arctiin | Others | 8.14E+03 | 6.50E+03 | 1.15E+04 | 1.28E+04 | 8.55E+03 | 8.72E+03 | 3.81E+03 | 6.90E+03 | 5.36E+03 | 3.64E+03 | 3.87E+03 | 3.76E+03 | 4.35E+03 | 4.65E+03 | 4.50E+03 |
| pme1640 | 3.71E+02 | 2.67E+02 | 1.19E+01 | 3.70E+02 | [M+H]+ | - | sesamolin | Others | 2.37E+06 | 2.22E+06 | 2.91E+06 | 3.27E+06 | 3.08E+06 | 3.67E+06 | 4.86E+05 | 2.98E+05 | 3.92E+05 | 4.76E+05 | 5.16E+05 | 4.96E+05 | 2.70E+05 | 2.02E+05 | 2.36E+05 |
| pme1646 | 3.57E+02 | 1.36E+02 | 5.41E+00 | 3.58E+02 | [M-H]- | - | Pinoresinol | Hydroxycinnamoyl derivatives | 2.76E+05 | 2.82E+05 | 2.85E+05 | 2.77E+05 | 2.78E+05 | 2.65E+05 | 1.05E+06 | 1.13E+06 | 1.09E+06 | 3.17E+05 | 4.47E+05 | 3.82E+05 | 2.31E+05 | 6.05E+05 | 4.18E+05 |
| pme1662 | 2.87E+02 | 2.87E+02 | 6.96E+00 | 2.86E+02 | [M+H]+ | C09833 | sakuranetin | Flavone | 3.24E+04 | 4.20E+04 | 3.17E+04 | 2.98E+04 | 3.50E+04 | 2.91E+04 | 1.05E+05 | 7.75E+04 | 9.13E+04 | 9.00E+00 | 1.35E+05 | 6.75E+04 | 2.37E+04 | 3.86E+04 | 3.12E+04 |
| pme1667 | 5.95E+02 | 3.67E+02 | 3.36E+00 | 5.94E+02 | [M+H]+ | C08064 | Isovitexin 7-O-glucoside (Saponarin) | Others | 5.33E+04 | 1.03E+05 | 5.88E+04 | 6.70E+04 | 5.46E+04 | 4.51E+04 | 1.04E+05 | 1.03E+05 | 1.04E+05 | 6.70E+04 | 4.48E+04 | 5.59E+04 | 4.37E+05 | 2.05E+05 | 3.21E+05 |
| pme1683 | 4.77E+02 | 2.58E+02 | 2.15E+00 | 4.76E+02 | [M+H]+ | C12276 | D-Pantothenic dcid | Organic acids | 4.29E+04 | 6.85E+04 | 8.81E+04 | 9.30E+04 | 6.65E+04 | 7.03E+04 | 1.09E+05 | 9.30E+04 | 1.01E+05 | 9.85E+04 | 8.16E+04 | 9.01E+04 | 5.66E+04 | 7.66E+04 | 6.66E+04 |
| pme1691 | 1.47E+02 | 8.80E+01 | 8.50E-01 | 1.46E+02 | [M+H]+ | C08201 | Acetylcholine | Cholines | 2.79E+06 | 3.96E+06 | 3.33E+06 | 3.29E+06 | 3.75E+06 | 3.44E+06 | 3.01E+06 | 2.84E+06 | 2.93E+06 | 4.08E+06 | 3.34E+06 | 3.71E+06 | 3.19E+06 | 2.87E+06 | 3.03E+06 |
| pme1695 | 2.23E+02 | 1.49E+02 | 4.06E+00 | 2.24E+02 | [M-H]- | C00482 | Sinapic acid | Hydroxycinnamoyl derivatives | 1.63E+05 | 1.51E+05 | 1.33E+05 | 1.35E+05 | 1.38E+05 | 1.12E+05 | 9.09E+04 | 7.23E+04 | 8.16E+04 | 1.02E+05 | 1.34E+05 | 1.18E+05 | 3.58E+05 | 2.45E+05 | 3.02E+05 |
| pme1709 | 1.38E+02 | 7.80E+01 | 3.73E+00 | 1.37E+02 | [M+H]+ | - | Nicotinic Acid Methyl Ester (Methyl Nicotinate) | Vitamins | 4.18E+06 | 4.64E+06 | 4.97E+06 | 5.25E+06 | 5.12E+06 | 4.96E+06 | 2.13E+06 | 2.07E+06 | 2.10E+06 | 1.46E+06 | 1.88E+06 | 1.67E+06 | 1.38E+06 | 1.69E+06 | 1.54E+06 |
| pme1711 | 1.17E+02 | 9.99E+01 | 2.03E+00 | 1.18E+02 | [M-H]- | - | 3-Hydroxy-3-methyl butyric acid | Organic acids | 1.07E+05 | 1.10E+05 | 1.28E+05 | 8.83E+04 | 9.57E+04 | 1.28E+05 | 1.18E+05 | 1.17E+05 | 1.18E+05 | 1.57E+05 | 1.18E+05 | 1.38E+05 | 1.79E+05 | 1.38E+05 | 1.59E+05 |
| pme1712 | 2.75E+02 | 2.57E+02 | 7.90E-01 | 2.76E+02 | [M-H]- | C00449 | L-Saccharopine | Amino acid derivatives | 9.79E+04 | 1.04E+05 | 1.24E+05 | 1.03E+05 | 1.17E+05 | 1.15E+05 | 1.22E+05 | 1.07E+05 | 1.15E+05 | 1.11E+05 | 1.07E+05 | 1.09E+05 | 1.40E+05 | 1.80E+05 | 1.60E+05 |
| pme1730 | 1.17E+02 | 9.98E+01 | 7.40E-01 | 1.18E+02 | [M-H]- | - | D-Erythronolactone | Organic acids | 7.61E+05 | 7.50E+05 | 6.50E+05 | 6.22E+05 | 7.27E+05 | 7.93E+05 | 7.08E+05 | 6.38E+05 | 6.73E+05 | 6.25E+05 | 5.46E+05 | 5.86E+05 | 6.54E+05 | 6.08E+05 | 6.31E+05 |
| pme1738 | 1.38E+02 | 9.50E+01 | 8.00E-01 | 1.37E+02 | [M+H]+ | C02918 | 3-Carbamyl-1-methylpyridinium (1-Methylnicotinamide) | Pyridine derivatives | 2.43E+04 | 2.77E+04 | 1.58E+04 | 2.28E+04 | 4.75E+04 | 3.92E+04 | 4.79E+04 | 2.51E+04 | 3.65E+04 | 3.83E+04 | 2.82E+04 | 3.33E+04 | 4.26E+04 | 3.24E+04 | 3.75E+04 |
| pme1773 | 5.95E+02 | 2.88E+02 | 2.70E+00 | 5.95E+02 | Protonated | C08620 | Cyanidin 3-O-rutinoside (Keracyanin) | Anthocyanins | 3.40E+04 | 2.86E+04 | 3.85E+04 | 4.79E+04 | 4.42E+04 | 2.59E+04 | 3.96E+04 | 3.40E+04 | 3.68E+04 | 4.85E+04 | 4.30E+04 | 4.58E+04 | 4.55E+04 | 2.72E+04 | 3.64E+04 |
| pme1786 | 6.55E+02 | 3.31E+02 | 2.61E+00 | 6.55E+02 | Protonated | C08718 | "Malvidin 3,5-diglucoside (Malvin)" | Anthocyanins | 4.09E+04 | 4.19E+04 | 3.13E+04 | 5.17E+04 | 4.97E+04 | 4.42E+04 | 4.25E+04 | 3.86E+04 | 4.06E+04 | 5.51E+04 | 2.86E+04 | 4.19E+04 | 1.26E+05 | 1.39E+05 | 1.33E+05 |
| pme1806 | 3.67E+02 | 1.79E+02 | 3.64E+00 | 3.68E+02 | [M-H]- | - | Chlorogenic acid methyl ester | Quinate and its derivatives | 1.02E+06 | 9.45E+05 | 9.99E+05 | 9.43E+05 | 9.33E+05 | 8.88E+05 | 2.51E+06 | 2.51E+06 | 2.51E+06 | 1.65E+06 | 1.44E+06 | 1.55E+06 | 8.52E+05 | 1.15E+06 | 1.00E+06 |
| pme1824 | 1.53E+02 | 1.09E+02 | 2.48E+00 | 1.54E+02 | [M-H]- | C00230 | Protocatechuic acid | Catechin derivatives | 1.44E+05 | 1.22E+05 | 1.40E+05 | 1.31E+05 | 9.99E+04 | 1.24E+05 | 1.07E+05 | 9.59E+04 | 1.01E+05 | 1.04E+05 | 8.81E+04 | 9.61E+04 | 1.71E+05 | 1.14E+06 | 6.56E+05 |
| pme1828 | 1.18E+02 | 1.18E+02 | 7.90E-01 | 1.17E+02 | [M+H]+ | C00719 | Betaine | Alkaloids | 3.88E+06 | 6.65E+06 | 7.03E+06 | 6.08E+06 | 5.64E+06 | 6.81E+06 | 7.19E+06 | 8.06E+06 | 7.63E+06 | 7.33E+06 | 7.94E+06 | 7.64E+06 | 8.61E+06 | 8.59E+06 | 8.60E+06 |
| pme1830 | 1.17E+02 | 9.99E+01 | 1.35E+00 | 1.18E+02 | [M-H]- | C00042 | Succinic acid | Organic acids | 1.76E+07 | 1.71E+07 | 1.68E+07 | 1.63E+07 | 1.51E+07 | 1.41E+07 | 3.17E+07 | 3.14E+07 | 3.16E+07 | 1.73E+07 | 2.24E+07 | 1.99E+07 | 1.33E+07 | 1.81E+07 | 1.57E+07 |
| pme1846 | 1.79E+02 | 7.09E+01 | 7.70E-01 | 1.80E+02 | [M-H]- | C00031 | D(+)-Glucose | Carbohydrates | 1.22E+07 | 1.20E+07 | 1.08E+07 | 1.03E+07 | 1.02E+07 | 1.02E+07 | 1.09E+07 | 1.05E+07 | 1.07E+07 | 1.03E+07 | 1.04E+07 | 1.04E+07 | 1.08E+07 | 1.12E+07 | 1.10E+07 |
| pme1851 | 1.30E+02 | 8.80E+01 | 8.10E-01 | 1.31E+02 | [M-H]- | C00300 | Creatine | Organic acids | 7.36E+05 | 8.89E+05 | 7.31E+05 | 8.73E+05 | 8.58E+05 | 6.48E+05 | 8.04E+05 | 9.58E+05 | 8.81E+05 | 7.97E+05 | 8.94E+05 | 8.46E+05 | 7.14E+05 | 8.74E+05 | 7.94E+05 |
| pme1872 | 1.14E+02 | 8.59E+01 | 8.10E-01 | 1.15E+02 | [M-H]- | C00148 | L-Proline | Amino acids | 3.25E+05 | 2.68E+05 | 2.52E+05 | 2.55E+05 | 2.69E+05 | 2.44E+05 | 2.68E+05 | 2.41E+05 | 2.55E+05 | 2.38E+05 | 2.82E+05 | 2.60E+05 | 3.19E+05 | 2.90E+05 | 3.05E+05 |
| pme1894 | 1.83E+02 | 5.93E+01 | 7.60E-01 | 1.82E+02 | [M+H]+ | C00794 | D-Sorbitol | Alcohols and polyols | 2.20E+06 | 2.65E+06 | 2.50E+06 | 2.19E+06 | 2.08E+06 | 2.03E+06 | 1.78E+06 | 1.71E+06 | 1.75E+06 | 1.75E+06 | 1.84E+06 | 1.80E+06 | 1.85E+06 | 1.90E+06 | 1.88E+06 |
| pme1936 | 1.14E+02 | 1.14E+02 | 6.20E-01 | 1.13E+02 | [M+H]+ | C00791 | Creatinine | Organic acids | 5.84E+05 | 7.02E+05 | 6.98E+05 | 7.05E+05 | 7.18E+05 | 7.30E+05 | 5.63E+05 | 6.09E+05 | 5.86E+05 | 5.50E+05 | 5.40E+05 | 5.45E+05 | 6.18E+05 | 6.38E+05 | 6.28E+05 |
| pme1944 | 1.83E+02 | 1.11E+02 | 8.00E-01 | 1.82E+02 | [M+H]+ | C00392 | D-Mannitol | Alcohols and polyols | 1.06E+05 | 1.89E+05 | 1.39E+05 | 1.52E+05 | 1.41E+05 | 1.67E+05 | 1.98E+05 | 1.91E+05 | 1.95E+05 | 1.49E+05 | 1.64E+05 | 1.57E+05 | 1.38E+05 | 1.20E+05 | 1.29E+05 |
| pme1949 | 3.75E+02 | 2.55E+02 | 3.26E+00 | 3.76E+02 | [M-H]- | C00255 | Riboflavin | Vitamins | 5.14E+05 | 5.23E+05 | 5.05E+05 | 4.96E+05 | 4.83E+05 | 4.95E+05 | 9.42E+05 | 9.60E+05 | 9.51E+05 | 4.44E+05 | 6.18E+05 | 5.31E+05 | 6.01E+05 | 5.45E+05 | 5.73E+05 |
| pme1977 | 1.73E+02 | 8.30E+01 | 3.87E+00 | 1.74E+02 | [M-H]- | C08278 | Suberic acid | Organic acids | 4.09E+04 | 3.97E+04 | 4.44E+04 | 3.43E+04 | 4.68E+04 | 3.17E+04 | 6.09E+04 | 5.40E+04 | 5.75E+04 | 4.36E+04 | 4.73E+04 | 4.55E+04 | 1.04E+05 | 7.24E+04 | 8.82E+04 |
| pme1987 | 8.80E+01 | 8.82E+01 | 7.80E-01 | 8.90E+01 | [M-H]- | C00041 | L-Alanine | Amino acids | 4.14E+04 | 4.00E+04 | 3.96E+04 | 4.17E+04 | 3.48E+04 | 4.44E+04 | 4.47E+04 | 3.08E+04 | 3.78E+04 | 5.29E+04 | 5.72E+04 | 5.51E+04 | 6.92E+04 | 4.95E+04 | 5.94E+04 |
| pme2009 | 1.49E+02 | 7.28E+01 | 1.03E+00 | 1.50E+02 | [M-H]- | - | L-(+)-Tartaric acid | Organic acids | 1.76E+05 | 2.24E+05 | 1.78E+05 | 2.19E+05 | 1.65E+05 | 1.62E+05 | 1.57E+05 | 2.45E+05 | 2.01E+05 | 2.09E+05 | 2.02E+05 | 2.06E+05 | 2.16E+05 | 1.90E+05 | 2.03E+05 |
| pme2019 | 1.49E+02 | 5.89E+01 | 8.10E-01 | 1.50E+02 | [M-H]- | - | DL-Arabinose | Carbohydrates | 1.65E+06 | 1.88E+06 | 1.51E+06 | 1.76E+06 | 1.59E+06 | 1.46E+06 | 1.65E+06 | 1.61E+06 | 1.63E+06 | 1.87E+06 | 1.75E+06 | 1.81E+06 | 1.58E+06 | 1.57E+06 | 1.58E+06 |
| pme2024 | 1.77E+02 | 1.60E+02 | 1.63E+00 | 1.76E+02 | [M+H]+ | C00780 | serotonin | Tryptamine derivatives | 5.92E+06 | 8.51E+06 | 6.77E+06 | 8.28E+06 | 6.53E+06 | 7.08E+06 | 1.07E+07 | 1.02E+07 | 1.05E+07 | 6.01E+06 | 4.77E+06 | 5.39E+06 | 3.52E+06 | 5.50E+06 | 4.51E+06 |
| pme2033 | 1.33E+02 | 7.08E+01 | 8.50E-01 | 1.34E+02 | [M-H]- | C00497 | L(-)-Malic acid | Organic acids | 4.05E+06 | 4.35E+06 | 3.91E+06 | 4.20E+06 | 4.21E+06 | 3.72E+06 | 5.83E+06 | 6.08E+06 | 5.96E+06 | 4.40E+06 | 4.55E+06 | 4.48E+06 | 3.55E+06 | 4.36E+06 | 3.96E+06 |
| pme2036 | 1.91E+02 | 8.50E+01 | 9.20E-01 | 1.92E+02 | [M-H]- | C00296 | Quinic acid | Quinate and its derivatives | 8.34E+06 | 8.68E+06 | 9.14E+06 | 9.21E+06 | 8.88E+06 | 9.03E+06 | 8.19E+06 | 9.48E+06 | 8.84E+06 | 8.09E+06 | 9.04E+06 | 8.57E+06 | 7.97E+06 | 8.87E+06 | 8.42E+06 |
| pme2046 | 1.70E+02 | 9.60E+01 | 7.90E-01 | 1.69E+02 | [M+H]+ | - | 3-N-Methyl-L-histidine | Amino acid derivatives | 1.13E+06 | 1.27E+06 | 2.67E+06 | 1.73E+06 | 1.16E+06 | 2.31E+06 | 3.12E+06 | 3.40E+06 | 3.26E+06 | 3.20E+06 | 2.93E+06 | 3.07E+06 | 2.22E+06 | 2.46E+06 | 2.34E+06 |
| pme2050 | 1.91E+02 | 1.31E+02 | 8.60E-01 | 1.92E+02 | [M-H]- | C00158 | Citric acid | Organic acids | 5.89E+04 | 4.81E+04 | 3.46E+04 | 5.08E+04 | 4.92E+04 | 5.62E+04 | 5.30E+04 | 5.62E+04 | 5.46E+04 | 4.28E+04 | 4.00E+04 | 4.14E+04 | 4.39E+04 | 4.36E+04 | 4.38E+04 |
| pme2108 | 1.63E+02 | 1.03E+02 | 6.80E-01 | 1.62E+02 | [M+H]+ | C00318 | L-Carnitine | Others | 2.41E+04 | 2.03E+04 | 1.75E+04 | 1.65E+04 | 1.33E+04 | 7.49E+03 | 6.59E+03 | 8.24E+03 | 7.42E+03 | 8.24E+03 | 1.06E+04 | 9.42E+03 | 9.22E+03 | 8.24E+03 | 8.73E+03 |
| pme2111 | 1.75E+02 | 8.70E+01 | 8.50E-01 | 1.76E+02 | [M-H]- | C00072 | L-ascorbate | Vitamins | 5.83E+05 | 5.84E+05 | 5.27E+05 | 6.02E+05 | 5.37E+05 | 5.54E+05 | 4.25E+05 | 4.52E+05 | 4.39E+05 | 5.48E+05 | 5.08E+05 | 5.28E+05 | 6.62E+05 | 5.91E+05 | 6.27E+05 |
| pme2122 | 1.12E+02 | 9.46E+01 | 8.40E-01 | 1.11E+02 | [M+H]+ | C00388 | Histamine | Amino acid derivatives | 4.89E+06 | 7.29E+06 | 6.50E+06 | 8.63E+06 | 8.02E+06 | 1.06E+07 | 9.71E+06 | 7.29E+06 | 8.50E+06 | 7.52E+06 | 1.02E+07 | 8.86E+06 | 4.98E+06 | 5.60E+06 | 5.29E+06 |
| pme2129 | 1.31E+02 | 6.89E+01 | 3.58E+00 | 1.32E+02 | [M-H]- | - | (S)-(-)-2-Hydroxyisocaproic acid | Organic acids | 5.49E+06 | 5.43E+06 | 5.58E+06 | 5.61E+06 | 5.10E+06 | 5.06E+06 | 5.40E+06 | 5.35E+06 | 5.38E+06 | 4.12E+06 | 4.11E+06 | 4.12E+06 | 2.83E+06 | 4.81E+06 | 3.82E+06 |
| pme2142 | 1.73E+02 | 8.39E+01 | 1.17E+00 | 1.74E+02 | [M-H]- | C01047 | L-Theanine | Amino acids | 8.28E+03 | 1.11E+04 | 9.99E+03 | 8.77E+03 | 9.77E+03 | 9.99E+03 | 1.01E+04 | 1.07E+04 | 1.04E+04 | 4.64E+03 | 1.01E+04 | 7.37E+03 | 7.61E+03 | 8.44E+03 | 8.03E+03 |
| pme2167 | 1.57E+02 | 1.11E+02 | 1.18E+00 | 1.56E+02 | [M+H]+ | C00295 | Orotic acid | Vitamins | 4.25E+06 | 5.90E+06 | 6.28E+06 | 5.50E+06 | 5.91E+06 | 5.69E+06 | 6.36E+06 | 5.62E+06 | 5.99E+06 | 4.92E+06 | 5.72E+06 | 5.32E+06 | 5.81E+06 | 6.38E+06 | 6.10E+06 |
| pme2169 | 1.15E+02 | 9.79E+01 | 1.19E+00 | 1.16E+02 | [M-H]- | C00122 | Fumaric acid | Organic acids | 1.78E+06 | 1.81E+06 | 1.84E+06 | 1.90E+06 | 1.86E+06 | 1.66E+06 | 2.59E+06 | 2.92E+06 | 2.76E+06 | 1.84E+06 | 2.32E+06 | 2.08E+06 | 1.65E+06 | 2.27E+06 | 1.96E+06 |
| pme2237 | 1.81E+02 | 7.08E+01 | 7.60E-01 | 1.82E+02 | [M-H]- | C01697 | Dulcitol | Alcohols and polyols | 2.83E+04 | 2.67E+04 | 5.17E+04 | 3.42E+04 | 3.25E+04 | 3.09E+04 | 2.90E+04 | 2.98E+04 | 2.94E+04 | 3.28E+04 | 2.64E+04 | 2.96E+04 | 3.21E+04 | 2.68E+04 | 2.95E+04 |
| pme2246 | 3.01E+02 | 5.88E+01 | 3.89E+00 | 3.02E+02 | [M-H]- | C10788 | Ellagic acid | Others | 3.95E+04 | 3.65E+04 | 2.60E+04 | 3.24E+04 | 2.76E+04 | 1.59E+04 | 2.05E+04 | 1.77E+04 | 1.91E+04 | 1.95E+04 | 1.89E+04 | 1.92E+04 | 2.68E+04 | 2.04E+04 | 2.36E+04 |
| pme2253 | 1.77E+02 | 7.48E+01 | 7.90E-01 | 1.78E+02 | [M-H]- | C01040 | L-Gulonic-γ-lactone | Carbohydrates | 7.83E+05 | 6.10E+05 | 5.73E+05 | 7.45E+05 | 7.91E+05 | 3.93E+05 | 5.54E+05 | 4.89E+05 | 5.22E+05 | 4.66E+05 | 5.16E+05 | 4.91E+05 | 5.57E+05 | 6.01E+05 | 5.79E+05 |
| pme2256 | 1.51E+02 | 7.08E+01 | 7.70E-01 | 1.52E+02 | [M-H]- | C01904 | D-Arabitol | Alcohols and polyols | 3.06E+04 | 3.74E+04 | 2.74E+04 | 2.83E+04 | 3.65E+04 | 2.58E+04 | 3.91E+04 | 2.61E+04 | 3.26E+04 | 3.55E+04 | 3.54E+04 | 3.55E+04 | 3.39E+04 | 4.68E+04 | 4.04E+04 |
| pme2263 | 2.43E+02 | 1.66E+02 | 3.38E+00 | 2.44E+02 | [M-H]- | C00120 | Biotin | Vitamins | 1.48E+04 | 1.33E+04 | 1.41E+04 | 1.58E+04 | 1.34E+04 | 1.35E+04 | 1.12E+04 | 1.17E+04 | 1.15E+04 | 1.16E+04 | 1.19E+04 | 1.18E+04 | 8.32E+03 | 1.25E+04 | 1.04E+04 |
| pme2268 | 1.38E+02 | 1.38E+02 | 7.90E-01 | 1.37E+02 | [M+H]+ | C01004 | Trigonelline | Alkaloids | 3.41E+07 | 4.08E+07 | 4.00E+07 | 3.99E+07 | 4.00E+07 | 3.75E+07 | 3.53E+07 | 3.28E+07 | 3.41E+07 | 6.01E+07 | 3.55E+07 | 4.78E+07 | 4.34E+07 | 3.61E+07 | 3.98E+07 |
| pme2278 | 3.62E+02 | 7.89E+01 | 1.17E+00 | 3.63E+02 | [M-H]- | - | Guanosine monophosphate | Nucleotide and its derivates | 1.80E+05 | 2.53E+05 | 1.90E+05 | 2.56E+05 | 2.71E+05 | 1.94E+05 | 1.89E+05 | 2.02E+05 | 1.96E+05 | 9.79E+04 | 2.49E+05 | 1.73E+05 | 2.27E+05 | 1.17E+05 | 1.72E+05 |
| pme2319 | 3.01E+02 | 2.42E+02 | 5.75E+00 | 3.02E+02 | [M-H]- | C01709 | Hesperetin | Flavanone | 8.58E+04 | 8.26E+04 | 8.30E+04 | 8.71E+04 | 8.60E+04 | 8.31E+04 | 9.90E+04 | 9.53E+04 | 9.72E+04 | 7.86E+04 | 8.43E+04 | 8.15E+04 | 3.06E+04 | 4.39E+04 | 3.73E+04 |
| pme2344 | 2.31E+02 | 1.49E+02 | 6.16E+00 | 2.30E+02 | [M+H]+ | - | Dodecanedioic aicd | Organic acids | 1.76E+04 | 1.86E+04 | 1.99E+04 | 1.55E+04 | 1.59E+04 | 2.28E+04 | 1.99E+04 | 1.63E+04 | 1.81E+04 | 1.97E+04 | 1.56E+04 | 1.77E+04 | 2.51E+04 | 1.75E+04 | 2.13E+04 |
| pme2362 | 1.51E+02 | 1.05E+02 | 2.89E+00 | 1.52E+02 | [M-H]- | C01984 | Mandelic acid | Organic acids | 1.43E+04 | 2.09E+04 | 2.33E+04 | 2.69E+04 | 3.13E+04 | 2.58E+04 | 9.00E+00 | 9.00E+00 | 9.00E+00 | 9.00E+00 | 9.00E+00 | 9.00E+00 | 9.00E+00 | 9.00E+00 | 9.00E+00 |
| pme2366 | 1.22E+02 | 1.05E+02 | 2.26E+00 | 1.21E+02 | [M+H]+ | C05332 | Phenethylamine | Others | 1.48E+07 | 1.91E+07 | 1.92E+07 | 1.75E+07 | 2.07E+07 | 1.91E+07 | 2.62E+07 | 2.74E+07 | 2.68E+07 | 2.31E+07 | 2.86E+07 | 2.59E+07 | 2.17E+07 | 2.04E+07 | 2.11E+07 |
| pme2380 | 1.45E+02 | 8.08E+01 | 1.14E+00 | 1.46E+02 | [M-H]- | - | A-Ketoglutaric acid | Organic acids | 4.09E+04 | 3.36E+04 | 3.00E+04 | 3.21E+04 | 3.08E+04 | 3.36E+04 | 5.08E+04 | 5.08E+04 | 5.08E+04 | 4.25E+04 | 5.20E+04 | 4.73E+04 | 1.02E+05 | 4.16E+04 | 7.18E+04 |
| pme2427 | 2.22E+02 | 1.63E+02 | 2.61E+00 | 2.23E+02 | [M-H]- | - | N-Acetyl-L-tyrosine | Amino acid derivatives | 4.67E+05 | 4.47E+05 | 4.42E+05 | 4.50E+05 | 4.74E+05 | 4.12E+05 | 9.51E+05 | 9.77E+05 | 9.64E+05 | 6.42E+05 | 5.68E+05 | 6.05E+05 | 5.60E+05 | 6.82E+05 | 6.21E+05 |
| pme2433 | 1.06E+02 | 8.81E+01 | 7.90E-01 | 1.05E+02 | [M+H]+ | C06772 | Diethanolamine | Others | 1.27E+05 | 8.58E+04 | 1.63E+05 | 1.31E+05 | 1.63E+05 | 1.65E+05 | 4.80E+04 | 6.91E+04 | 5.86E+04 | 7.66E+04 | 1.89E+05 | 1.33E+05 | 9.11E+04 | 1.72E+05 | 1.32E+05 |
| pme2435 | 1.63E+02 | 7.08E+01 | 7.60E-01 | 1.64E+02 | [M-H]- | C01019 | L-Fucose | Carbohydrates | 1.35E+05 | 1.44E+05 | 1.38E+05 | 1.33E+05 | 1.37E+05 | 1.34E+05 | 1.11E+05 | 9.43E+04 | 1.03E+05 | 1.43E+05 | 9.12E+04 | 1.17E+05 | 1.27E+05 | 9.57E+04 | 1.11E+05 |
| pme2457 | 4.47E+02 | 2.85E+02 | 3.87E+00 | 4.48E+02 | [M-H]- | - | Luteolin 7-O-glucoside (Cynaroside) | Flavone | 3.30E+05 | 3.40E+05 | 3.26E+05 | 3.08E+05 | 3.29E+05 | 2.86E+05 | 2.41E+05 | 2.69E+05 | 2.55E+05 | 1.14E+05 | 8.75E+04 | 1.01E+05 | 1.05E+05 | 1.10E+05 | 1.08E+05 |
| pme2478 | 1.37E+02 | 1.37E+02 | 3.06E+00 | 1.38E+02 | [M-H]- | C16700 | Protocatechuic aldehyde | Catechin derivatives | 6.36E+04 | 7.39E+04 | 7.45E+04 | 7.48E+04 | 7.37E+04 | 6.18E+04 | 1.45E+05 | 1.38E+05 | 1.42E+05 | 5.98E+04 | 6.21E+04 | 6.10E+04 | 4.65E+04 | 5.38E+04 | 5.02E+04 |
| pme2527 | 1.33E+02 | 7.00E+01 | 7.80E-01 | 1.32E+02 | [M+H]+ | C00077 | L(+)-Ornithine | Amino acids | 7.60E+05 | 9.54E+05 | 8.23E+05 | 9.61E+05 | 9.31E+05 | 9.90E+05 | 4.67E+05 | 4.24E+05 | 4.46E+05 | 2.34E+04 | 4.72E+05 | 2.48E+05 | 5.69E+04 | 2.80E+05 | 1.68E+05 |
| pme2529 | 1.63E+02 | 1.63E+02 | 7.50E-01 | 1.64E+02 | [M-H]- | C07326 | "1,5-Anhydro-D-glucitol" | Alcohols and polyols | 2.09E+05 | 1.86E+05 | 1.84E+05 | 1.77E+05 | 1.64E+05 | 1.88E+05 | 1.56E+05 | 1.50E+05 | 1.53E+05 | 1.81E+05 | 1.40E+05 | 1.61E+05 | 1.79E+05 | 1.54E+05 | 1.67E+05 |
| pme2560 | 1.76E+02 | 8.80E+01 | 1.27E+00 | 1.75E+02 | [M+H]+ | C01042 | N-Acetylaspartate | Amino acid derivatives | 1.20E+05 | 1.71E+05 | 1.67E+05 | 1.31E+05 | 1.62E+05 | 1.53E+05 | 2.31E+05 | 2.37E+05 | 2.34E+05 | 2.10E+05 | 1.97E+05 | 2.04E+05 | 1.25E+05 | 1.31E+05 | 1.28E+05 |
| pme2569 | 2.19E+02 | 1.56E+02 | 1.16E+00 | 2.18E+02 | [M+H]+ | C03740 | (5-L-Glutamyl)-L-amino acid | Amino acid derivatives | 7.11E+04 | 1.01E+05 | 7.88E+04 | 7.78E+04 | 9.11E+04 | 1.06E+05 | 1.08E+05 | 1.09E+05 | 1.09E+05 | 8.18E+04 | 1.01E+05 | 9.14E+04 | 8.93E+04 | 7.90E+04 | 8.42E+04 |
| pme2594 | 1.82E+02 | 1.38E+02 | 1.66E+00 | 1.83E+02 | [M-H]- | C00847 | 4-Pyridoxic acid | Pyridine derivatives | 1.40E+06 | 1.34E+06 | 1.31E+06 | 1.17E+06 | 1.16E+06 | 1.21E+06 | 1.54E+06 | 1.36E+06 | 1.45E+06 | 1.85E+06 | 1.12E+06 | 1.49E+06 | 9.71E+05 | 1.25E+06 | 1.11E+06 |
| pme2601 | 8.90E+01 | 6.89E+01 | 1.19E+00 | 9.00E+01 | [M-H]- | C01013 | 3-Hydroxypropanoic acid | Organic acids | 1.13E+05 | 1.45E+05 | 1.12E+05 | 1.58E+05 | 1.37E+05 | 1.25E+05 | 1.57E+05 | 1.89E+05 | 1.73E+05 | 1.30E+05 | 1.53E+05 | 1.42E+05 | 1.29E+05 | 1.55E+05 | 1.42E+05 |
| pme2614 | 1.64E+02 | 6.28E+01 | 7.60E-01 | 1.65E+02 | [M-H]- | - | Methionine sulfoxide | Amino acid derivatives | 1.37E+04 | 1.72E+04 | 1.39E+04 | 8.71E+03 | 1.48E+04 | 7.13E+03 | 9.23E+03 | 1.56E+04 | 1.24E+04 | 2.21E+04 | 1.98E+04 | 2.10E+04 | 1.32E+04 | 2.39E+04 | 1.86E+04 |
| pme2634 | 1.18E+02 | 8.80E+01 | 8.50E-01 | 1.17E+02 | [M+H]+ | C01826 | Dl-Norvaline | Amino acids | 8.05E+06 | 9.98E+06 | 9.44E+06 | 9.46E+06 | 9.61E+06 | 8.94E+06 | 7.02E+06 | 7.33E+06 | 7.18E+06 | 6.27E+06 | 7.97E+06 | 7.12E+06 | 1.03E+07 | 1.04E+07 | 1.04E+07 |
| pme2636 | 3.01E+02 | 2.53E+02 | 4.66E+00 | 3.02E+02 | [M-H]- | C18166 | Enterodiol | Alcohols and polyols | 9.20E+03 | 1.18E+04 | 1.20E+04 | 1.09E+04 | 7.88E+03 | 1.06E+04 | 7.08E+03 | 4.35E+03 | 5.72E+03 | 9.00E+00 | 4.31E+03 | 2.16E+03 | 5.07E+03 | 7.51E+03 | 6.29E+03 |
| pme2651 | 7.42E+02 | 6.20E+02 | 1.15E+00 | 7.43E+02 | [M-H]- | C00006 | NADP | Others | 2.48E+05 | 3.16E+05 | 3.13E+05 | 3.82E+05 | 3.39E+05 | 2.71E+05 | 3.46E+04 | 3.45E+04 | 3.46E+04 | 1.76E+05 | 6.69E+04 | 1.21E+05 | 1.72E+05 | 2.66E+05 | 2.19E+05 |
| pme2662 | 3.85E+02 | 2.44E+02 | 5.46E+00 | 3.84E+02 | [M+H]+ | C05443 | Vitamin D3 | Vitamins | 1.42E+04 | 1.17E+04 | 9.58E+03 | 1.16E+04 | 1.17E+04 | 1.17E+04 | 7.40E+03 | 5.45E+03 | 6.43E+03 | 5.75E+03 | 6.75E+03 | 6.25E+03 | 5.58E+03 | 5.97E+03 | 5.78E+03 |
| pme2670 | 1.21E+02 | 9.19E+01 | 3.72E+00 | 1.22E+02 | [M-H]- | C00633 | 4-Hydroxybenzaldehyde | Benzoic acid derivatives | 5.35E+05 | 5.96E+05 | 5.98E+05 | 5.98E+05 | 5.51E+05 | 5.82E+05 | 5.19E+05 | 5.12E+05 | 5.16E+05 | 3.22E+05 | 3.92E+05 | 3.57E+05 | 3.51E+05 | 3.97E+05 | 3.74E+05 |
| pme2693 | 1.31E+02 | 7.19E+01 | 8.00E-01 | 1.30E+02 | [M+H]+ | C02714 | N-Acetylputrescine | Phenolamides | 1.50E+05 | 1.63E+05 | 1.96E+05 | 1.35E+05 | 1.65E+05 | 1.84E+05 | 1.77E+05 | 1.89E+05 | 1.83E+05 | 1.81E+05 | 1.37E+05 | 1.59E+05 | 1.53E+05 | 1.69E+05 | 1.61E+05 |
| pme2698 | 3.13E+02 | 1.20E+02 | 3.62E+00 | 3.12E+02 | [M+H]+ | - | Phe-Phe | Amino acid derivatives | 3.49E+04 | 3.98E+04 | 4.70E+04 | 3.53E+04 | 2.86E+04 | 2.50E+04 | 4.08E+04 | 3.18E+04 | 3.63E+04 | 1.31E+04 | 2.26E+04 | 1.79E+04 | 3.33E+04 | 3.58E+04 | 3.46E+04 |
| pme2702 | 1.44E+02 | 1.02E+02 | 1.62E+00 | 1.45E+02 | [M-H]- | C02946 | 4-Acetamidobutyric acid | Organic acids | 2.08E+05 | 2.85E+05 | 2.08E+05 | 3.09E+05 | 2.66E+05 | 2.20E+05 | 3.26E+05 | 4.27E+05 | 3.77E+05 | 4.13E+05 | 3.91E+05 | 4.02E+05 | 1.72E+05 | 3.01E+05 | 2.37E+05 |
| pme2706 | 1.53E+02 | 1.09E+02 | 2.42E+00 | 1.54E+02 | [M-H]- | C00196 | "2,3-Dihydroxybenzoic acid" | Organic acids | 1.85E+05 | 1.44E+05 | 1.38E+05 | 1.51E+05 | 1.55E+05 | 1.16E+05 | 1.09E+05 | 1.00E+05 | 1.05E+05 | 1.40E+05 | 6.84E+04 | 1.04E+05 | 1.73E+05 | 1.18E+06 | 6.77E+05 |
| pme2723 | 1.46E+02 | 1.18E+02 | 4.54E+00 | 1.45E+02 | [M+H]+ | C08493 | Indole-3-carboxaldehyde | Indole derivatives | 2.59E+05 | 3.41E+05 | 3.18E+05 | 2.69E+05 | 3.65E+05 | 3.71E+05 | 4.89E+05 | 4.90E+05 | 4.90E+05 | 1.49E+05 | 2.90E+05 | 2.20E+05 | 1.19E+05 | 1.61E+05 | 1.40E+05 |
| pme2746 | 7.84E+02 | 4.37E+02 | 2.79E+00 | 7.85E+02 | [M-H]- | C00016 | Flavin adenine dinucleotide (FAD) | Nucleotide and its derivates | 2.85E+05 | 2.54E+05 | 2.58E+05 | 1.72E+05 | 2.06E+05 | 1.34E+05 | 1.00E+05 | 1.09E+05 | 1.05E+05 | 1.05E+05 | 1.10E+05 | 1.08E+05 | 1.55E+05 | 2.03E+05 | 1.79E+05 |
| pme2755 | 2.22E+02 | 1.38E+02 | 6.20E-01 | 2.21E+02 | [M+H]+ | C00140 | N-Acetyl-D-glucosamine | Carbohydrates | 6.08E+06 | 7.13E+06 | 6.61E+06 | 7.92E+06 | 8.00E+06 | 8.15E+06 | 7.38E+06 | 7.36E+06 | 7.37E+06 | 7.90E+06 | 8.10E+06 | 8.00E+06 | 7.87E+06 | 9.78E+06 | 8.83E+06 |
| pme2758 | 1.62E+02 | 1.44E+02 | 7.30E-01 | 1.63E+02 | [M-H]- | C03079 | 4-Hydroxy-L-glutamic acid | Amino acid derivatives | 1.94E+04 | 1.26E+04 | 1.41E+04 | 7.28E+03 | 1.24E+04 | 1.35E+04 | 1.09E+04 | 1.41E+04 | 1.25E+04 | 1.20E+04 | 1.30E+04 | 1.25E+04 | 1.50E+04 | 2.47E+04 | 1.99E+04 |
| pme2761 | 1.61E+02 | 1.61E+02 | 9.50E-01 | 1.62E+02 | [M-H]- | C01127 | 4-Hydroxy-2-oxoglutaric acid | Organic acids | 6.26E+05 | 6.24E+05 | 7.28E+05 | 5.55E+05 | 6.15E+05 | 5.28E+05 | 6.29E+05 | 6.37E+05 | 6.33E+05 | 6.57E+05 | 5.36E+05 | 5.97E+05 | 6.24E+05 | 6.10E+05 | 6.17E+05 |
| pme2786 | 2.19E+02 | 1.60E+02 | 3.32E+00 | 2.18E+02 | [M+H]+ | C00978 | N-Acetyl-5-hydroxytryptamine | Tryptamine derivatives | 9.03E+05 | 8.18E+05 | 9.61E+05 | 8.28E+05 | 9.48E+05 | 8.82E+05 | 4.77E+05 | 4.30E+05 | 4.54E+05 | 5.60E+05 | 5.24E+05 | 5.42E+05 | 9.11E+05 | 7.23E+05 | 8.17E+05 |
| pme2798 | 1.65E+02 | 1.50E+02 | 1.85E+00 | 1.66E+02 | [M-H]- | C16353 | 7-Methylxanthine | Nucleotide and its derivates | 2.87E+04 | 2.80E+04 | 2.29E+04 | 2.44E+04 | 2.38E+04 | 1.87E+04 | 9.00E+00 | 9.00E+00 | 9.00E+00 | 9.00E+00 | 9.00E+00 | 9.00E+00 | 9.00E+00 | 9.00E+00 | 9.00E+00 |
| pme2819 | 2.87E+02 | 1.73E+02 | 1.11E+01 | 2.86E+02 | [M+H]+ | - | "All-trans-13,14-dihydroretinol" | Vitamins | 2.68E+04 | 1.81E+04 | 2.24E+04 | 1.99E+04 | 1.89E+04 | 2.21E+04 | 1.62E+04 | 1.46E+04 | 1.54E+04 | 2.36E+04 | 2.33E+04 | 2.35E+04 | 1.32E+04 | 2.15E+04 | 1.74E+04 |
| pme2827 | 2.39E+02 | 2.23E+02 | 6.77E+00 | 2.40E+02 | [M-H]- | C00517 | Palmitaldehyde | Lipids_Fatty acids | 1.32E+07 | 1.39E+07 | 1.43E+07 | 1.49E+07 | 1.51E+07 | 1.57E+07 | 5.17E+06 | 7.83E+06 | 6.50E+06 | 5.45E+06 | 7.08E+06 | 6.27E+06 | 4.39E+06 | 6.56E+06 | 5.48E+06 |
| pme2828 | 1.40E+02 | 9.89E+01 | 6.30E-01 | 1.39E+02 | [M+H]+ | C00870 | 4-Nitrophenol | Others | 4.15E+07 | 4.74E+07 | 4.37E+07 | 4.36E+07 | 3.90E+07 | 3.57E+07 | 1.90E+07 | 1.77E+07 | 1.84E+07 | 2.62E+07 | 1.86E+07 | 2.24E+07 | 2.37E+07 | 2.19E+07 | 2.28E+07 |
| pme2830 | 1.40E+02 | 7.85E+01 | 7.50E-01 | 1.41E+02 | [M-H]- | - | O-Phosphorylethanolamine | Others | 2.38E+05 | 2.76E+05 | 2.44E+05 | 2.54E+05 | 2.55E+05 | 2.44E+05 | 1.75E+05 | 1.88E+05 | 1.82E+05 | 2.04E+05 | 2.37E+05 | 2.21E+05 | 1.36E+05 | 1.87E+05 | 1.62E+05 |
| pme2884 | 1.66E+02 | 1.24E+02 | 8.50E-01 | 1.67E+02 | [M-H]- | - | Guanidinoethyl sulfonate | Organic acids | 3.12E+04 | 2.92E+04 | 2.87E+04 | 3.47E+04 | 3.11E+04 | 3.07E+04 | 2.21E+04 | 3.34E+04 | 2.78E+04 | 3.66E+04 | 4.89E+04 | 4.28E+04 | 4.11E+04 | 2.61E+04 | 3.36E+04 |
| pme2890 | 2.69E+02 | 1.34E+02 | 8.50E-01 | 2.68E+02 | [M+H]+ | C01817 | L-Homocystine | Amino acids | 2.05E+04 | 1.89E+04 | 1.91E+04 | 1.97E+04 | 1.73E+04 | 2.06E+04 | 1.92E+04 | 1.42E+04 | 1.67E+04 | 1.71E+04 | 1.56E+04 | 1.64E+04 | 4.30E+04 | 5.83E+03 | 2.44E+04 |
| pme2895 | 3.19E+02 | 1.93E+02 | 3.52E+00 | 3.20E+02 | [M-H]- | C02906 | Dihydromyricetin | Flavonol | 9.02E+04 | 8.82E+04 | 8.23E+04 | 8.28E+04 | 7.18E+04 | 6.22E+04 | 1.05E+05 | 8.36E+04 | 9.43E+04 | 9.21E+04 | 8.89E+04 | 9.05E+04 | 1.10E+05 | 1.09E+05 | 1.10E+05 |
| pme2901 | 3.53E+02 | 1.91E+02 | 2.38E+00 | 3.54E+02 | [M-H]- | - | 1-O-Caffeoyl quinic acid | Quinate and its derivatives | 1.74E+06 | 1.70E+06 | 1.56E+06 | 1.60E+06 | 1.64E+06 | 1.69E+06 | 2.69E+06 | 2.42E+06 | 2.56E+06 | 2.40E+06 | 2.38E+06 | 2.39E+06 | 1.74E+06 | 1.67E+06 | 1.71E+06 |
| pme2903 | 1.37E+02 | 9.25E+01 | 3.10E+00 | 1.38E+02 | [M-H]- | C00156 | 4-Hydroxybenzoic acid | Organic acids | 4.80E+06 | 4.98E+06 | 4.82E+06 | 4.85E+06 | 4.77E+06 | 4.59E+06 | 8.27E+06 | 8.01E+06 | 8.14E+06 | 7.91E+06 | 9.72E+06 | 8.82E+06 | 1.33E+07 | 8.43E+06 | 1.09E+07 |
| pme2914 | 1.61E+02 | 5.68E+01 | 1.59E+00 | 1.62E+02 | [M-H]- | C03761 | "3-Hydroxy-3-methylpentane-1,5-dioic acid" | Amino acid derivatives | 1.37E+05 | 1.97E+05 | 9.47E+04 | 1.96E+05 | 1.73E+05 | 1.20E+05 | 4.15E+04 | 1.32E+05 | 8.68E+04 | 1.53E+05 | 2.25E+05 | 1.89E+05 | 2.87E+05 | 1.45E+05 | 2.16E+05 |
| pme2957 | 2.71E+02 | 1.51E+02 | 5.57E+00 | 2.72E+02 | [M-H]- | C06561 | Naringenin chalcone | Flavanone | 8.15E+05 | 8.63E+05 | 8.27E+05 | 7.69E+05 | 7.63E+05 | 6.77E+05 | 1.43E+06 | 1.76E+06 | 1.60E+06 | 1.86E+06 | 1.39E+06 | 1.63E+06 | 6.04E+05 | 1.40E+06 | 1.00E+06 |
| pme2963 | 2.87E+02 | 1.25E+02 | 4.62E+00 | 2.88E+02 | [M-H]- | C00974 | Aromadedrin (Dihydrokaempferol) | Flavonol | 2.72E+06 | 2.83E+06 | 2.82E+06 | 2.80E+06 | 2.81E+06 | 2.67E+06 | 2.40E+06 | 2.44E+06 | 2.42E+06 | 1.21E+06 | 9.83E+05 | 1.10E+06 | 8.49E+05 | 1.27E+06 | 1.06E+06 |
| pme2991 | 1.91E+02 | 1.76E+02 | 4.15E+00 | 1.92E+02 | [M-H]- | C01752 | Scopoletin (7-Hydroxy-5-methoxycoumarin) | Coumarins | 5.50E+06 | 5.51E+06 | 5.51E+06 | 5.40E+06 | 5.41E+06 | 5.10E+06 | 9.58E+06 | 9.49E+06 | 9.54E+06 | 2.68E+06 | 6.06E+06 | 4.37E+06 | 2.59E+06 | 3.13E+06 | 2.86E+06 |
| pme3007 | 4.03E+02 | 1.59E+02 | 7.00E-01 | 4.04E+02 | [M-H]- | C00015 | Uridine 5’-diphosphate | Nucleotide and its derivates | 1.82E+05 | 1.92E+05 | 2.27E+05 | 2.19E+05 | 1.88E+05 | 1.88E+05 | 1.29E+05 | 1.37E+05 | 1.33E+05 | 1.47E+05 | 1.44E+05 | 1.46E+05 | 1.10E+05 | 1.59E+05 | 1.35E+05 |
| pme3009 | 1.75E+02 | 9.89E+01 | 6.30E-01 | 1.74E+02 | [M+H]+ | - | trans-Citridic acid | Organic acids | 1.32E+05 | 1.09E+05 | 1.06E+05 | 1.24E+05 | 1.29E+05 | 9.29E+04 | 5.15E+04 | 4.28E+04 | 4.72E+04 | 2.13E+04 | 9.35E+04 | 5.74E+04 | 4.95E+04 | 4.68E+04 | 4.82E+04 |
| pme3011 | 1.04E+02 | 8.70E+01 | 7.70E-01 | 1.03E+02 | [M+H]+ | C00334 | γ-aminobutyric acid | Organic acids | 5.55E+04 | 7.50E+04 | 7.22E+04 | 6.73E+04 | 7.24E+04 | 5.03E+04 | 5.96E+04 | 5.52E+04 | 5.74E+04 | 5.68E+04 | 5.59E+04 | 5.64E+04 | 5.98E+04 | 5.11E+04 | 5.55E+04 |
| pme3017 | 1.04E+02 | 8.59E+01 | 7.60E-01 | 1.03E+02 | [M+H]+ | C03665 | 2-Aminoisobutyric acid | Amino acid derivatives | 1.32E+07 | 1.85E+07 | 1.92E+07 | 1.84E+07 | 1.79E+07 | 1.80E+07 | 1.89E+07 | 1.85E+07 | 1.87E+07 | 1.95E+07 | 1.61E+07 | 1.78E+07 | 1.67E+07 | 1.47E+07 | 1.57E+07 |
| pme3033 | 1.04E+02 | 5.80E+01 | 7.60E-01 | 1.03E+02 | [M+H]+ | C01026 | "N,N-Dimethylglycine" | Amino acid derivatives | 7.40E+05 | 9.04E+05 | 9.29E+05 | 8.88E+05 | 8.75E+05 | 8.43E+05 | 8.96E+05 | 8.84E+05 | 8.90E+05 | 9.84E+05 | 8.04E+05 | 8.94E+05 | 7.68E+05 | 7.19E+05 | 7.44E+05 |
| pme3038 | 1.30E+02 | 8.39E+01 | 8.50E-01 | 1.29E+02 | [M+H]+ | C01879 | 5-oxoproline | Amino acid derivatives | 8.77E+06 | 1.33E+07 | 1.23E+07 | 1.12E+07 | 1.33E+07 | 1.13E+07 | 7.65E+06 | 7.18E+06 | 7.42E+06 | 6.03E+06 | 8.74E+06 | 7.39E+06 | 3.48E+06 | 7.98E+06 | 5.73E+06 |
| pme3069 | 5.56E+02 | 3.71E+02 | 1.03E+01 | 5.55E+02 | [M+H]+ | C05122 | Taurocholic acid | Organic acids | 7.07E+06 | 6.28E+06 | 6.15E+06 | 6.52E+06 | 6.65E+06 | 7.66E+06 | 6.72E+06 | 5.84E+06 | 6.28E+06 | 5.87E+06 | 6.08E+06 | 5.98E+06 | 5.78E+06 | 5.78E+06 | 5.78E+06 |
| pme3081 | 1.69E+02 | 1.34E+02 | 7.70E-01 | 1.68E+02 | [M+H]+ | C00534 | 4-(Aminomethyl)-5-(hydroxymethyl)-2-methylpyridin-3-ol | Others | 4.80E+04 | 7.65E+04 | 1.32E+05 | 1.09E+05 | 1.02E+05 | 9.40E+04 | 1.04E+05 | 1.50E+05 | 1.27E+05 | 1.48E+05 | 1.50E+05 | 1.49E+05 | 1.12E+05 | 9.65E+04 | 1.04E+05 |
| pme3083 | 1.64E+02 | 9.20E+01 | 3.87E+00 | 1.65E+02 | [M-H]- | - | 2-(Formylamino)benzoic acid | Organic acids | 5.37E+06 | 5.42E+06 | 5.38E+06 | 5.30E+06 | 5.11E+06 | 5.08E+06 | 4.86E+06 | 4.80E+06 | 4.83E+06 | 1.25E+06 | 4.33E+06 | 2.79E+06 | 5.81E+06 | 4.87E+06 | 5.34E+06 |
| pme3096 | 1.18E+02 | 7.39E+01 | 1.34E+00 | 1.19E+02 | [M-H]- | C00872 | Aminomalonic acid | Organic acids | 1.19E+06 | 1.17E+06 | 1.19E+06 | 1.11E+06 | 1.04E+06 | 9.50E+05 | 1.56E+06 | 1.56E+06 | 1.56E+06 | 1.01E+06 | 1.31E+06 | 1.16E+06 | 8.44E+05 | 1.21E+06 | 1.03E+06 |
| pme3104 | 1.50E+02 | 1.33E+02 | 1.15E+00 | 1.49E+02 | [M+H]+ | C02216 | 1-Methyladenine | Nucleotide and its derivates | 2.32E+06 | 2.46E+06 | 2.51E+06 | 2.23E+06 | 2.64E+06 | 2.34E+06 | 2.24E+06 | 2.03E+06 | 2.14E+06 | 2.95E+06 | 2.30E+06 | 2.63E+06 | 2.79E+06 | 2.64E+06 | 2.72E+06 |
| pme3123 | 2.09E+02 | 1.79E+02 | 3.88E+00 | 2.10E+02 | [M-H]- | C02325 | Sinapyl alcohol | Hydroxycinnamoyl derivatives | 2.67E+04 | 3.31E+04 | 3.15E+04 | 3.19E+04 | 3.26E+04 | 2.45E+04 | 1.65E+04 | 1.26E+04 | 1.46E+04 | 4.03E+04 | 2.30E+04 | 3.17E+04 | 1.82E+04 | 2.61E+04 | 2.22E+04 |
| pme3129 | 4.63E+02 | 3.01E+02 | 3.86E+00 | 4.64E+02 | [M-H]- | - | Quercetin 4'-O-glucoside (Spiraeoside) | Flavonol | 1.59E+06 | 1.64E+06 | 1.46E+06 | 1.50E+06 | 1.43E+06 | 1.39E+06 | 1.15E+06 | 1.05E+06 | 1.10E+06 | 7.14E+05 | 6.99E+05 | 7.07E+05 | 1.34E+06 | 1.69E+06 | 1.52E+06 |
| pme3140 | 1.75E+02 | 1.31E+02 | 4.11E+00 | 1.76E+02 | [M-H]- | - | 6-Hydroxy-4-methylcoumarin | Coumarins | 6.88E+05 | 6.93E+05 | 6.79E+05 | 6.72E+05 | 6.80E+05 | 6.29E+05 | 7.84E+05 | 6.56E+05 | 7.20E+05 | 6.17E+05 | 6.58E+05 | 6.38E+05 | 5.16E+05 | 6.30E+05 | 5.73E+05 |
| pme3154 | 1.47E+02 | 5.88E+01 | 1.22E+00 | 1.48E+02 | [M-H]- | - | (Rs)-Mevalonic acid | Organic acids | 1.63E+05 | 5.33E+05 | 4.30E+05 | 3.76E+05 | 2.30E+05 | 3.73E+05 | 2.69E+05 | 5.63E+05 | 4.16E+05 | 3.23E+05 | 2.50E+05 | 2.87E+05 | 4.41E+05 | 6.29E+05 | 5.35E+05 |
| pme3160 | 2.59E+02 | 9.69E+01 | 7.10E-01 | 2.60E+02 | [M-H]- | C00092 | D-Glucose 6-phosphate | Carbohydrates | 5.62E+06 | 5.55E+06 | 5.83E+06 | 6.08E+06 | 5.71E+06 | 5.75E+06 | 3.53E+06 | 3.52E+06 | 3.53E+06 | 3.87E+06 | 4.38E+06 | 4.13E+06 | 2.79E+06 | 3.21E+06 | 3.00E+06 |
| pme3163 | 2.89E+02 | 7.89E+01 | 1.07E+00 | 2.90E+02 | [M-H]- | - | D-Sedoheptuiose 7-phosphate | Carbohydrates | 1.59E+06 | 1.91E+06 | 2.09E+06 | 1.69E+06 | 1.71E+06 | 1.68E+06 | 2.88E+06 | 1.99E+06 | 2.44E+06 | 2.40E+06 | 1.30E+06 | 1.85E+06 | 1.84E+06 | 1.55E+06 | 1.70E+06 |
| pme3174 | 3.24E+02 | 1.12E+02 | 8.30E-01 | 3.23E+02 | [M+H]+ | C00055 | Cytidine 5'-monophosphate (Cytidylic acid) | Nucleotide and its derivates | 1.56E+05 | 1.35E+05 | 1.60E+05 | 1.44E+05 | 1.29E+05 | 1.50E+05 | 2.02E+05 | 1.91E+05 | 1.97E+05 | 9.97E+04 | 1.05E+05 | 1.02E+05 | 9.73E+04 | 5.06E+04 | 7.40E+04 |
| pme3179 | 1.79E+02 | 7.59E+01 | 8.60E-01 | 1.78E+02 | [M+H]+ | C01419 | CYS-GLY | Amino acid derivatives | 1.32E+04 | 1.91E+04 | 1.56E+04 | 1.66E+04 | 1.25E+04 | 2.50E+04 | 2.16E+03 | 6.84E+03 | 4.50E+03 | 3.99E+03 | 4.20E+03 | 4.10E+03 | 1.23E+04 | 5.80E+03 | 9.05E+03 |
| pme3184 | 3.32E+02 | 1.36E+02 | 1.17E+00 | 3.31E+02 | [M+H]+ | C00360 | 2'-Deoxyadenosine-5'-monophosphate | Nucleotide and its derivates | 1.61E+06 | 1.55E+06 | 1.74E+06 | 1.67E+06 | 1.83E+06 | 1.47E+06 | 2.17E+06 | 2.02E+06 | 2.10E+06 | 2.10E+06 | 1.93E+06 | 2.02E+06 | 1.36E+06 | 1.38E+06 | 1.37E+06 |
| pme3188 | 3.23E+02 | 7.88E+01 | 1.18E+00 | 3.24E+02 | [M-H]- | C00105 | Uridine 5'-monophosphate | Nucleotide and its derivates | 4.44E+05 | 5.35E+05 | 4.93E+05 | 5.84E+05 | 5.31E+05 | 4.37E+05 | 6.44E+05 | 6.21E+05 | 6.33E+05 | 2.72E+05 | 5.91E+05 | 4.32E+05 | 6.22E+05 | 3.57E+05 | 4.90E+05 |
| pme3194 | 1.18E+02 | 7.60E+01 | 1.02E+00 | 1.17E+02 | [M+H]+ | - | N-acetylglycine | Amino acid derivatives | 2.84E+06 | 6.00E+06 | 5.56E+06 | 6.24E+06 | 5.01E+06 | 6.14E+06 | 6.99E+06 | 6.40E+06 | 6.70E+06 | 6.54E+06 | 6.26E+06 | 6.40E+06 | 6.13E+06 | 6.88E+06 | 6.51E+06 |
| pme3198 | 1.53E+02 | 1.09E+02 | 2.75E+00 | 1.54E+02 | [M-H]- | - | "2,4-Dihydroxybenzoic acid" | Benzoic acid derivatives | 1.14E+05 | 1.24E+05 | 8.81E+04 | 1.07E+05 | 8.56E+04 | 1.03E+05 | 6.20E+04 | 7.52E+04 | 6.86E+04 | 1.23E+05 | 7.25E+04 | 9.78E+04 | 1.17E+05 | 1.04E+06 | 5.79E+05 |
| pme3200 | 7.40E+01 | 5.69E+01 | 7.10E-01 | 7.31E+01 | [M+H]+ | - | 1-methylguanidine | Nucleotide and its derivates | 1.67E+04 | 2.12E+04 | 1.15E+04 | 1.82E+04 | 1.97E+04 | 2.05E+04 | 9.00E+00 | 2.13E+04 | 1.07E+04 | 2.43E+03 | 2.52E+04 | 1.38E+04 | 4.86E+03 | 1.37E+04 | 9.28E+03 |
| pme3207 | 1.41E+02 | 5.87E+01 | 6.90E-01 | 1.42E+02 | [M-H]- | - | "trans,trans-Muconic acid" | Organic acids | 9.27E+06 | 9.08E+06 | 9.10E+06 | 9.39E+06 | 8.95E+06 | 8.52E+06 | 8.66E+06 | 9.72E+06 | 9.19E+06 | 8.53E+06 | 7.29E+06 | 7.91E+06 | 8.35E+06 | 6.33E+06 | 7.34E+06 |
| pme3208 | 4.45E+02 | 2.82E+02 | 3.54E+00 | 4.46E+02 | [M-H]- | C16195 | Glycitin | Isoflavone | 1.64E+05 | 1.50E+05 | 1.71E+05 | 1.67E+05 | 1.55E+05 | 1.43E+05 | 4.20E+04 | 4.22E+04 | 4.21E+04 | 4.16E+04 | 6.91E+04 | 5.54E+04 | 1.68E+05 | 2.90E+05 | 2.29E+05 |
| pme3210 | 4.33E+02 | 2.71E+02 | 4.01E+00 | 4.32E+02 | [M+H]+ | C09126 | Genistein 7-O-Glucoside (Genistin) | Isoflavone | 1.50E+06 | 1.38E+06 | 1.33E+06 | 1.17E+06 | 1.05E+06 | 1.03E+06 | 3.71E+05 | 3.08E+05 | 3.40E+05 | 5.15E+05 | 4.47E+05 | 4.81E+05 | 3.21E+05 | 2.08E+05 | 2.65E+05 |
| pme3211 | 4.63E+02 | 3.01E+02 | 3.86E+00 | 4.64E+02 | [M-H]- | C05623 | Quercetin 3-O-glucoside (Isotrifoliin) | Flavonol | 1.54E+06 | 1.64E+06 | 1.41E+06 | 1.54E+06 | 1.39E+06 | 1.34E+06 | 1.14E+06 | 1.14E+06 | 1.14E+06 | 6.90E+05 | 6.81E+05 | 6.86E+05 | 1.31E+06 | 1.79E+06 | 1.55E+06 |
| pme3224 | 5.77E+02 | 4.13E+02 | 3.67E+00 | 5.78E+02 | [M-H]- | C12628 | Vitexin 2''-O-beta-L-rhamnoside | Flavone C-glycosides | 3.86E+04 | 3.85E+04 | 4.58E+04 | 3.91E+04 | 3.04E+04 | 3.16E+04 | 1.12E+04 | 1.18E+04 | 1.15E+04 | 1.99E+04 | 1.22E+04 | 1.61E+04 | 8.44E+03 | 1.32E+04 | 1.08E+04 |
| pme3245 | 2.71E+02 | 1.37E+02 | 6.82E+00 | 2.70E+02 | [M+H]+ | C10503 | Medicarpin | Hydroxycinnamoyl derivatives | 1.16E+04 | 1.45E+04 | 1.48E+04 | 1.97E+04 | 2.01E+04 | 2.57E+04 | 2.56E+04 | 2.62E+04 | 2.59E+04 | 1.96E+04 | 2.34E+04 | 2.15E+04 | 1.60E+04 | 1.33E+04 | 1.47E+04 |
| pme3246 | 3.41E+02 | 1.79E+02 | 2.72E+00 | 3.42E+02 | [M-H]- | C00761 | Coniferin | Hydroxycinnamoyl derivatives | 4.65E+06 | 4.67E+06 | 4.44E+06 | 4.41E+06 | 4.34E+06 | 4.42E+06 | 5.51E+06 | 5.18E+06 | 5.35E+06 | 5.45E+06 | 4.52E+06 | 4.99E+06 | 3.98E+06 | 3.92E+06 | 3.95E+06 |
| pme3256 | 6.11E+02 | 3.03E+02 | 2.37E+00 | 6.11E+02 | Protonated | C16315 | Delphinidin 3-O-rutinoside (Tulipanin) | Anthocyanins | 1.05E+05 | 8.32E+04 | 6.56E+04 | 7.01E+04 | 5.22E+04 | 7.35E+04 | 3.28E+04 | 4.68E+04 | 3.98E+04 | 1.66E+05 | 1.02E+05 | 1.34E+05 | 1.36E+05 | 8.87E+04 | 1.12E+05 |
| pme3261 | 2.71E+02 | 2.15E+02 | 4.48E+00 | 2.70E+02 | [M+H]+ | C14314 | 6-Hydroxydaidzein | Isoflavone | 2.53E+04 | 3.05E+04 | 2.42E+04 | 2.36E+04 | 3.56E+04 | 2.99E+04 | 2.09E+04 | 2.17E+04 | 2.13E+04 | 1.29E+04 | 1.39E+04 | 1.34E+04 | 1.70E+04 | 9.00E+00 | 8.50E+03 |
| pme3268 | 4.49E+02 | 2.87E+02 | 3.87E+00 | 4.48E+02 | [M+H]+ | C12626 | Kaempferol 3-O-galactoside (Trifolin) | Flavonol | 4.89E+06 | 5.16E+06 | 5.45E+06 | 5.45E+06 | 4.92E+06 | 4.61E+06 | 3.61E+06 | 3.64E+06 | 3.63E+06 | 5.05E+06 | 3.05E+06 | 4.05E+06 | 4.40E+06 | 3.99E+06 | 4.20E+06 |
| pme3279 | 2.87E+02 | 2.17E+02 | 4.89E+00 | 2.86E+02 | [M+H]+ | C12134 | 2'-Hydroxygenistein | Isoflavone | 2.04E+04 | 2.16E+04 | 2.88E+04 | 2.69E+04 | 2.20E+04 | 1.82E+04 | 1.90E+04 | 2.80E+04 | 2.35E+04 | 1.55E+04 | 1.50E+04 | 1.53E+04 | 2.69E+04 | 2.45E+04 | 2.57E+04 |
| pme3297 | 4.33E+02 | 2.87E+02 | 4.49E+00 | 4.32E+02 | [M+H]+ | C16911 | Kaempferol 3-O-rhamnoside (Kaempferin) | Flavonol | 3.92E+05 | 3.82E+05 | 3.61E+05 | 3.12E+05 | 3.33E+05 | 3.32E+05 | 9.43E+04 | 1.13E+05 | 1.04E+05 | 1.05E+05 | 9.20E+04 | 9.85E+04 | 2.18E+05 | 3.25E+05 | 2.72E+05 |
| pme3305 | 1.49E+02 | 1.30E+02 | 3.67E+00 | 1.50E+02 | [M-H]- | C02646 | p-Coumaryl alcohol | Hydroxycinnamoyl derivatives | 2.68E+04 | 2.46E+04 | 2.84E+04 | 2.72E+04 | 2.18E+04 | 3.20E+04 | 2.85E+04 | 3.04E+04 | 2.95E+04 | 1.29E+04 | 5.24E+03 | 9.07E+03 | 4.88E+03 | 1.28E+04 | 8.84E+03 |
| pme3309 | 1.45E+02 | 1.01E+02 | 2.58E+00 | 1.46E+02 | [M-H]- | - | 2-Methylglutaric acid | Organic acids | 2.83E+05 | 2.87E+05 | 3.07E+05 | 2.87E+05 | 2.96E+05 | 2.86E+05 | 3.52E+05 | 3.22E+05 | 3.37E+05 | 2.91E+05 | 2.40E+05 | 2.66E+05 | 2.67E+05 | 2.75E+05 | 2.71E+05 |
| pme3313 | 2.59E+02 | 7.88E+01 | 8.30E-01 | 2.60E+02 | [M-H]- | C00085 | D-Fructose 6-phosphate | Carbohydrates | 1.94E+06 | 1.86E+06 | 1.86E+06 | 1.71E+06 | 1.55E+06 | 1.94E+06 | 1.22E+06 | 9.51E+05 | 1.09E+06 | 1.21E+06 | 1.26E+06 | 1.24E+06 | 9.21E+05 | 9.65E+05 | 9.43E+05 |
| pme3333 | 1.53E+02 | 1.36E+02 | 1.51E+00 | 1.52E+02 | [M+H]+ | - | "1,4-dihydro-1-Methyl-4-oxo-3-pyridinecarboxamide" | Pyridine derivatives | 9.69E+05 | 1.17E+06 | 1.07E+06 | 1.19E+06 | 1.14E+06 | 9.08E+05 | 1.70E+06 | 1.66E+06 | 1.68E+06 | 7.67E+05 | 1.23E+06 | 9.99E+05 | 9.42E+05 | 4.58E+03 | 4.73E+05 |
| pme3336 | 3.82E+02 | 1.34E+02 | 2.06E+00 | 3.83E+02 | [M-H]- | - | N6-Succinyl Adenosine | Nucleotide and its derivates | 1.63E+06 | 1.54E+06 | 1.79E+06 | 1.53E+06 | 1.51E+06 | 1.51E+06 | 1.88E+06 | 1.71E+06 | 1.80E+06 | 1.67E+06 | 1.54E+06 | 1.61E+06 | 1.34E+06 | 1.52E+06 | 1.43E+06 |
| pme3346 | 1.31E+02 | 7.08E+01 | 2.76E+00 | 1.32E+02 | [M-H]- | - | 5-hydroxyhexanoic acid | Organic acids | 2.25E+06 | 2.37E+06 | 2.24E+06 | 2.19E+06 | 2.18E+06 | 2.24E+06 | 1.82E+06 | 1.78E+06 | 1.80E+06 | 8.42E+05 | 9.80E+05 | 9.11E+05 | 1.51E+06 | 1.60E+06 | 1.56E+06 |
| pme3349 | 2.82E+02 | 1.92E+02 | 1.73E+00 | 2.83E+02 | [M-H]- | - | 8-Hydroxy-2-deoxyguanosine | Nucleotide and its derivates | 1.63E+04 | 1.71E+04 | 1.76E+04 | 1.61E+04 | 1.19E+04 | 1.47E+04 | 2.41E+04 | 2.59E+04 | 2.50E+04 | 1.78E+04 | 2.38E+04 | 2.08E+04 | 1.75E+04 | 1.29E+04 | 1.52E+04 |
| pme3351 | 1.44E+02 | 1.26E+02 | 8.40E-01 | 1.45E+02 | [M-H]- | C04076 | Allysine(6-Oxo DL-Norleucine) | Amino acid derivatives | 1.69E+04 | 2.41E+04 | 2.42E+04 | 3.28E+04 | 1.39E+04 | 2.38E+04 | 3.02E+04 | 2.61E+04 | 2.82E+04 | 3.12E+04 | 3.68E+04 | 3.40E+04 | 2.90E+04 | 2.86E+04 | 2.88E+04 |
| pme3384 | 1.62E+02 | 7.40E+01 | 1.16E+00 | 1.61E+02 | [M+H]+ | - | N-Acetylthreonine | Amino acid derivatives | 2.07E+05 | 2.74E+05 | 1.89E+05 | 2.43E+05 | 2.47E+05 | 1.70E+05 | 2.04E+05 | 1.88E+05 | 1.96E+05 | 3.31E+05 | 2.63E+05 | 2.97E+05 | 2.30E+05 | 1.27E+05 | 1.79E+05 |
| pme3388 | 1.89E+02 | 1.44E+02 | 7.50E-01 | 1.88E+02 | [M+H]+ | C01924 | H-HomoArg-OH | Amino acid derivatives | 1.28E+06 | 1.88E+06 | 2.11E+06 | 1.61E+06 | 1.69E+06 | 1.66E+06 | 2.13E+06 | 1.78E+06 | 1.96E+06 | 1.19E+06 | 1.46E+06 | 1.33E+06 | 1.68E+06 | 1.65E+06 | 1.67E+06 |
| pme3396 | 2.89E+02 | 2.15E+02 | 3.65E+00 | 2.88E+02 | [M+H]+ | C01378 | Fustin | Flavonol | 3.88E+04 | 2.79E+04 | 3.99E+04 | 4.92E+04 | 4.82E+04 | 3.65E+04 | 2.74E+04 | 3.52E+04 | 3.13E+04 | 3.74E+04 | 3.11E+04 | 3.43E+04 | 3.85E+04 | 2.04E+04 | 2.95E+04 |
| pme3416 | 1.77E+02 | 1.33E+02 | 3.24E+00 | 1.78E+02 | [M-H]- | C09263 | "Esculetin (6,7-dihydroxycoumarin)" | Coumarins | 4.18E+05 | 4.40E+05 | 4.37E+05 | 4.07E+05 | 4.17E+05 | 4.08E+05 | 7.39E+05 | 7.55E+05 | 7.47E+05 | 2.13E+05 | 3.94E+05 | 3.04E+05 | 2.51E+05 | 3.24E+05 | 2.88E+05 |
| pme3422 | 2.07E+02 | 1.92E+02 | 3.56E+00 | 2.08E+02 | [M-H]- | C09265 | "6-Methoxy-7,8-DihydroxyCoumarin" | Coumarins | 8.52E+04 | 7.59E+04 | 1.14E+05 | 7.89E+04 | 8.02E+04 | 8.62E+04 | 8.29E+04 | 8.89E+04 | 8.59E+04 | 1.01E+05 | 7.11E+04 | 8.61E+04 | 7.67E+04 | 6.65E+04 | 7.16E+04 |
| pme3428 | 3.39E+02 | 1.77E+02 | 2.72E+00 | 3.40E+02 | [M-H]- | C09264 | "Esculin (6,7-Dihydroxycoumarin-6-glucoside)" | Coumarins | 9.75E+06 | 9.55E+06 | 9.23E+06 | 9.30E+06 | 9.33E+06 | 9.14E+06 | 7.77E+06 | 7.53E+06 | 7.65E+06 | 8.83E+06 | 7.99E+06 | 8.41E+06 | 5.64E+06 | 6.30E+06 | 5.97E+06 |
| pme3439 | 2.71E+02 | 1.35E+02 | 5.49E+00 | 2.72E+02 | [M-H]- | C08578 | Butein | Flavanone | 4.84E+03 | 4.48E+03 | 3.92E+03 | 4.99E+03 | 4.54E+03 | 5.75E+03 | 6.40E+03 | 8.68E+03 | 7.54E+03 | 3.48E+03 | 3.95E+03 | 3.72E+03 | 2.99E+03 | 2.29E+03 | 2.64E+03 |
| pme3443 | 2.07E+02 | 1.77E+02 | 4.61E+00 | 2.08E+02 | [M-H]- | - | Sinapinaldehyde | Hydroxycinnamoyl derivatives | 1.94E+06 | 1.98E+06 | 1.97E+06 | 1.90E+06 | 1.91E+06 | 1.90E+06 | 1.72E+06 | 1.71E+06 | 1.72E+06 | 1.60E+06 | 1.50E+06 | 1.55E+06 | 1.26E+06 | 1.30E+06 | 1.28E+06 |
| pme3453 | 1.47E+02 | 1.29E+02 | 4.42E+00 | 1.48E+02 | [M-H]- | - | p-Coumaraldehyde | Hydroxycinnamoyl derivatives | 6.36E+03 | 8.53E+03 | 8.66E+03 | 7.93E+03 | 8.88E+03 | 8.61E+03 | 7.67E+03 | 2.96E+03 | 5.32E+03 | 2.94E+03 | 4.08E+03 | 3.51E+03 | 4.93E+03 | 6.17E+03 | 5.55E+03 |
| pme3461 | 3.01E+02 | 1.51E+02 | 5.75E+00 | 3.02E+02 | [M-H]- | C09756 | Homoeriodictyol | Flavanone | 6.49E+04 | 6.20E+04 | 6.82E+04 | 6.25E+04 | 6.30E+04 | 6.25E+04 | 7.51E+04 | 6.97E+04 | 7.24E+04 | 6.26E+04 | 6.36E+04 | 6.31E+04 | 2.69E+04 | 3.69E+04 | 3.19E+04 |
| pme3464 | 2.85E+02 | 1.64E+02 | 6.98E+00 | 2.86E+02 | [M-H]- | C05334 | Isosakuranetin (4'-Methylnaringenin) | Flavanone | 1.34E+04 | 1.34E+04 | 1.21E+04 | 1.41E+04 | 1.33E+04 | 1.34E+04 | 3.74E+04 | 3.00E+04 | 3.37E+04 | 9.00E+00 | 7.71E+04 | 3.86E+04 | 8.58E+03 | 1.89E+04 | 1.37E+04 |
| pme3468 | 7.39E+02 | 5.93E+02 | 3.35E+00 | 7.40E+02 | [M-H]- | C10178 | Kaempferol-3-O-robinoside-7-O-rhamnoside (Robinin) | Flavonol | 1.16E+04 | 9.60E+03 | 1.57E+04 | 1.16E+04 | 1.50E+04 | 1.07E+04 | 4.06E+03 | 4.30E+03 | 4.18E+03 | 5.50E+03 | 5.87E+03 | 5.69E+03 | 1.01E+04 | 1.07E+04 | 1.04E+04 |
| pme3473 | 2.71E+02 | 1.51E+02 | 5.59E+00 | 2.72E+02 | [M-H]- | C09614 | Butin | Flavone | 4.88E+06 | 5.00E+06 | 4.95E+06 | 4.71E+06 | 4.86E+06 | 4.90E+06 | 7.27E+06 | 7.77E+06 | 7.52E+06 | 3.17E+06 | 3.70E+06 | 3.44E+06 | 2.81E+06 | 2.98E+06 | 2.90E+06 |
| pme3478 | 2.77E+02 | 1.46E+02 | 2.84E+00 | 2.78E+02 | [M-H]- | C00831 | Pantetheine | Vitamins | 3.44E+04 | 3.16E+04 | 2.69E+04 | 2.35E+04 | 2.28E+04 | 2.38E+04 | 8.57E+04 | 8.13E+04 | 8.35E+04 | 1.75E+04 | 2.93E+04 | 2.34E+04 | 2.49E+04 | 3.05E+04 | 2.77E+04 |
| pme3514 | 3.01E+02 | 1.51E+02 | 5.11E+00 | 3.02E+02 | [M-H]- | C10105 | Morin | Flavonol | 4.79E+04 | 3.69E+04 | 3.69E+04 | 3.32E+04 | 2.93E+04 | 2.73E+04 | 4.35E+04 | 4.32E+04 | 4.34E+04 | 1.61E+04 | 2.17E+04 | 1.89E+04 | 2.07E+04 | 2.54E+04 | 2.31E+04 |
| pme3564 | 1.79E+02 | 1.79E+02 | 3.19E+00 | 1.78E+02 | [M+H]+ | C03093 | Daphnetin | Coumarins | 1.38E+05 | 1.40E+05 | 1.10E+05 | 8.07E+04 | 1.09E+05 | 1.04E+05 | 1.26E+05 | 1.58E+05 | 1.42E+05 | 7.03E+04 | 7.16E+04 | 7.10E+04 | 8.08E+04 | 9.62E+04 | 8.85E+04 |
| pme3569 | 2.07E+02 | 2.07E+02 | 4.77E+00 | 2.06E+02 | [M+H]+ | C09311 | Scoparone | Coumarins | 1.19E+05 | 1.12E+05 | 1.14E+05 | 1.25E+05 | 1.02E+05 | 9.81E+04 | 1.08E+05 | 1.11E+05 | 1.10E+05 | 4.38E+04 | 9.75E+04 | 7.07E+04 | 4.04E+04 | 8.19E+04 | 6.12E+04 |
| pme3576 | 1.38E+02 | 9.19E+01 | 4.07E+00 | 1.37E+02 | [M+H]+ | C00108 | Anthranilic acid | Benzoic acid derivatives | 5.86E+04 | 5.10E+04 | 6.81E+04 | 5.58E+04 | 3.79E+04 | 6.66E+04 | 6.03E+04 | 4.59E+04 | 5.31E+04 | 4.80E+04 | 1.88E+04 | 3.34E+04 | 3.06E+04 | 2.74E+04 | 2.90E+04 |
| pme3609 | 2.87E+02 | 2.32E+02 | 3.54E+00 | 2.87E+02 | Protonated | C05905 | Cyanidin | Anthocyanins | 2.04E+05 | 2.42E+05 | 1.85E+05 | 1.86E+05 | 1.99E+05 | 2.20E+05 | 6.97E+04 | 6.65E+04 | 6.81E+04 | 7.38E+04 | 7.11E+04 | 7.25E+04 | 8.67E+04 | 4.51E+04 | 6.59E+04 |
| pme3705 | 1.93E+02 | 7.29E+01 | 7.20E-01 | 1.94E+02 | [M-H]- | C00191 | D-glucoronic acid | Carbohydrates | 1.94E+06 | 2.03E+06 | 1.89E+06 | 2.03E+06 | 1.74E+06 | 1.99E+06 | 1.02E+06 | 8.12E+05 | 9.16E+05 | 2.77E+06 | 8.05E+05 | 1.79E+06 | 1.46E+06 | 9.45E+05 | 1.20E+06 |
| pme3719 | 1.65E+02 | 7.49E+01 | 7.50E-01 | 1.66E+02 | [M-H]- | - | D-Xylonic acid | Organic acids | 5.10E+06 | 4.87E+06 | 4.68E+06 | 4.65E+06 | 4.63E+06 | 4.24E+06 | 3.85E+06 | 3.83E+06 | 3.84E+06 | 3.68E+06 | 4.38E+06 | 4.03E+06 | 4.10E+06 | 4.32E+06 | 4.21E+06 |
| pme3732 | 2.44E+02 | 1.11E+02 | 8.30E-01 | 2.43E+02 | [M+H]+ | C00475 | Cytidine | Nucleotide and its derivates | 3.60E+06 | 3.55E+06 | 3.86E+06 | 3.13E+06 | 3.12E+06 | 3.66E+06 | 7.67E+06 | 6.87E+06 | 7.27E+06 | 3.93E+06 | 4.53E+06 | 4.23E+06 | 3.05E+06 | 2.88E+06 | 2.97E+06 |
| pme3827 | 1.98E+02 | 1.15E+02 | 1.16E+00 | 1.97E+02 | [M+H]+ | C00355 | "3,4-Dihydroxy-DL-phenylalanine" | Amino acid derivatives | 2.39E+06 | 1.86E+06 | 2.06E+06 | 1.85E+06 | 1.83E+06 | 1.75E+06 | 2.64E+06 | 2.16E+06 | 2.40E+06 | 1.28E+06 | 2.10E+06 | 1.69E+06 | 1.89E+06 | 1.42E+06 | 1.66E+06 |
| pme3835 | 3.44E+02 | 3.44E+02 | 1.33E+00 | 3.45E+02 | [M-H]- | C00942 | "Guanosine 3',5'-cyclic monophosphate" | Nucleotide and its derivates | 1.40E+06 | 1.63E+06 | 1.56E+06 | 1.75E+06 | 1.62E+06 | 1.43E+06 | 3.37E+06 | 3.74E+06 | 3.56E+06 | 7.65E+05 | 3.40E+06 | 2.08E+06 | 4.56E+06 | 2.59E+06 | 3.58E+06 |
| pme3960 | 2.50E+02 | 1.31E+02 | 1.88E+00 | 2.51E+02 | [M-H]- | C00559 | Deoxyadenosine | Nucleotide and its derivates | 1.25E+04 | 1.44E+04 | 1.78E+04 | 1.58E+04 | 1.72E+04 | 1.67E+04 | 8.66E+03 | 1.14E+04 | 1.00E+04 | 5.94E+03 | 4.13E+03 | 5.04E+03 | 4.19E+03 | 4.93E+03 | 4.56E+03 |
| pme3965 | 3.10E+02 | 1.78E+02 | 2.18E+00 | 3.11E+02 | [M-H]- | - | 2-(dimethylamino)guanosine | Nucleotide and its derivates | 6.35E+04 | 6.53E+04 | 6.33E+04 | 6.20E+04 | 6.68E+04 | 7.20E+04 | 7.97E+04 | 8.58E+04 | 8.28E+04 | 2.72E+04 | 5.61E+04 | 4.17E+04 | 1.88E+04 | 4.83E+04 | 3.36E+04 |
| pme3968 | 1.66E+02 | 1.49E+02 | 1.15E+00 | 1.65E+02 | [M+H]+ | C02242 | 7-methylguanine | Nucleotide and its derivates | 1.69E+04 | 2.82E+04 | 2.40E+04 | 2.36E+04 | 5.05E+04 | 2.52E+04 | 4.41E+04 | 4.04E+04 | 4.23E+04 | 1.67E+04 | 2.86E+04 | 2.27E+04 | 3.30E+04 | 1.99E+04 | 2.65E+04 |
| pme3970 | 2.43E+02 | 1.53E+02 | 1.21E+00 | 2.44E+02 | [M-H]- | C02067 | β-Pseudouridine | Nucleotide and its derivates | 2.95E+05 | 3.07E+05 | 2.71E+05 | 2.82E+05 | 2.75E+05 | 2.35E+05 | 5.41E+05 | 6.43E+05 | 5.92E+05 | 2.21E+05 | 4.62E+05 | 3.42E+05 | 1.86E+05 | 2.37E+05 | 2.12E+05 |
| pme3982 | 2.67E+02 | 1.35E+02 | 1.48E+00 | 2.68E+02 | [M-H]- | - | Hypoxanthine-9-β-D-arabinofuranoside | Nucleotide and its derivates | 4.35E+05 | 4.31E+05 | 4.00E+05 | 4.15E+05 | 4.14E+05 | 3.63E+05 | 5.31E+05 | 5.59E+05 | 5.45E+05 | 2.84E+05 | 5.13E+05 | 3.99E+05 | 2.85E+05 | 4.40E+05 | 3.63E+05 |
